# Using eDNA tools to examine the impact of kelp farming on underlying sediments

**DOI:** 10.1371/journal.pone.0331416

**Published:** 2025-09-05

**Authors:** Samuel H. Tan, Emmaeve Jourdain, Shane P. Farrell, Nichole N. Price, Jeremy J. Rich, David Emerson

**Affiliations:** 1 University of Maine Darling Marine Center, School of Marine Sciences, Walpole, Maine, United States of America; 2 Bigelow Laboratory for Ocean Sciences, East Boothbay, Maine, United States of America; 3 University of British Columbia, Vancouver, BC, Canada; Gujarat Institute of Desert Ecology, INDIA

## Abstract

Using environmental DNA (eDNA)-based tools, we examined sediments underlying a ~ 1.25 hectare commercial kelp farm in the Gulf of Maine growing sugar kelp (*Saccharina latissima*) for two farming seasons, post-harvest. Two eDNA methods were used: a newly designed *S. latissima*-specific digital polymerase chain reaction (dPCR) assay targeting the cytochrome oxidase subunit I (COI) mitochondrial gene, as well as metabarcoding for the 16S and 18S ribosomal RNA (rRNA) genes, to examine overall bacterial, archaeal, and eukaryotic diversity. Sediment carbon and nitrogen content was analyzed using isotope ratio mass spectrometry (IRMS) as more traditional indicators of potential kelp biomass-derived nutrient enrichment in the benthos. When targeted sampling sites were added inside the footprint of the farm lease area in year two of the study, dPCR data showed subtle but significant differences between sediment samples inside and outside of the farm, with mean *S. latissima* COI gene copies from cores taken inside the farm being ~8% greater than mean values outside the farm. The highest COI copy numbers in marine sediments were from sites with observed accumulation of kelp biomass, while there was no conclusive difference in carbon and nitrogen content of those same sediment samples. Metabarcoding data also revealed subtle differences in taxa associated with sediments inside and outside the farm. For example, microbial taxa that correlated with kelp eDNA from cores within the farm included the families *Rhodothermaceae, Rubritaleaceae, Flavobacteriaceae, Prolixibacteraceae, Nitrosomonadaceae, Nitrincolaceae* and *Rubinisphaeraceae*. However, the majority of the above taxa were low in relative abundance, with only *Flavobacteriaceae* ranking among the top 30 most abundant and prevalent families in these sediments. In summary, this study demonstrates the sensitivity and specificity of eDNA tools to detect potential ecological and anthropogenic effects in marine sediments, beyond that of bulk nutrient and stable isotope analyses.

## Introduction

Kelp aquaculture is a growing industry worldwide [1] and the fastest growing ocean farming sector [2,3]. Kelp aquaculture can serve as an important component of a more equitable blue economy, producing raw material for potentially environmentally sustainable products [4–6] (e.g., food and food additives, nutraceuticals, animal feed, fertilizers, bioplastics, biofuels). In Maine, the expanding kelp aquaculture sector is the largest in North America at present [7]. There is also the potential for kelp aquaculture to form an increasingly larger supplementary income source as marine resource harvesters (e.g., lobstermen) and aquaculturists find ways to diversify their fisheries activities in response to climate change [8].

Besides their value as a marine resource, large-scale kelp aquaculture also holds promise for bioremediation in the form of dissolved nutrient capture (e.g., organic and inorganic carbon and nitrogen from anthropogenic sources that might otherwise lead to marine eutrophication and acidification) [9–11], and marine carbon dioxide removal (mCDR) solutions. Uniquely, kelp aquaculture is also the only mCDR strategy that can also generate useful and valuable aquacultural products on a large scale. Given the scale and importance of the oceans and their importance in global carbon cycling [12–17], a diverse suite of mCDR solutions is likely to be necessary as part of a wider portfolio of CDR methods [18–21]; to keep global carbon dioxide levels manageable in the face of anthropogenic climate change [22].

Among macroalgae, kelp are especially promising candidates for large-scale mCDR, given that they are already widely grown in aquaculture, grow quickly, and are relatively structurally resilient to physical and chemical degradation compared to other macroalgae [23–29]. Kelp biomass can theoretically be sunk en masse into the deep sea, where it can remain in deep ocean circulation for hundreds of years [23,30,17], or even longer, if net burial in marine sediments lacking significant bioturbation can occur [31].

However, large-scale biomass sinking is still highly controversial for potential negative effects on the environment in exchange for uncertain benefits, particularly given the vulnerability of benthic habitats to disturbance, especially in the deep sea [5,32,33]. Nevertheless, when grown for aquacultural products, kelp farming is thought to have minimal environmental impact at present production scales [34,35]. These previous studies have focused primarily on benthic invertebrates, eelgrass, and sediment nutrient content. There is little known at present about the effects of kelp farming on the benthic epifaunal and infaunal microbiome, including both prokaryotes and eukaryotes. Such communities are known to be sensitive indicators of disturbance that may not necessarily be reflected in metazoan communities [36]. Furthermore, support of such environmental impact findings may be of interest to farmers who value the sustainability of their operations.

Even if not grown specifically for mCDR purposes, kelp aquaculture sheds large amounts of organic carbon, and presumably organic nitrogen, into the surrounding environment [12,37–39] as part of natural growth dynamics (e.g., blade fragmentation), environmental disturbances (e.g., storms), and farming operations (e.g., discarding of biofouled material and unwanted parts of the thallus, like holdfasts and stipes). Quantifying these fluxes and the fate of the affiliated carbon could represent a solution where biomass could potentially be verified for mCDR applications without compromising the production and sale of higher value-added products.

Regardless, whether for dedicated or incidental mCDR purposes, or a combination thereof (e.g., baling and depositing culled biomass), any mCDR method requires standardized, empirical, and quantitative measurement, monitoring, reporting and verification (MMRV), ideally in a full Forensic Carbon Accounting-based framework [40,41]. In the case of biomass sinking, highly specific, quantitative tracers are required to trace kelp biomass from sources to sinks [42,43], during which it is likely to undergo substantial transport and degradation. At present, traditional methods of tracking such biomass generally involve the use of a range of bulk nutrients (e.g., total organic carbon, total nitrogen) and stable isotopes [44,42,45–47], which face a trade-off between sensitivity, specificity and cost. While such drawbacks can be partially addressed with isotopic analyses on compounds with greater taxonomic resolution (e.g., lipids, amino acids), such methods are currently relatively costly and thus may not be practical for large-scale implementation as a verification strategy.

Comparatively, several aspects that make eDNA attractive as a potential blue carbon (and nitrogen) tracer and proxy [42,47–49] are its taxonomic specificity, sensitivity, as well as the relative ease of sample collection and processing and short analytical times, with relatively straightforward quantitative interpretation for quantitative polymerase chain reaction (qPCR). All these factors can reduce sampling and analytical costs. With sufficient validation, community-level data can offer comprehensive information about the taxa contributing to carbon sequestration in potential carbon sink locations, as well as ecological impacts of the mCDR intervention, and quantitative approaches such as qPCR can potentially serve as a useful indicator of the absolute contribution of blue carbon taxa of interest. While far from readiness as a primary tool at present, in conjunction with more established biogeochemical methods, eDNA can serve as powerful additional lines of evidence about the fate of blue carbon in marine systems, that can be difficult to obtain via other means.

In this case study, we developed and rigorously tested a species-specific quantitative PCR (qPCR) assay for *Saccharina latissima.* We used this assay (adapted for use as a digital PCR [dPCR] assay), coupled with metabarcoding-based community analyses to examine sediments underlying a ~ 1.25-hectare kelp farm in coastal Maine. *S. latissima* is the only species grown at this farm, which has been in existence since 2009, making it the longest continuously operating kelp aquaculture farm in the USA. Fine, anoxic muddy sediments similar to those common under this farm [44] are theoretically well-suited for preservation of organic matter given their physical favorability for promoting hypoxic subsurface conditions, which are known to be conducive for biomass preservation [50,51]. However, coastal nearshore sediments are also subject to more intense disturbance from biological (e.g., bioturbation) and physical sources (e.g., hydrodynamic disturbance) than deeper waters, which is why conventional wisdom suggests that long-term carbon sequestration in shallow benthic sediments is unlikely compared to deeper waters [31,52]. However, dredging and trawling is prohibited from leased areas for aquaculture in Maine, which keeps anthropogenic disturbance of the benthos to a theoretical minimum. In addition, based on our knowledge of prevailing currents at the site and estimated sinking rates for kelp biomass in the literature [53], the relatively shallow depth of the site allows for theoretically efficient conveyance of relatively fresh kelp biomass to the seabed, with minimal opportunity for degradation during transport. While models suggest that shed particulate biomass from kelp farms can theoretically travel great distances, such studies are usually conducted for waters much deeper than at our study site (~10 m) [37], In recent years, contrary to the prevailing paradigm about carbon sequestration in coastal sediments, there has been some evidence to suggest that sediments underlying kelp farms can accumulate significant amounts of organic carbon over the course of their operations [44], perhaps due to a combination of factors that minimize benthic disturbance and rapidly convey biomass to the seabed for burial. Furthermore, given the rise in kelp farming worldwide, there is value in empirical studies to examine the as-of-yet poorly understood fate of kelp biomass shed from kelp farms. While this study is not meant to be extrapolated to sediments in deeper waters, shallower waters can also serve as more tractable study sites for answering more general questions about the impact of macroalgal biomass enrichment on benthic communities, even if the intensity of such enrichment is likely to be lower than in biomass sinking mCDR interventions. Hence, this farm is intended to serve as a testbed for eDNA to assess 1) if a clear signal of kelp biomass can be detected in the underlying sediments, 2) if the carbon and nitrogen content in sediments is noticeably elevated and can be linked to the presence of kelp DNA, and 3) what, if any, effects there might be on the benthic microbial and eukaryotic communities due to kelp aquaculture.

## Materials and methods

### Field sampling

The study site is a ~ 1.25-hectare farm in Casco Bay close to Chebeague Island, Maine, USA. (Lease ID: CAS CHEB2; latitude, longitude: 43.72159, −70.14764). This farm is large by northeastern U.S. standards [54], but small compared to regions where kelp aquaculture is more established and other newer farm operations in the Pacific. The farm has been operating since 2009, with an annual growing season from seeding in roughly September-November, and harvest from April-May). The farm is situated in water of around 10 meters in depth (mean low water), and the seabed is muddy sediment. Mean sedimentation rates within the farm are around 4 mm/year [44]. A site map, with sampling site descriptions and coordinates can be found in S1 Fig A1, and S1 Table A1 in S1 File.

On 13 May 2021, just after harvest, sediment cores were collected at one site inside the center of the farm (n = 3 cores) and one site outside the farm (n = 3 cores). Cores outside the farm were collected at a similar depth (~9–10 m) upstream of prevailing tidal currents and subject to minimal influence from the farm itself, informed by 3 years of previous sampling of seawater chemistry at the site. Sediment cores were collected using a Haps corer with a 4.5-liter volume stainless steel sample tube (KC Denmark) mounted on the RV Ira C. of the Darling Marine Center (DMC), University of Maine. Sample tube dimensions are 315 mm long, with inner and outer diameters of 136 mm and 140 mm respectively.

On 1 June 2022, also post-harvest, the same sites (inside and outside of the farm) were resurveyed (n = 3 cores per site) using the same sampling methods as in 2021. An additional site outside the farm and two sites inside the farm were also sampled (n = 3 cores per site). All sites were additionally surveyed via a remotely operated vehicle (BlueROV2, Blue Robotics), and the only sites where kelp detritus was visually observed accumulating on the seabed were the two additional sites inside the farm added in 2022. These two additional sites were selected to represent portions of the seabed where kelp biomass was most likely to be accumulating within the farm.

At each sample location, sediment within the stainless-steel sample tube of the Haps corer was sub-cored using a single acid-washed polycarbonate tube (10 cm internal diameter, 30 cm length), taking care to maintain an undisturbed water column on the surface of each core. Core tubes were capped on both ends with acid-washed sleeve caps (SC-4 ½, Caplugs), placed on ice, and transported to the lab where they were placed in tanks of UV-sterilized filtered and aerated seawater in the dark at 12˚C prior to processing, which occurred within 48 hours of core collection.

### Lab processing

#### Core slicing.

All glassware and plasticware were acid-washed before use and between each sample. Metal scoopulas and other utensils were cleaned with 10% bleach instead.

Sediment cores were extruded and sliced into sections with thin plastic sheets and plastic ring segments. In 2021, 6 cores were sampled in total over two sites. Subsamples were taken at 0.5 cm depth intervals from 0–10 cm, and at 1 cm depth intervals afterwards up to the maximum depth of sediment in the core (ranging from 17–22 cm). These depth intervals were selected to cover the likely mixed layer of the sediment column and below into undisturbed sediment, with higher sampling resolution in the shallower depths which are most likely to correspond to the operational history of the farm [44]. In 2022, 15 cores were sampled in total over 5 sites. Subsamples were taken at 1 cm depth intervals down to a maximum depth of 10 cm instead, given that the highest kelp eDNA signals were observed in the first 5 cm of the cores in the 2021 samples. No distinguishable pieces of kelp, or other macrophytes, were observed in any of the sediment cores or subsamples.

### eDNA

The wet weight of each sediment core section was measured on an analytical balance (Sartorius 1212 MP; readability to 0.001 g), and sediment at the center of each slice was homogenized with a clean metal scoopula, before being subsampled for eDNA and frozen at −20°C prior to extraction. Wet weights of the eDNA subsamples were also measured.

In 2021, 8 depth horizons were analyzed for eDNA per core (0–0.5 cm, 0.5–1 cm,1–1.5 cm, 2–2.5 cm, 3–3.5 cm, 4–4.5 cm, 4.5–5 cm, and 6–6.5 cm). In 2022, 5 depth horizons were analyzed for eDNA per core (0–1 cm, 1–2 cm, 2–3 cm, 4–5 cm, and 6–7 cm).

For eDNA subsamples, approximately 0.25 g of sediment was extracted using the PowerSoil Pro kit (Qiagen), followed by additional clean-up with the Genomic DNA Clean & Concentrator kit (Zymo Research). DNA extracts were quantified with a Nanodrop ND-1000 spectrophotometer (Thermofisher Scientific) and then diluted to ~8 ng/µL for dPCR and amplicon sequencing. All samples were re-quantified with a Qubit 3.0 fluorometer and the Qubit dsDNA BR kit (ThermoFisher Scientific), and frozen at −80°C until use for dPCR and metabarcoding. Extraction blanks were conducted for each batch of extracted samples and considered to pass muster if no DNA was quantified via Qubit, and no COI amplification occurred during dPCR. Extraction blanks were not sequenced.

### Sediment bulk carbon and nitrogen analyses

Remaining sediments in each slice were weighed as above for wet weight, dried in a Precision 51221126, drying oven (Thermo Fisher Scientific) at 60°C for up to a week and weighed for dry weight. Dried samples were ground up either manually with a mortar and pestle (2021) or with the aid of a DC-5 soil crusher (Custom Laboratory Equipment Inc.) followed by finer manual grinding (2022). All of the 2021 subsamples (ranging from 27 to 32 subsamples per core, depending on the maximum height of the sediment column within each core, which was variable) were sent for total organic carbon (TOC), total carbon (TC), total nitrogen (TN), and ^210^Pb analysis as described in [44].

Samples from 2022 were analyzed by the Bigelow Analytical Services at the Bigelow Laboratory for Ocean Sciences on a Costech ECS 4010/Thermo DELTA V Advantage IRMS for total carbon, total nitrogen, ^13^C, and ^15^N. These metrics were chosen to maximize the data obtainable from each sample, given limited resources and the fact that acidified samples would lead to uncertain losses of nitrogen. In addition, sediments at our study site were expected to be low in carbonate, and thus we expected total carbon and total organic carbon values to be similar. To determine if this was so, a subset of the 2022 samples (n = 12) were acidified and run for total organic carbon to compare to the total C data in non-acidified samples. This subset was chosen to cover the range of total carbon values observed in the entire dataset. The TC to TOC ratio was 0.939 + /- 0.0117 and was judged to be sufficiently close to 1 for simplicity. Results of the TC to TOC regression can be found in S1 Fig C3 in S1 File.

### Development of a *Saccharina latissima* dPCR assay

A *S. latissima*-specific qPCR assay was developed targeting a 147 base pair fragment of the mitochondrial cytochrome oxidase 1 (COI) gene and adapted for use as a dPCR assay on the QIAcuity Digital PCR system (Qiagen). Primer and probe sequences are identical for both qPCR and dPCR variants of the assay (Table 1). An *in-silico* analysis of COI sequences available on Genbank for species closely related to *S. latissima* showed that those taxa had an average of 1–3 base pair mismatches for the forward primer, and 1–2 mismatches for the reverse primer. No other congeneric species are present in the Gulf of Maine, and other kelp species showed a minimum of 3 mismatches for both forward and reverse primers. Furthermore, we conducted *in-vitro* cross reactivity tests on genomic DNA extracts from a comprehensive list of common macroalgal species present in the Gulf of Maine (S1 Table in S1 File). Development of the qPCR assay proper is discussed in detail in S1 Section B in S1 File.

**Table 1:** Primer and probe sequences for the *S. latissima* COI qPCR assay.

Name	Sequence (5’ → 3’)	Length (bp)	Tm (°C)	% GC
**SacCOI_Fwd**	CCC CTC TTT AAT CTT GCT TC	20	59.5	45
**SacCOI_Rev**	CCT GAA AGA TGG AGA CTA AAT ATA G	25	59.7	36
**SacCOI_Probe**	AGC GTC CTC ATT GGA	15	69	50

Each dPCR reaction was prepared as follows: 3.4 µL of Qiacuity Probe Kit Master Mix (Qiagen), 0.544 µL of the probe, 1.088 µL each of both forward and reverse primers, 5.88 µL of nuclease-free water, and 4 µL of sample, for a total reaction volume of 16 µL. All samples were run on 8.5k partition QIAcuity nanoplates (both 24 and 96-well) and performed on a QIAcuity Four instrument (Qiagen).

The dPCR thermal cycler conditions were as follows: initial denaturation at 95°C, for 2 minutes, followed by 40 cycles: denaturation at 95°C for 15 seconds, and combined annealing and extension at 60°C for 1 minute.

### Kelp biomass spiking experiment

We conducted a laboratory-based biomass spiking experiment to determine the relationship between *S. latissima* COI gene copy number and known biomass in marine sediments. *S. latissima* blades were collected from thalli growing off the dock at the Bigelow Laboratory for Ocean Sciences in May 2023 and then lyophilized in a VirTis Advantage XL-70 (SP Scientific), and ground into a fine powder. Fresh, healthy portions of the blades were used for consistency. Kelp powder was added to sediment slurries made from a 50:50 mix of autoclaved filtered seawater and muddy sediment from a fully saline (32 parts per thousand) part of the Damariscotta River, Maine. *S. latissima* was known to be present on hard substrata, both natural and artificial in the surrounding area. Sediment was collected by divers at depths of 5–7 m, from anaerobic layers of intact sediment cores. Kelp powder was added to the sediment slurries at the following percentages by wet weight (0, 0.01, 0.05, 0.10, 0.50, 1.00, 2.00, and 5.00%), in 50 mL conical tubes (n = 3 replicates per added biomass level). All weighing was conducted on an analytical balance (Denver Instrument APX-200; readability to 0.0001 g)

Sediments and kelp biomass were vigorously mixed with a Digital Vortex Mixer (Fisher Scientific, Model 945415) at maximum speed (3000 rpm), until no kelp particles were visually distinguishable from the slurry, for about 30 seconds. Slurries were immediately subsampled for eDNA analysis, using the extraction protocols described above in the eDNA section of the kelp farm survey. Added wet kelp biomass was converted to added dry kelp carbon, using the mean wet weight to dry weight ratios of the added kelp (~12.5%) and carbon content of the resultant lyophilized kelp powder (~33%). This yielded kelp dry total C additions of 0% (no addition control), 0.00004%, 0.002%, 0.004%, 0.02%, 0.04%, 0.08%, and 0.21% in the kelp spiking experiment, by mass of sediment slurry. In addition, an additional set of controls were prepared using sediment from Great Salt Bay, a less saline part of the estuary (44.065248N, 69.517443W) at the head of the Damariscotta River, where kelp does not naturally grow.

### 16S and 18S amplicon sequencing

To assess the benthic prokaryotic and eukaryotic communities inside and outside the kelp farm, we subjected the DNA extracts from the sediment cores to 16S (V4-V5 region) and 18S (V4 region) amplicon sequencing (i.e., metabarcoding). The same DNA extracts used for the COI assay were used for amplicon sequencing. For 16S, DNA extracts (~1–10 ng/µl) were sent to the University of New Hampshire (UNH) Hubbard Center for Genomics Studies (HCGS) in 2021 for amplicon sequencing on the Novaseq 6000 platform (Illumina), following Earth Microbiome Project protocols [55]. In 2022, 16S DNA extracts were sequenced at Dalhousie University’s Integrated Microbiome Resource (IMR), where 16S gene amplification, barcoding, and sequencing was on a MiSeq platform (Illumina) following IMR protocols. To assess microeukaryote and metazoan presence and diversity, the V4 region of the 18S ribosomal RNA gene was sequenced. 18S subsamples in both 2021 and 2022 were sequenced at IMR, similar to as described above.

### Data analysis

All statistics were conducted in RStudio (version 2024.12.1) [56]. All underlying code and data used for the analyses and plots in this study, as well as statistical analyses output, can be found on Zenodo at https://doi.org/10.5281/zenodo.15133761. All raw 16S and 18S sequences used in this study have been deposited with the National Center for Biotechnology Information (NCBI) under BioProject ID PRJNA1241800.

### Kelp biomass spiking experiment

For all sediment samples (i.e., kelp biomass spiking experiment and field samples), mean COI gene copy number per gram of dry sediment (mean COI) was calculated from the dPCR data using the equation in S1 Fig C1 in S1 File.

For the kelp biomass spiking experiment, an ordinary least squares (OLS) regression was fitted to added dry kelp carbon (as a percentage of mass of dry sediment) against mean COI gene copy number per gram of dry sediment using the lm() function, with the y-intercept coerced to zero. To confirm that our models fulfilled OLS assumptions, standard diagnostic plots (for linearity of relationship between the variables, homoscedasticity, normality of residuals) and Shapiro-Wilk’s test (for normality of data) were used.

### Kelp farm data: COI and nutrients

Ordinary least squares (OLS) regressions were fitted to the eDNA (i.e., *S. latissima* COI gene copy number per gram of dry sediment) and sediment nutrient (i.e., carbon and nitrogen) data from the kelp farm. COI and nutrient data were log transformed to allow models to fulfill OLS assumptions.

OLS effect sizes are presented as beta and standard beta values. Datasets from 2021 and 2022 were treated independently, given the different sampling resolutions and maximum sampling depths. Given that the 2022 dataset had multiple sites for each site type, site number was nested within site type. For the nutrient data, acidified (i.e., TOC% and TN% [acidified]) and non-acidified (i.e., TC% and TN%) datasets were run separately.

To account for depth-related differences in COI gene copy numbers and nutrients that may confound potential site-related effects between cores, these data were also integrated into a mean value per 1 cm^3^ of sediment, based on the maximum sampled depth in the core and the volume of sediment sampled for each depth horizon, with extrapolation between depth horizons that were not sampled. The inverse of the gene copy number to kelp carbon equation from the kelp spiking experiment was also used to estimate the amount of kelp carbon per 1 cm^3^ of sediment in each core (Kelp carbon = (7.093*10^-11^) *COI gene copy number), assuming that the kelp carbon to COI gene copy number relationship from our spiking experiment also holds true for the kelp eDNA in our sediments; however see the Discussion for additional details on how kelp degradation state may impact these results.

For the 2021 dataset, despite having nutrient data for up to a mean depth of 20 cm, we focused on the depths sampled for eDNA (i.e., 0–6.5 cm). These shallower depths (<10 cm) were chosen, as they were more likely to correspond to recent sediment deposition within the operational life of the farm (~15 years; per the estimated sedimentation rates of ~4 mm/year from the ^210^Pb profiles in [44]).

For integrated kelp carbon and nutrient estimates, which normalized for depth-related differences between cores, unequal variance between samples from inside and outside the farm violated OLS assumptions, even after log transformation of the data. Henceforth, these datasets were analyzed with non-parametric Kruskal-Wallis tests and post-hoc Dunn’s tests (with Benjamin-Hochberg correction).

### Kelp farm data: Metabarcoding

For the 16S and 18S metabarcoding data, forward and reverse reads were assembled, filtered, and trimmed using a custom dada2 [57] pipeline implemented on the Bigelow Laboratory’s supercomputing cluster. Given the differences in sequencing methods between the 16S samples in 2021 and 2022, all four datasets (both genes, for both years) were analyzed separately. The SILVA NR99 v138.1 database was used to assign taxonomy for the 16S dataset, and the PR2 v 4.13.0 database was used for the 18S dataset. R packages used for downstream analyses were dada2, phyloseq [58], vegan v6.1, [59] and MicrobiomeStat v1.2 [60]. Phyloseq objects were assembled using the pipeline output and sample metadata. 16S reads for chloroplasts and mitochondria were removed. The 2021 16S and 18S samples, and the 2022 16S samples were rarefied to the minimum read count in the respective datasets to retain all samples (2021 16S: 24500; 18S: 1300; 2022 16S: 3100). The 2022 18S samples were rarefied to 1100 reads to only remove 2 samples in the dataset, out of 75.

Alpha diversity metrics (observed diversity & the Shannon-Wiener diversity index) were generated and tested for normality with Shapiro-Wilk tests. The former only accounts for numbers of taxa, while the latter also accounts for abundance of each taxon. Given that none of the diversity metrics showed normal distributions, henceforth, Kruskal-Wallis tests were used to assess if there were any statistically significant differences in diversity metrics. Any significant Kruskal-Wallis results were assessed with post-hoc Dunn’s tests.

To assess beta diversity, stacked barplots were made with the MicrobiomeStat package, with the top 30 taxa selected, and the remaining taxa aggregated into an “other” category. Amplicon sequence variants (ASVs) were aggregated to the family level for the 16S datasets, and the genus level for the 18S dataset, to strike a balance between taxonomic resolution and minimal unassigned taxonomic identities. Principal coordinates analysis (PCoA) plots were then made based on Bray-Curtis distances to examine similarity between samples.

Permutational ANOVAs (PERMANOVAs) were conducted with the adonis2 function in the vegan package, to examine the statistical significance of the various covariates (e.g., *S. latissima* COI gene copies, nutrient data) on the community data, using Bray-Curtis distances. PERMANOVAs were conducted for both the full datasets, which lacked complete nutrient data, and a subset of samples that had complete nutrient data. PERMANOVA effect sizes were calculated as partial Omega-squared values (ω2) using the MicEco package [61].

To identify potential indicator taxa, volcano plots were made for taxa with the largest log-fold differences between samples taken from sites inside and outside the kelp farm. This was also done using the MicrobiomeStat package. Volcano plots were chosen over other indicator taxa identification methods such as DESeq and SIMPER because of greater interpretability and a greater list of statistically significant potential candidate taxa. However, the volcano plots were only able to identify indicator taxa associated with site type and not *S. latissima* COI gene copies. Hence, Spearman correlation tests were also conducted in the phylosmith package [62] to examine taxa significantly enriched for each site type that showed statistically significant correlations with *S. latissima* COI gene copy number values, assumed to be by extension to be correlated to kelp biomass presence in sediments.

## Results

### *S. latissima* assay standard curve performance, sensitivity and specificity

*In-vitro* cross reactivity testing of the assay against common macroalgal species present in the Gulf of Maine showed a lack of cross-reactivity, consistent with the in-silico analysis (S1 Table B2 in S1 File). In initial trials using traditional qPCR, the limit of detection (LOD) and limit of quantification (LOQ) was constrained down to at most 5 COI gene copies per µL of qPCR reaction and may be lower. When tested against a dilution series of standards spanning 5 orders of magnitude from 2.73 to 273 thousand gene copies per µL, there was a strong, significant positive relationship between the levels tested (y = −3.197x + 37.049, p < 0.0001, R^2^ = 0.999) (S1 Fig B2 in S1 File) and the qPCR cycle threshold (Cq) values. The theoretical limit of the dPCR assay is one gene copy per reaction mix, and because dPCR is based on an absolute quantification method, it does not require standards [63]. Direct comparisons between the qPCR and dPCR assays showed a consistent relationship between qPCR and dPCR values across all treatment levels (S1 Fig B3 in S1 File), with the former method generally giving slightly higher values than the latter (by 1.633 + /- 0.508 times), probably because of the absolute quantification capabilities of dPCR.

### Application of the *S. latissima* dPCR assay on natural sediments spiked with kelp biomass

**Fig 1:**
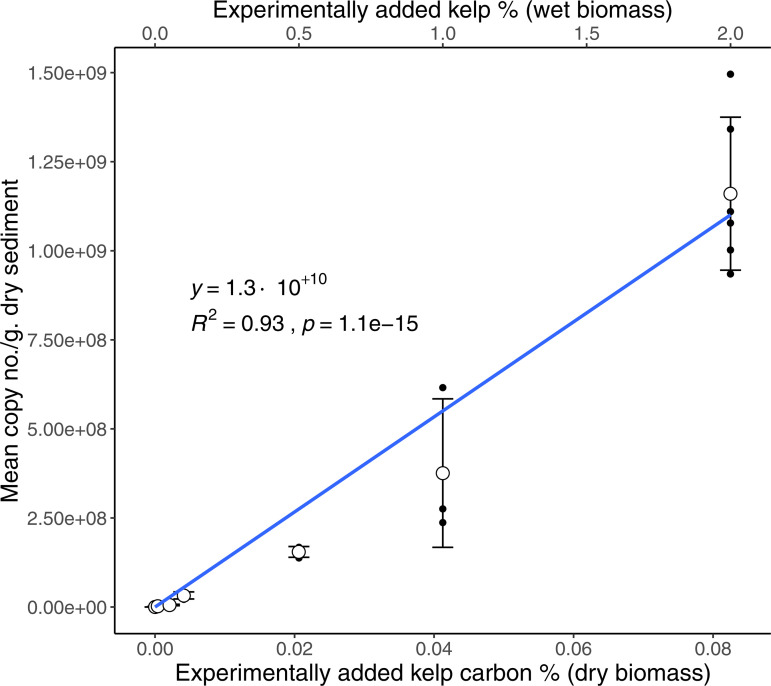
Mean COI gene copy number per gram of dry sediment, against experimentally added dry kelp carbon percentage of *S. latissima* tissue. The blue line and associated output represent the OLS regression fitted to the dataset, with the gray borders as the 95% confidence interval. The black bars represent standard error at each treatment level, with the white unfilled circles as the mean. An alternative x-axis on the top of the plot corresponds to the percentage of wet biomass added to each treatment.

Overall, the kelp spiking experiment showed a strong linear correlation between *S. latissima* COI gene copy number and added biomass in a range from approximately 0.0004% to 0.08% added kelp carbon by dry weight (F(1, 19) = 250.8, p < 0.0001, adjusted R^2^ = 0.926, y = 1.3 x 10^10^) (Fig 1). The greater variance between replicate samples at higher treatment levels (0.04% and 0.08%) may be due to clumping of kelp biomass at these larger additions despite vigorous homogenization. It is worth noting that at 0.2% dry kelp carbon addition (not shown in Fig 1, but tabulated in S1 Table C1 in S1 File), mean COI values were similar to that of the 0.08% level, and thus lower than the expected mean COI values based on the linear regression between the levels presented in Fig 1. A table of values and standard deviation, including values of dry kelp carbon and wet kelp biomass at each treatment level can be found in S1 Table C1 in S1 File.

### Kelp farm study

#### Sediment total carbon and nitrogen.

TC%, TOC%, TN%, and TN% (acidified) were measured in 2021, and TC% and TN% in 2022. d^13^C and d^15^N were measured in both years for the above metrics. In addition, an integrated value was calculated for each non-isotopic metric based on the mean value for all sampled depths, normalized to a volume of 1 cm^3^ of sediment.

Briefly, there was no clear difference in sediment carbon and nitrogen content between sites inside and outside the farm in both years of the study. Full details of the nutrient analyses for both years can be found in S1 Section D S1 File, including results of other factors omitted for brevity (e.g., depth). The only nutrient metrics that showed a statistically significant effect of site type were the integrated TN% value in 2021 and TN% in 2022. Neither TC% nor TOC% showed a significant difference based on site type in both years. d^13^C and d^15^N showed no site type-related differences in both years, for both acidified and non-acidified datasets.

### *S. latissima* COI gene copy numbers associated with the Chebeague Island kelp farm sediments

Sediment cores collected in 2021 were analyzed for *S. latissima* eDNA abundance using the species-specific COI dPCR assay, with a depth profile from 0 to 6.5 cm (S1 Fig C4, S1 Table C2 in S1 File). An OLS regression conducted on the 2021 dataset was non-significant (F(2, 45) = 2.43, p = 0.100, adj. R^2^ = 0.06).

Total gene copy numbers within cores from 2021 were summed down to the maximum sampled depth and converted to mass of dry kelp carbon per 1 cm^3^ volume of sediment, using the equation from the kelp spiking experiment (S1 Fig C5, S1 Table C3 in S1 File). For this integrated, derived kelp carbon value, a Kruskal-Wallis test for kelp carbon against site type was non-significant (Kruskal-Wallis chi-squared (KW χ^2^) = 0.429, df = 1, p = 0.513)

**Fig 2:**
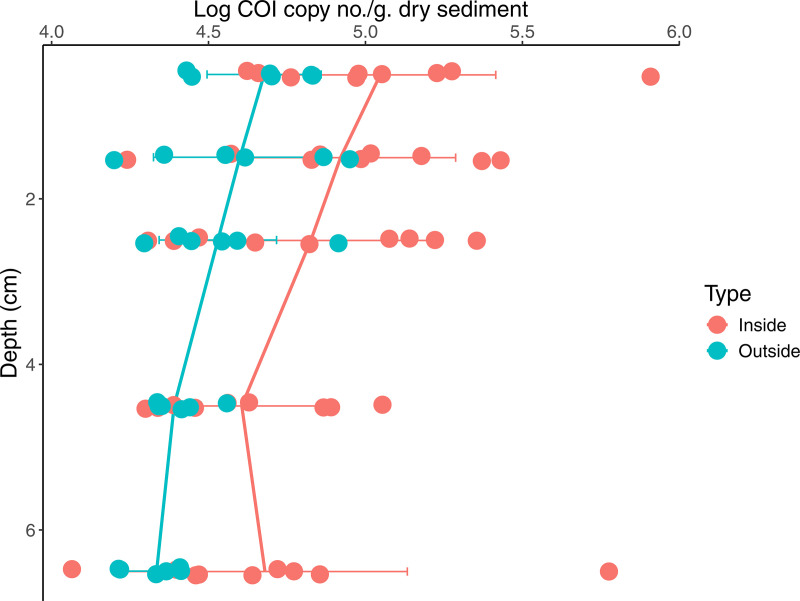
Log *S. latissima* COI gene copy number by depth for the 2022 samples. The colored lines represent mean values at each depth sampled, colored by site type. The horizontal bars with whiskers represent standard deviation at each depth. Depth values presented correspond to the middle of each depth horizon (e.g., 6.5 cm corresponds to the 6−7 cm depth horizon). There is a statistically significant difference in log *S. latissima* COI gene copy numbers between samples taken from inside and outside the farm (p = 0.036, beta = −0.20, std. beta = −0.56).

Based on the 2021 results where higher resolution (~0.5 cm) sampling of surficial sediments (0−3 cm) was deemed to be unnecessary, sediment cores collected in 2022 were instead analyzed with a depth profile from 0 to 7 cm, with 1 cm intervals for the first 3 cm (Fig 2, S1 Table C4 in S1 File). An OLS regression conducted on the 2022 dataset was significant (F(5, 69) = 14.51, p < 0.001, adj. R^2^ = 0.48). Within the model, site type was statistically significant (p = 0.036, beta = −0.20, std. beta = −0.56; using type = inside as the baseline). Log COI values were greater in samples from inside the farm by a mean value of 0.310 (inside = 4.816; outside = 4.506), after aggregating across all depths. Relatively-speaking, mean COI gene copy numbers were approximately double in sediments inside the farm compared to outside the farm. Sample depth was statistically significant and negative (p < 0.001, beta = −0.06, std. beta = −0.38; using depth = 0 cm as the baseline). The only site type: site number pair that was statistically significant is Site 2 (Inside) (p < 0.001, beta = 0.43, std. beta = 1.19), with greater mean log COI values than the other sites (ranging from 0.423 to 0.628, i.e., ~ 2.7 to ~4.4x greater in mean COI values).

**Fig 3:**
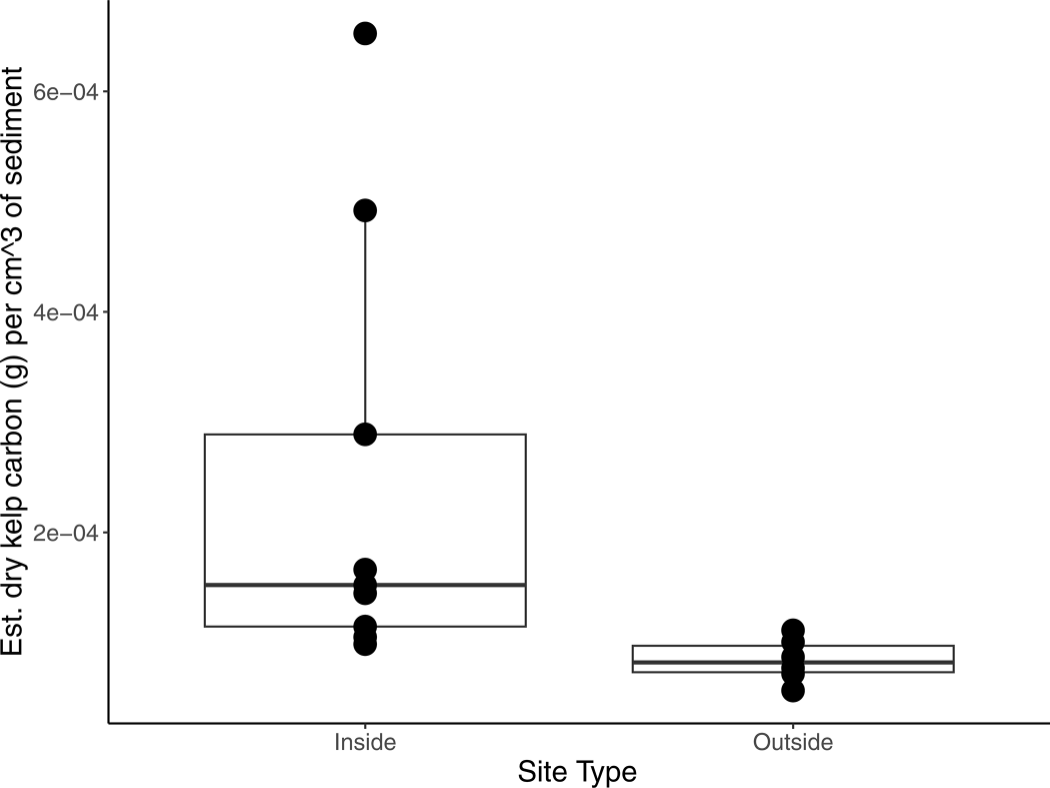
Box plot of derived grams of *S. latissima* carbon in the 2022 cores per 1 cm^3^ volume of sediment, integrated down to the maximum depth sampled for eDNA (7 cm). A Kruskal-Wallis test for derived kelp carbon against site type was significant (KW χ^2^ = 8, df = 1, p = 0.00468).

Similarly to the 2021 dataset, an integrated kelp carbon value (henceforth referred to as the “derived kelp carbon value”) was also derived from the linear relationship established in the kelp biomass spiking experiment (Fig 3, S1 Table C5 in S1 File). A Kruskal-Wallis test for kelp carbon against site type was significant (Kruskal-Wallis chi-squared (KW χ^2^) = 8, df = 1, p = 0.00468). The post-hoc Dunn’s test showed sediments from inside the farm to have a greater derived kelp carbon value than outside of the farm (p = 0.0023), by a mean value of ~2.9X.

To summarize, overall, the 2021 dataset, with much less sampling effort, shows no statistically significant difference in *S. latissima* COI gene copies and derived kelp carbon between inside and outside the farm. In contrast, the 2022 dataset showed a statistically significant effect of site type on both metrics. Based on the box plots of derived kelp carbon values (and by extension, COI gene copies), and the statistical significance of Site 2 in the OLS, it is clear that there are sites with markedly greater kelp eDNA presence within the farm.

### 2022: 16S + 18S metabarcoding data

A variety of community-level diversity analyses from the 2021 sampling did not show any statistical differences between cores taken from inside and outside the farm. This, in part, led to a more robust sampling effort in 2022 to better understand the level of variation within the farm sediments, and focus on a subset of depths that the 2021 sampling indicated were more likely to show significant differences in kelp eDNA presence (i.e., shallower depths) that could, in turn, be linked to differences in the sediment community as revealed by 16S and 18S metabarcoding. The full results of the alpha diversity and beta diversity analyses conducted on the 2021 16S and 18S datasets can be found in S1 Section E in S1 File.

### Alpha diversity metrics: 16S and 18S

Briefly, both the 16S and 18S datasets in 2022 showed modest but statistically significant differences in the Shannon index, with the 16S dataset also being significant for observed diversity. Alpha diversity was greater in sediments outside the farm, compared to inside the farm. Depth and *S. latissima* COI were non-significant. The full results of the alpha diversity analysis on the 2022 datasets can be found in S1 Section F in S1 File.

### Beta diversity: 16S

Given the statistically significant differences in alpha diversity with site type, beta diversity analyses were conducted to examine subtler variations in taxonomic distribution with site type, and other covariates of interest (e.g., *S. latissima* COI gene copy number) that could be reflective of kelp biomass presence.

**Fig 4:**
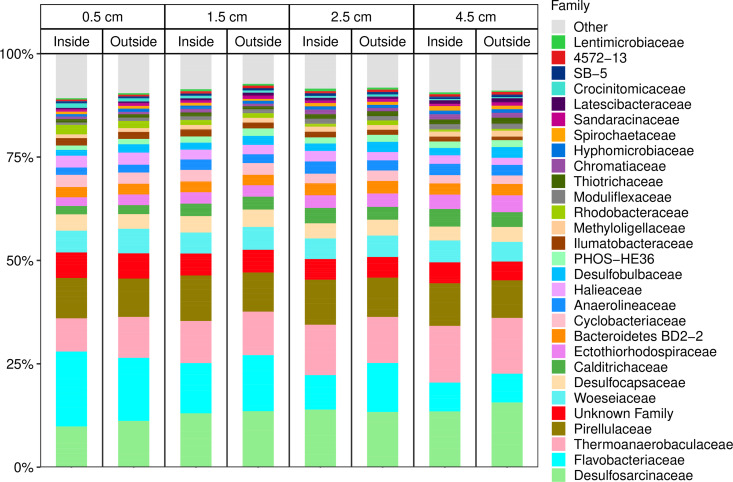
Stacked bar plots of the top 30 microbial families in the 2022 16S dataset by relative abundance. Bars are grouped together by sample depth (with the value representing the mid-depth of each depth horizon), and then by site type. Remaining taxa have been aggregated into an “Other” category.

Firstly, family-level barplots of the top 30 microbial taxa were made, grouped by depth and then site type (Fig 4). As is apparent in the figure, there were no substantial differences in these major taxa, either with depth or site, inside versus outside the farm. Major taxa included lineages belonging to anaerobic clades including sulfate-reducing bacteria, *Desulfosarcinae* and *Desulfocapsaceae;* anaerobic fermentative bacteria, *Thermoanaerobaculaeceae* and *Anaerolinaceae*, as well as more facultative groups, *Flavobacteriaceae* and *Pirellulaceae.* These taxa are consistent with other marine sediments where sulfate-reduction is the primary form of anaerobic respiration, while fermentation and aerobic respiration, resulting from bioturbation driven oxygen inputs, can facilitate the breakdown of complex organic matter [64].

**Fig 5:**
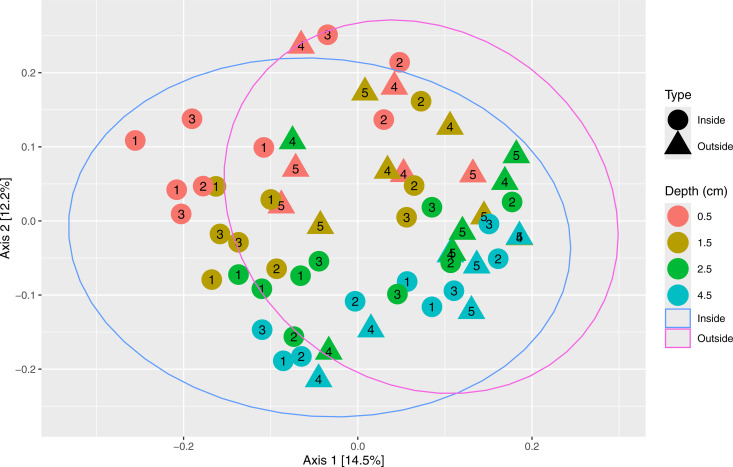
Principal coordinates analysis (PCoA) plot of the 2022 16S microbiome dataset, based on Bray-Curtis distances. Each point represents a single sample, with shape representing site type (inside vs outside the farm) and color representing the depth of the sample. Site number (1 to 5) is overlaid on top of each point. Ellipses have been drawn around points of the two site types and are also colored by type. An alternative of this plot with color representing log COI gene copy number can be found as S1 Fig F7 in S1 File.

A Principal Coordinates Analysis (PCoA) of the 16S dataset (Fig 5) was used to examine dissimilarity in community composition between the different samples, based on Bray-Curtis dissimilarity, which takes into account both presence/absence and abundance. Once again, some depth-related clustering was apparent. In general, based on 95% confidence intervals, samples from inside the farm occupied a larger segment of PCoA space than samples from outside the farm, with the latter largely being a subset of the former. Nevertheless, points from inside the farm beyond the PCoA space occupied by outside samples were spread out across all 3 inside sites and all depths, rather than specific site numbers and/or depths. Likewise, samples with the highest *S. latissima* COI gene copy numbers did not appear to be clearly separated from samples from similar depth horizons, regardless of site type (S1 Fig F7 in S1 File).

PERMANOVA analyses were then conducted to examine factors of interest (i.e., site type, depth, COI, nutrients) that might show statistically significant associations with the Bray-Curtis distances between samples. For the full 2022 dataset, site type (p = 0.0014, ω2 = 0.0241) and sample depth (p = 0.0001, ω2 = 0.102) were statistically significant in structuring the microbial communities in the samples, while *S. latissima* COI gene copy number (p = 0.455) was non-significant.

For a subset of the 2022 dataset with nutrient data (0.5, 1.5, 2.5 cm), site type (p = 0.0036, ω2 = 0.0241), sample depth (p = 0.0001, ω2 = 0.0711) and TC% (p = 0.0077, ω2 = 0.0196) were statistically significant, while COI gene copy number (p = 0.282) and TN% (0.574) were non-significant.

Based on the PERMANOVA effect sizes, sample depth is more important in structuring 16S community composition than site type and TC%. This can also be seen in the PCoA, where there is more apparent visual separation based on sample depth, compared to site type.

**Fig 6:**
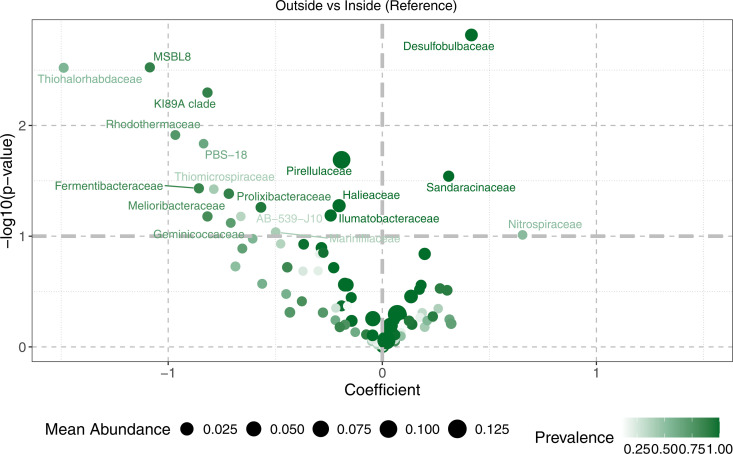
Volcano plot of potential microbial indicator taxa at the family level distinguishing samples from inside and outside the farm in the 2022 16S dataset. Potential indicator taxa are determined by differential log-fold changes, with a negative coefficient on the x-axis representing a relative enrichment in samples from inside the farm, and vice versa. The size of the dot for each taxon represents its mean abundance in samples across the dataset where present, while the color intensity represents its prevalence across all samples in the dataset. The dotted gray line on the y-axis represents a cut-off p-value of 0.1 for statistically significant taxa. A more stringent p-value cut-off of 0.05 would be represented by a y-axis value of ~1.3. Likewise, a p-value cut-off of 0.01 would be presented by a y-axis value of 2.

Given the statistical significance of site type in the PERMANOVA, a family-level volcano plot (Fig 6) was made to examine potential indicator taxa that may be associated with sediment inside and outside the farm. Using a p-value threshold of 0.05 (a log value of ~1.3) as the cutoff, taxa relatively enriched in sediments from inside the farm include *Pirellulaceae*, MSBL8, the KI89A clade, PBS-18, *Rhodothermaceae* and *Thiohalorhabdaceae. Desulfobulbaceae* and *Sandarinaceae* were comparatively more enriched in sediments from outside the farm.

To examine potential indicator taxa in further depth, a correlation analysis (S1 Table F3 in S1 File) was conducted to examine taxa that showed statistically significant correlations with *S. latissima* COI gene copy number, and by extension, possibly kelp biomass presence and quantity. Prominent taxa associated with sediments from inside the farm that were positively correlated with COI values include *Rhodothermaceae, Rubritaleaceae, Flavobacteriaceae, Prolixibacteraceae, Nitrosomonadaceae, Nitrincolaceae* and *Rubinisphaeraceae*. The only taxon in common between the correlation analysis and the volcano plot was *Rhodothermaceae*.

A list of the top 30 significant taxa, and their associated p-values, Spearman’s rho values and site types can be found in S1 Table F3 in S1 File. Spearman’s rho values range from −1 to +1, indicating negative to positive correlation respectively.

### Beta diversity: 18S

**Fig 7:**
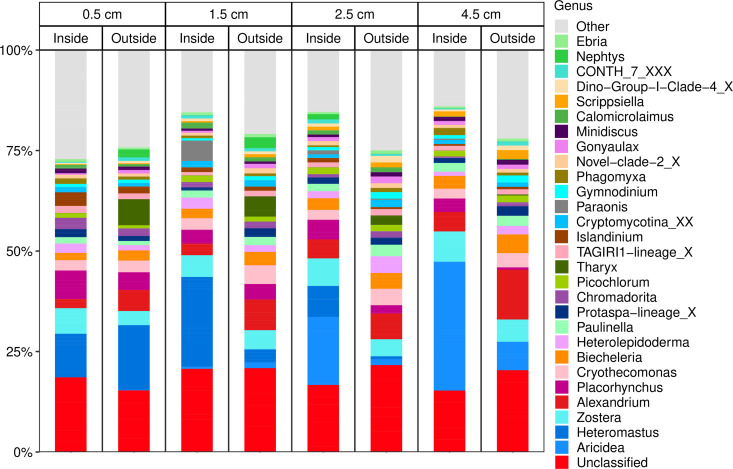
Stacked bar plots of the top 30 eukaryotic genera in the 2022 18S dataset by relative abundance. Bars are grouped together by sample depth (with the value representing the mid-depth of each depth horizon), and then by site type. Remaining taxa have been aggregated into an “Other” category.

The 2022 18S dataset showed more heterogeneity in taxonomic distribution of 18S ASVs between samples of different site types and depths (Fig 7), especially compared to the 16S dataset. Because of this taxonomic heterogeneity, no consistent depth-related and site-related trends in beta diversity were visually evident, although it must be noted that the Shannon Index (but not observed diversity) showed a statistically significant difference between the site types. From the barplots, the polychaetes, *Heteromastus,* and *Aricidea* appear to be more prevalent in samples from inside the farm, with the former being more dominant in the upper sediment layers, and the latter in the deeper sediment layers.

The main genera in the core microeukaryotic community include *Biechelaria, Alexandrium*, a member of the family *Thoracosphaeraceae, Cryothecomonas, Islandinium, Heterolepidoderma, Chromadorita,* and *Picochlorum*. The common eelgrass, *Zostera marina*, made up a small (~5%) but prominent proportion of ASVs, and was the only common macrophyte in the dataset, whether embryophyte (i.e., land plants) or macroalgae. By extension, it is also the only blue carbon contributor in the dataset. *S. latissima* reads were absent in the dataset, and other *Phaeophyceae* (e.g., *Fucus* spp., *Ascophyllum* sp.) were either also absent, or present in low single digit read counts in some samples.

**Fig 8:**
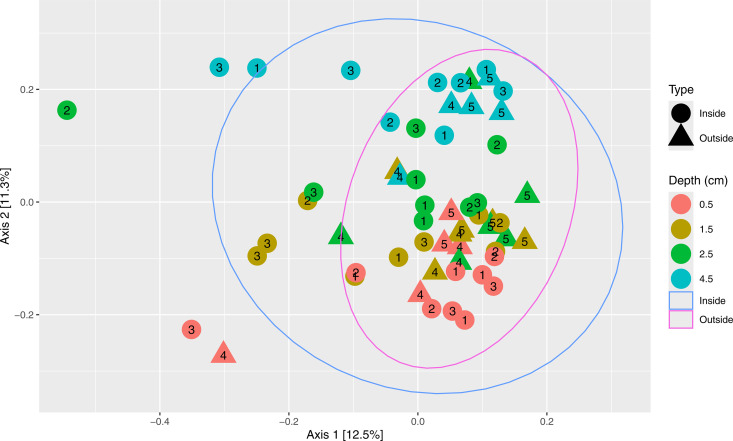
Principal coordinates analysis (PCoA) plot of the 2022 18S eukaryotic dataset, based on Bray-Curtis distances. Refer to Fig 5 for details on figure interpretation. An alternative of this plot with color representing log COI gene copy number can be found as S1 Fig F8 in S1 File.

The 18S PCoA analysis (Fig 8) showed stronger visual separation of samples by depth, with no obvious separation by site type, or COI gene copy number (S1 Fig F8 in S1 File) The ellipse drawn around samples from inside the farm (representing the 95% confidence interval) encompassed a greater amount of PCoA space, with samples from outside the farm largely being nested within this space. In contrast to other datasets, there were more relative outliers in PCoA space, with the majority consisting of samples from inside the farm, from various depths.

PERMANOVA analyses were then conducted to examine factors of interest (i.e., site type, depth, COI, nutrients) that might show statistically significant associations with the Bray-Curtis distances between samples. For the full 2022 dataset, site type (p = 0.0002, ω2 = 0.0401) and sample depth (p = 0.0001, ω2 = 0.123) were statistically significant in structuring the eukaryotic communities in the samples, while *S. latissima* COI gene copy number (p = 0.240) was non-significant. For a subset of the 2022 dataset with nutrient data (0.5, 1.5, 2.5 cm), site type (p = 0.0014, ω2 = 0.0373), sample depth (p = 0.0002, ω2 = 0.0600) were statistically significant, while COI gene copy number (p = 00.117), TC% (p = 0.311) and TN% (0.141) were non-significant. In general, the PERMANOVAs conducted on the 2022 18S dataset showed both site type and depth to be statistically significant, similar to the 2022 16S dataset (barring TC% being significant in the latter).

**Fig 9:**
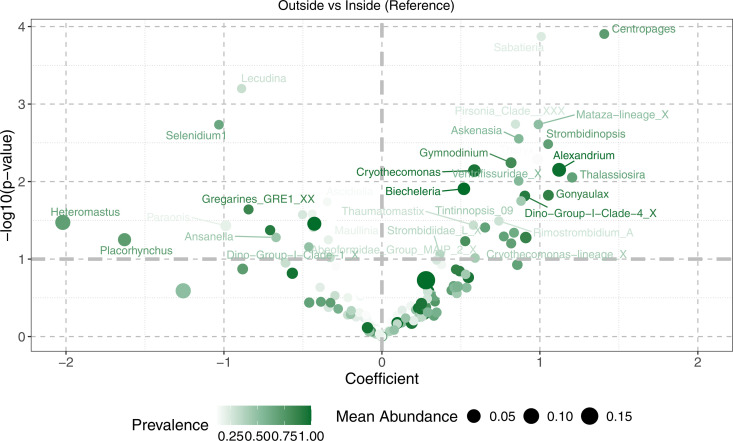
Volcano plot of potential eukaryotic indicator taxa at the genus level distinguishing samples from inside and outside the farm in the 2022 18S dataset. Refer to Fig 6 for details on figure interpretation.

Given that the majority of eukaryotic taxa could be resolved to the genus level, a genus-level volcano plot (Fig 9) was made to examine potential indicator taxa that may be associated with sediment from inside versus outside the farm. There were a relatively large number of potentially significant indicator taxa. Taxa such as *Heteromastus, Zostera, Polyplicarium, Gregarines* GRE1, and *Seledinium* 1 were comparatively more prevalent in samples from inside the farm, while many taxa that were comparatively enriched outside the farm include *Centropages, Strombidinopsis, Alexandrium, Gymnodinium, Cryothecomonas, Biecherlaria*, Dinoflagellate Group I Clade 4, and *Gonyaulax*.

The correlation analysis conducted on COI gene copy number (S1 Table F4 in S1 File) showed taxa such as *Gastrotricha, Pycnophyes, Oxystomina, Thalassiosira, Difficilina, Halomonhystera,* and *Parastrombidinopsis* to be positively correlated with COI gene copies inside the farm, with no clear overlap with the taxa identified in the volcano plot.

## Discussion

### Development of the *S. latissima*-specific dPCR assay and quantification of kelp eDNA in sediments

#### Assay development and performance.

The COI-based assay for *S. latissima* worked well in amplifying target DNA extracted from marine sediments. The primers were species-specific for *S. latissima* and did not show cross-reactivity with other related algal species that would be expected to be found in the Gulf of Maine. Further cross-reactivity testing is recommended should this assay be implemented in regions where other *Saccharina* species, or more prudently, other *Laminariales* are present.

In sediments spiked with known amounts of kelp biomass, there was a strong linear relationship between *S. latissima* gene copy number and kelp biomass. This indicates the potential to utilize the assay to not only detect kelp DNA in sediment; but also opens the possibility for it to be used as a quantitative tool to estimate the amount of species-specific carbon in marine sediments. When applied to field samples from the kelp farm study site, the COI assay consistently detected kelp eDNA at gene copy numbers above the LOD from both inside and outside the farm. However, the highest of these values were much lower compared to the tested dynamic range of the assay on the spiking experiment samples. Field samples ranged from single digit to low double digit gene copy numbers per µL of samples in each dPCR reaction, compared to the lowest tested levels in our biomass spiking experiment (~50 copies/µL in the 0.01% wet biomass/0.0004% dry kelp carbon treatment). This indicates that kelp biomass presence in the sediments in our study area is likely to be low even in samples where kelp eDNA shows the greatest relative abundance.

It is important to note that the relationship that we established between kelp biomass and COI gene copy number is for fresh, finely ground biomass, and is thus likely to represent a lower limit to this relationship. In addition, the lyophilized nature of our added biomass is likely to lead to rapid cellular lysis on introduction to sediments, leading to liberation of large amounts of eDNA. Natural kelp biomass in marine sediments is likely to be in various stages of degradation. Given that eDNA represents among the most labile fractions of biomass and is easily degraded by microbial activity [65], the ratio of eDNA to kelp carbon is likely to decrease over time as biomass degrades. Hence, recoverable kelp eDNA in natural sediments is theoretically an underestimate of actual remaining kelp carbon. By applying the results of the spiking experiment to field samples of a similar sediment type, we aim to estimate what the minimum value of kelp carbon in our sediments could be based on eDNA values. Further research is needed to determine how the relationship between kelp eDNA and organic carbon changes over time throughout the degradation process. In addition, it may be necessary to recalibrate the relationship in significantly different sediment types than analyzed here.

As for similar approaches in the literature, *Saccharina latissima* qPCR assays for the COI gene have already been developed for use in detecting the presence of the species in other settings (e.g., kelp in copepod gut contents [66]; zoospores in water [67]), but to the best of our knowledge, this is the first time such quantitative assays have been shown to work well on sediments [49,68].Other eDNA approaches to blue carbon have generally elected to use qPCR primers targeting a broader range of taxa (e.g., kelp in general [69,70]), or choose to use metabarcoding instead [25,48,71,72], estimating relative contributions to the carbon pool based on relative read counts (with varying degrees of validation to attempt to link eDNA data to sediment nutrient metrics).

Overall, our *S. latissima* assay is effective both as a qPCR assay (and thus likely to be more accessible to most labs) and a dPCR assay. While our study is not the first to develop a COI qPCR assay for *S. latissima*, our assay has been tested to be highly specific to the species in our study system and has been proven to be effective in marine sediments. While the cost of instruments and consumables for dPCR assays are higher than for qPCR assays, dPCR has the significant advantage of not requiring standards to be run each time a sample set is analyzed. This reduces personnel cost in terms of time to set up standards, cost of consumables for running standards, as well as overcoming issues related to degradation of standards over time or contamination of standards during multiple uses, making this approach a more cost-effective one for MMRV applications.

#### Use of dPCR for assessing kelp eDNA in sediments.

Another primary goal of this work was to assess the degree to which eDNA could be used to estimate how much kelp-derived carbon may be deposited in sediment. For this exercise, we aggregated all the COI gene copies present in a unit volume of sediment for each sediment core and used that to derive an estimate of total kelp carbon. The resultant values are a small percentage of TOC% and TC% values from these same cores, with the highest derived kelp carbon values being approximately a thousand-fold less (~0.0013%) than the observed range of TOC% (2.5–3.5%) and TC% values (3.25–3.6%) (S1 Table C1 in S1 File). Within the literature, a study [70] that investigated eDNA gene copies of various common macrophyte taxa in deep offshore sediments did not find a correlation with sediment bulk organic carbon. On the other hand, two studies [69,73] were able to find a correlation between *Zostera marina* eDNA and TOC%, as well as the C:N ratio, but not for other taxa, including macroalgae. However, a later study by the same team [70] was unable to determine any clear relationship between offshore sediment nutrient content and eDNA from various macrophyte taxa; and suggested that their findings may have been due to the relatively small contribution of such taxa to the bulk OC pool.

However, our derived kelp carbon values need to be treated with caution as eDNA is ultimately but a proxy of bulk biomass, and not a direct measurement of it. In addition, eDNA is easily hydrolyzed by biological and chemical processes [74,75] and would theoretically represent one of the most labile fractions of organic carbon in biomass. It is still unknown as to how COI copy number reflects kelp biomass as it degrades over time, but it is likely to follow an exponential decay pattern, with persistence being heavily dependent on starting concentration [76,77]. It is reasonable to assume that the kelp DNA was relatively intact in the fresh finely-ground kelp biomass that was lyophilized and used in the biomass spiking experiment. In this regard, our relationship between COI gene copy number and kelp biomass may represent a conservative underestimate of kelp biomass in marine sediment, since eDNA in environmental samples is likely to be in various stages of degradation and/or complexation with minerals and organics that may or may not be available for PCR amplification. For our study site, we assumed that the bulk of kelp biomass reaching the seabed under the farm would experience minimal transport in the water column and thus be relatively undegraded, given the shallow depth of our study site (~10m) and estimated sinking rates of different forms of kelp biomass [53]. However, substantial degradation may still occur during the process of burial, which may also alter the relationship between eDNA and kelp carbon beyond what we have determined in this study. In addition, the carbon content of kelp can be variable depending on life stage, life history and growth conditions, such as temperature and nutrients, and is likely to be affected by climate change as well [78]. How this may affect the relationship between kelp eDNA and bulk biomass, much less kelp carbon is unknown at present.

It is worth noting that a current major limitation of eDNA as an MMRV tool for mCDR is that there is insufficient evidence to show that eDNA from seaweeds can last long enough and in readily detectable quantities in marine sediments to satisfy expectations of permanence, on climate-relevant timescales of centuries or greater. This is even more relevant for high-integrity voluntary carbon markets, where conservative estimates of mCDR effectiveness are prudent. Molecular tools from the field of ancient DNA examining the degradation status of eDNA over time [79], applied to deeper cores from offshore sediments corresponding to such timeframes, may prove useful in working towards addressing this issue. Ultimately, it will be necessary to demonstrate that eDNA as a proxy can be used to accurately reflect biomass (and by extension, carbon content) over time in a systematic manner, before it can become a fully-fledged tool in MMRV.

Another finding from this work is the recognition that *S. latissima* eDNA is highly patchy in marine sediments both across the seabed, and with depth in the sediment, likely reflecting local heterogeneity in biomass deposition, burial, and subsequent degradation. Even though we only found clear evidence of greater kelp eDNA presence within the farm in 2022, there were clearly sediment cores in both years with markedly higher kelp gene copy numbers than the baseline. Site 2 (2022), for example, was close to a freshly harvested kelp growth line and showed markedly higher COI values than the other sites inside the farm in that year. Overall, levels of *S. latissima* gene copies were quite consistent outside the farm (and in the unamended sediment from the fully marine Damariscotta River used in the kelp spiking experiment, where *S. latissima* is known to be present); and were more variable inside the farm. In addition, using the effect size of site type on COI from our OLS (standardized beta = 0.56), which is relatively high (~0.5 = high; ~ 0.3 = moderate; ~ 0.1 = low), a power analysis conducted as a thought experiment (not shown in results) showed that a minimum sample size of about 14 samples is required to determine if any difference in COI values between site types is genuine and significant, for a significance value of 0.05 and statistical power of 0.8. This is just shy of the number of cores we sampled in this study in 2022; 15. This indicates that an effective sampling scheme to detect kelp biomass sequestered in sediment should include more extensive sampling from inside a farm (or other study sites) to account for signal heterogeneity, while more limited sampling outside a farm may be sufficient.

### Impacts of the kelp farm on benthic ecology

#### Nutrient impacts.

In both years, there was no conclusive effect of site type on any of the individual nutrient metrics measured. There was no evidence of enhanced sediment organic carbon (or total carbon content) in both years of sampling. While the farm sediment in 2022 showed a significant effect of site type on TN%, the mean TN% value of sediments inside the farm was only marginally higher than that of sediments outside the farm (by about 4.6%). Fresh kelp biomass is known to have relatively high C:N ratios for macroalgae [80], which in turn decreases throughout the degradation process, leading to a greater relative nitrogen content [50]. However, the estimated kelp carbon values derived from our eDNA gene copy numbers represent a miniscule proportion of observed sediment TOC content (~0.0013%), and hence, kelp-derived nitrogen enrichment is likely to be minimal, especially compared to other sources, such as planktonic and benthic microalgae.

Several studies in the literature have attempted to link gene copy numbers or read counts to sediment carbon values in a blue carbon context, but focusing on natural sediments that are not associated with farming activity [44,47,69,70,81,72,73,82]. On the other hand, a small number of studies have aimed to investigate the carbon content of sediments associated with kelp farming [44,83,84]. Only one study [44] was able to find significant enrichment in organic carbon in sediments underlying kelp farms (across a variety of scales, ages, seaweed species, and geographical locations), although it is worth noting that integrated multitrophic aquaculture (IMTA) systems involving macroalgae are known to exhibit enhanced organic carbon in underlying sediments, even in relatively low-impact IMTA settings such as bivalve-macroalgal co-culture [85,86].

#### Impacts on sediment community composition.

Overall, differences in prokaryotic and eukaryotic taxa inside and outside the farm were relatively minor, indicating the presence of this kelp farm had minimal impact on the benthic community. Both 2022 16S and 18S datasets showed statistically significant, albeit minor differences in the Shannon Index, with alpha diversity being lower in sediments inside the farm, which may reflect some sort of kelp farm-associated effect. However, given the lack of evidence of increased organic carbon (or total carbon) content, and some ambiguous evidence of increased nitrogen content in sediments inside the farm, it is unclear as to whether nutrient enrichment is responsible for such an effect.

The more robust coring strategy used in 2022 did detect some statistically significant differences in community composition driven by site type. This sampling strategy included more sites inside the farm compared to outside; and sampled near sites where kelp was visibly present on the seafloor. Nevertheless, in 2022 both the effect sizes of significant factors in the PERMANOVAs and the explained variance on the axes in the PCoAs are relatively small (<15% explained variance on both PCoA axes, for all marker genes and years. From the PERMANOVA results, depth was by far the most important factor structuring biodiversity in the sediments (e.g., 3-4x the ω2 value of site type), which is to be expected given the strong redox gradients in such fine organic matter rich sediment, where the oxic layer is expected to extend only millimeters, barring extensive bioturbation. This depth effect is consistent with other studies of community composition in marine sediments [64–78].

To the best of our knowledge, there are no other studies looking at 16S and 18S-based analyses of shallow, subtidal, temperate marine sediments on the Maine coast. Looking at prokaryotes, the dominant members of the sediment microbiome were consistent with intertidal Maine sediments [87] and eastern China subtidal muddy sediments [88]. These groups include many bacteria associated with sulfur cycling (e.g., *Desulfosarcinaceae, Desulfobulbaceae),* as well as *Gammaproteobacteria.* No prominent potentially pathogenic groups were identified. Overall, the prokaryotic diversity inside and outside the farm displayed remarkable consistency as illustrated in the bar plots of prokaryotic diversity (Fig 4). Nonetheless, the volcano plots identified prospective indicator taxa for kelp farm inputs in the 16S-based prokaryotic community, although these taxa were of low read abundance. It is challenging to assign functional roles to these potential indicator taxa since we lack a detailed understanding of their physiological diversity. Nonetheless, there were some interesting correlations between the presence of kelp biomass (using eDNA as a proxy), and bacterial taxa, for example the family *Flavobactericaea*e, known to be associated with degradation of complex organic matter (e.g., macroalgal biomass) [89]. More studies will be required to determine if such taxa are truly indicative of active kelp biomass degradation.

As for eukaryotes, the only prominent taxa in our study in common with similar systems were *Dinophyceae* [90] and *Islandinium* [87], with both studies being from intertidal Gulf of Maine sediments, rather than subtidal sediments as in our study. Similar to prokaryotes, the strongest driver of diversity was sediment depth. In terms of indicator taxa for the presence of kelp, e.g., taxa more enriched inside the farm from the 2022 dataset, the polychaete *Heteromastus* was a prospective indicator taxon, appearing to be markedly more common in sediments inside the farm in the bar plots (Fig 7). Infaunal polychaetes are known to be associated with nutrient enrichment in marine sediments [91], thus increased abundance of *Heteromastus* eDNA inside the farm could be consistent with some enrichment of organic matter in these sediments. Nonetheless, the patchiness of polychaete eDNA and the lack of evidence of nutrient enrichment based on total C or N makes it difficult to draw clear conclusions. Larger eukaryotes may also have a tendency to be simultaneously over-sampled and under-sampled in eDNA based studies, the former if greater amounts of shed eDNA are found in a sample due to their relatively larger size, and the latter due to their relative scarcity in bulk sediment compared to smaller organisms. For the other kelp farm studies looking at benthic invertebrates, the impact on such communities was minimal to slightly positive in terms of both species abundance and diversity, and derived benthic quality indicators [34,35]. Again, more kelp farms and samples will need to be analyzed to draw clearer conclusions regarding increased polychaete abundance associated with kelp farm sediments.

The only common macrophyte (and blue carbon contributor) that we were able to identify in our 18S dataset was the common eelgrass, *Zostera marina*, and only in relatively low proportions, yet still large enough to be visually distinctive in the bar plots (~5% of ASVs). We observed an eelgrass bed near the intertidal zone of Chebeague Island, approximately ~400–500 m from the farm, however, no visible eelgrass biomass was seen in the sediment cores. As a vascular plant, eelgrass biomass is known to be more resistant to degradation than macroalgal biomass [92]. Eelgrass biomass can be exported and potentially contribute to carbon sequestration in allochthonous sediments [69,70,73], both coastal and further offshore. The absence of ASVs corresponding to *S. latissima* and other *Phaeophyceae* is likely to reflect genuine low abundance in the study sediments rather than primer biases, as the Earth Microbiome 18S primer set that we used has been validated to be effective on these taxa in other studies in the eastern North Atlantic [81,72].

## Concluding remarks

Despite a targeted sampling effort aiming to sample at sites where kelp biomass was visibly observed accumulating on the seabed, the actual magnitude of this relatively small and young kelp farm “effect” in terms of various eDNA indicators and derived kelp carbon is minimal and may only be detectable with a larger sampling effort. From an environmental impact perspective, it is clear that the effect of this young, small kelp farm on underlying sediments is relatively minimal, which concurs with the findings of traditional biodiversity surveys [35]. This may be heartening to farmers who would like to be certain that their livelihoods have minimal impact on the surrounding benthos.

In addition, the results of this study may not discount the possibility of shed and discarded biomass from the natural operations of kelp farming contributing to underlying carbon sequestration per se, as the farm in this study, while large for by New England standards (~1.25 ha), was relatively small in comparison to farms in other parts of the world, including Alaska (e.g., 100 acres/ ~ 40 ha for Seagrove Kelp Co. circa 2021 [NOAA Fisheries]) and Northwestern Europe (e.g., 18 ha [34]), not to mention north-east Asia (which has the oldest, largest kelp aquaculture industries in the world, e.g., 15000 ha in Ningde, China, 320 years of operation in Tokyo Bay, Japan [44]). Should kelp farming scale up in the western world, further eDNA and non-eDNA analytical methods will be necessary to confirm if the findings in this paper are still valid.

The patchiness of eDNA in the environment also indicates the importance of concerted sampling design prior to any large-scale environmental sampling. This may require comprehensive power-analysis pilot studies to determine how many samples and replicates to collect and process from field sampling all the way up to molecular analytical methods (e.g., DNA extraction replicates, PCR replicates, sequencing replicates). Depending on the taxa of interest and the consequences of false positives and false negatives, this may be necessary to allow study designers to be confident about detecting the presence of taxa of interest, as well as the distance (and/or) environmental gradients to collect samples along.

Nevertheless, the ability of both species-specific qPCR-based approaches and community-wide metabarcoding approaches to identify subtle signals of known environmental influence in marine sediments, which traditional bulk nutrient analyses could not, and at lower cost is highly promising. This indicates the potential for eDNA to become a common part of future environmental monitoring toolkits, whether blue carbon or otherwise, provided sufficient site and taxon-specific optimization and proper sampling strategies are implemented.

## Supporting information

S1 File Supplementary document for the manuscript [93,94]. (DOCX)
