# Supplementary material for: Using eDNA tools to examine the impact of kelp farming on underlying sediments
Source: PLoS One. 2025 Sep 5;20(9):e0331416. doi: 10.1371/journal.pone.0331416 (PMC12412952; doi:10.1371/journal.pone.0331416)
Supplement: S1 File — (DOCX) [file pone.0331416.s001.docx]

# **Supplementary Document S1**

## **Section A: Sample site details**


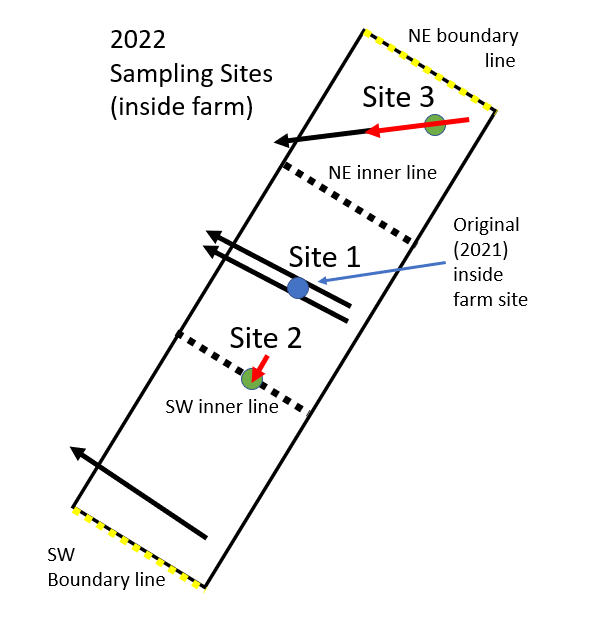


**Supplementary Figure A1**: A map of the three sites (1, 2, 3) within the kelp farm and their relative locations compared to each other and lines within the farm. The dotted boundary lines and inner lines are part of the structure of the farm, and the kelp itself is grown on growth lines (7+ lines) spanning the long length of the farm (i.e. the longer, non-dotted lines). The arrows represent transects surveyed by ROV, with the red arrows representing where kelp detritus was observed on the seabed. The blue dot represents the original farm site in 2021, and the green dots represent the additional sites sampled in 2022 based on ROV footage of kelp. Tidally driven flow runs NE to SW, back and forth on the ebb and rising tides. Further information on the lease site (Site ID: CAS CHEB2) can be found on the Maine Department of Marine Resources (DMR) [website](https://www.maine.gov/dmr/aquaculture/maine-aquaculture-leases-and-lpas/aquaculture-web-map).

**Supplementary Table A1**: Details of sites sampled in this study

| Site No. | Site Type | Latitude | Longitude | Years sampled | ROV detection of kelp (2022) | Comments |
| --- | --- | --- | --- | --- | --- | --- |
| 1 | Inside | 43.7213 | -70.1474 | 2021, 2022 | Absent | Inside reference site, middle of farm |
| 2 | Inside | 43.7211 | -70.1479 | 2022 | Present | Next to SW inner line |
| 3 | Inside | 43.72229 | -70.1464 | 2022 | Present | Next to NE boundary |
| 4 | Outside | 43.72409 | -70.1457 | 2021, 2022 | Absent | Outside reference site |
| 5 | Outside | 43.72272 | -70.1489 | 2022 | Absent | Outside farm, to west |

## **Section B: qPCR assay development**

***In-silico* primer and probe development**

Six different mitochondrial, chloroplast, and nuclear gene markers (COI, ITS, NAD5, COB, rbCL, COX3) were initially examined to identify a candidate gene with the greatest interspecific variation.

Sequences for *S. latissima*, related kelp taxa and common Gulf of Maine macroalgal taxa (i.e., potential off-target taxa, Supplementary Table B1) were collated from GenBank and evaluated in Benchling v 1.01 (Benchling). The mitochondrial cytochrome oxidase subunit I (COI) was chosen as the best candidate based on interspecific genetic variation and used to create a Taqman® minor groove binding (MGB) qPCR assay using Allele ID v 7.8 (PREMIER Biosoft).

**Supplementary Table B1:** Brown intertidal and subtidal macroalgal COI sequences used for the development of the COI qPCR assay, once COI was chosen as the gene of interest. Species were selected based on phylogenetic similarity to *S. latissima* or overlapping distribution in the Gulf of Maine.

| Taxon | Farmed? | Distribution | GenBank Accession Number |
| --- | --- | --- | --- |
| *Ascophyllum nodosum* | No | Intertidal | EU681390 |
| *Agarum clathratum* | Can be | Subtidal | LC148146 |
| *Alaria esculenta* | Yes | Subtidal | MN816900 |
| *Desmarestia aculeata* | No | Subtidal | AB776654 |
| *Desmarestia viridis* | No | Subtidal | AY500367 |
| *Fucus distichus* | No | Intertidal | KY678904 |
| *Fucus spiralis* | No | Intertidal | MG922856 |
| *Fucus vesiculosis* | No | Intertidal | AY494079 |
| *Laminaria digitata* | Yes | Subtidal | AJ344328 |
| *Hedophyllum nigripes* | No | Subtidal | MH327950 |
| *Saccharina japonica* | Yes | Subtidal | AP011493 |

The resulting qPCR assay amplified a 147 base pair product. The probe was labeled on the 5’-end with the fluorescent reporter dye 6-carboxyxfluoroescein (FAM) and a MGB-Eclipse Quencher (MGBE-Q) on the 3’ end to prevent amplification in the absence of the target sequence.

**Assay development**

The assay was initially developed as a conventional qPCR assay. All reactions were performed on a BioRad CFX-1000 instrument. Each 20μL reaction consisted of 10 μL IDT PrimeTime Gene Expression Master Mix, 3 μL of 10uM forward primer (1.5 μM final concentration), 2 μL of 10 uM reverse primer (1 uM final concentration), 1 μL of 10mM probe (500 nM final concentration), and 4 μL of the diluted sample.

Thermal cycler settings used in this study were as follows: initial denaturation at 95 ℃ for 3 minutes, followed by 39 cycles: denaturation at 95 ˚C for 10 seconds, annealing step at 53.4 ˚C for 30 seconds and extension at 72 ˚C for 20 seconds. A final extension step was used at 72 ˚C for 5 minutes to complete amplification.

**Standard curve**

To generate a standard curve, the PCR product containing the 147 base pair *S. latissima* COI amplicon was inserted into a pCR4-TOPO® cloning vector and cloned into One Shot® TOP-10 competent cells using the TOPO TA Cloning Kit for Sequencing, following manufacturer’s instructions. The linearized plasmid was run on a 0.8% TAE gel and isolated with the Zymoclean™ Gel DNA Recovery Kit (Zymo Research). The concentration of the linearized plasmid and its molecular weight (verified from its nucleotide composition via Sanger sequencing), were used to determine the concentration (gene copy number per μL) of the product. The linearized plasmid was used to make a standard dilution series spanning 7 orders of magnitude, from 2.73 gene copies per μL to 27.3 million gene copies per μL (Supplementary Figure B1). For the standard curve presented in this manuscript, which was used for runs on actual samples, a standard dilution series spanning 5 orders of magnitude, from 2.73 gene copies per μL to 273 000 gene copies per μL was deemed sufficient.


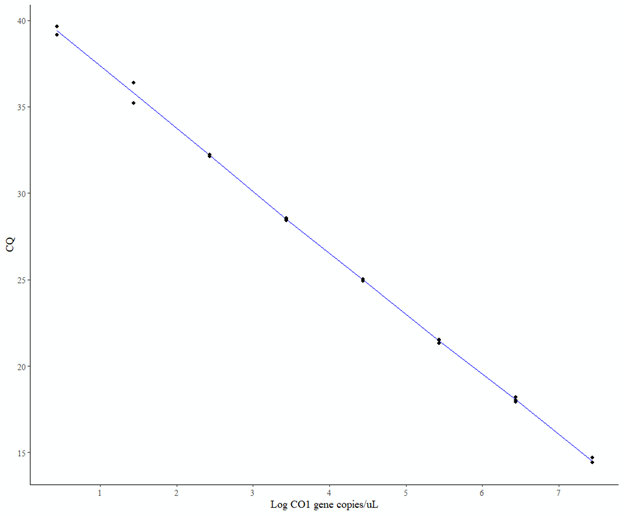
**Supplementary Figure B1:** Original standard dilution series showing correlation between log COI gene copy number per μL and cycle threshold value (Cq). [y = -3.549x + 40.821, R^2^ = 0.999, p < 0.00001]. Excluding the NTC (the lowest level on the plot), the dilution series spanned 7 orders of magnitude from 2.73 gene copies/μL up to 27.3 million gene copies/μL.

qPCR technical replicates were used to calculate mean copy number for each sample. Standard curve performance was evaluated by fitting a linear regression to Cq values against log gene copy number per μL of qPCR reaction.

The standard dilution series showed a significant positive relationship between cycle threshold (Cq) value and gene copy number (R^2^ = 0.999, p <0.00001; y = -3.197x + 37.049) (Supplementary Figure B2).


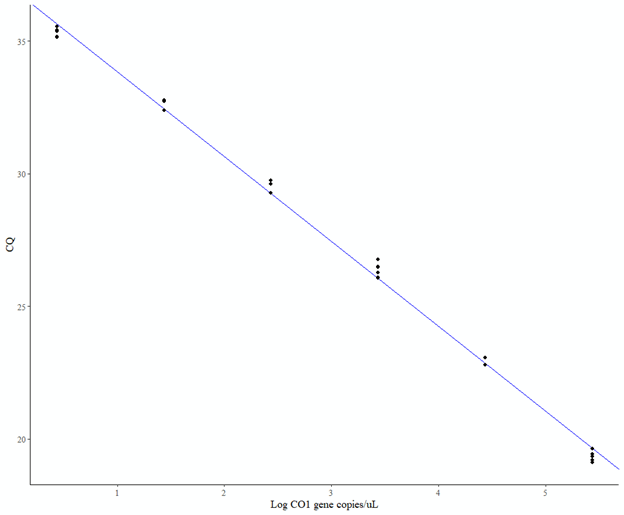


**Supplementary Figure B2:** Standard dilution series using the IDT PrimeTime Gene Expression Master Mix formula showing correlation between log COI gene copy number per μL and cycle threshold value (Cq). [y = -3.197x + 37.049, R^2^ = 0.999, p < 0.0001]. Excluding the NTC, the dilution series spanned 5 orders of magnitude from 2.73 gene copies per μL up to 273 thousand gene copies/μL.

**Cross-reactivity tests**

Primer set specificity was tested in-silico using NCBI Primer-Blast and was shown to only amplify *S. latissima* (Supplementary Table 1). To further verify species-specificity, an initial cross-reactivity test was conducted. Each reaction was prepared as follows: 10 μL 2x PerfeCTa SYBR Green FastMix, 1 μL of both forward and reverse primers at 500 nM final concentration, 6 μL MilliQ water and 2 μL sample (1x DNA, 1:10 DNA, 1:00 DNA or MilliQ water). This test included 32 total physical samples, including 9 samples of *S. latissima* sourced from different sites in the Gulf of Maine, 9 samples of common local brown macroalgae species (family Phaeophyceae), and 14 samples of common local red macroalgae species (phylum Rhodophyta), see Supplementary Table B2 for species designations. All algal DNA was extracted using the Qiagen Plant Pro Kit, following the manufacturer’s protocols. To decrease the chance of PCR inhibition, all DNA extractions were diluted 1:10 and 1:100. Cross reactivity between the *S. latissima* qPCR probe and the different species was tested with a melt curve analysis, with an initial melt temperature of 65 ˚C for 5 seconds that increased by 0.5 ˚C up to 95 ˚C with a plate read taken every 5 seconds. The non-*S. latissima* reactions that exceeded baseline RFUs were sent for Sanger sequencing of the COI target sequence to Eurofins Genomics USA (Louisville, KY) to determine if these positive reactions were due to off-target amplification, or contamination of the sample with *S. latissima* DNA. An additional cross-reactivity test was conducted on the taxa listed in Supplementary Table B2 by pooling all the positive qPCR COI amplicons and concentrating them using a gDNA Clean & Concentrator-25 Kit (Zymo Research). The pooled sample was sent to Genewiz (Amplicon-EZ service, Azenta, South Plainfield NJ) for metabarcoding of the COI gene to determine if non-*S. latissima* amplicons were present.

**Supplementary Table B2:** Taxa tested in the cross-reactivity tests based on common brown and red macroalgal taxa in the Gulf of Maine. Test 1 and Test 2 are two independent cross reactivity tests, with species tested differing based on DNA extracts available at the time. All samples that had a positive result (Yes) were shown to contain *S. latissima* DNA, see Methods and Results for details. Verification of species names and distribution corroborated with Mathieson *et al*. 2008 [96].

| Taxon | General descriptor | S. latissima DNA present  Test 1. Test 2 | |
| --- | --- | --- | --- |
| *Agarum clathratum* | Brown subtidal alga (kelp), farmed | Yes | Yes |
| *Alaria esculenta* | Brown subtidal alga (kelp), farmed | Yes | Yes |
| *Ascophyllum nodosum* | Brown intertidal alga (fucoid) | Yes | Yes |
| *Callophyllis cristata^1^* | Red subtidal alga | Yes | No |
| *Ceramium spp.^2^* | Red intertidal & subtidal algae | Yes | No |
| *Chondrus crispus* | Red intertidal alga | Yes | No |
| *Dasysiphonia japonica* | Red intertidal alga, invasive | No | Yes |
| *Desmarestia aculeata* | Brown intertidal alga | No | Yes |
| *Euthora spp.* | Red alga | No | Yes |
| *Ectocarpus fasiculatus* or *siliculosus* | Brown intertidal & subtidal alga | No | Yes |
| *Fucus spiralis* | Brown intertidal alga (fucoid) | Yes | Yes |
| *Fucus vesiculosus* | Brown intertidal alga (fucoid) | Yes | Yes |
| *Gracilaria tikvahiae* | Red intertidal alga | No | Yes |
| *Grateloupia turuturu* | Red intertidal alga, invasive | Yes | No |
| *Hedophyllum nigripes^3^* | Brown subtidal alga (kelp) | Yes | No |
| *Laminaria digitata* | Brown subtidal alga (kelp), farmed | Yes | Yes |
| *Lomentaria clavellosa* or *orcadensis* | Red intertidal & subtidal algae | Yes | No |
| *Membranoptera fabriciana* or *alata* | Red intertidal algae | Yes | No |
| *Palmaria palmata* | Red intertidal alga, can be farmed | Yes | Yes |
| *Polyides rotundus* | Red intertidal & subtidal alga | Yes | No |
| *Polysiphonia stricta* | Red intertidal & subtidal alga | No | Yes |
| *Saccharina latissima* | Brown subtidal alga (kelp), farmed | Yes | Yes |
| *Saccharina latissima* var. *angustissima* (skinny kelp) | Brown subtidal alga (kelp), farmed, possibly endemic | Yes | Yes |
| Assorted benthic diatoms (e.g., Family: *Pleurosigmataceae*) | Diatom | No | Yes |

^1^ Formerly known as *Euthora cristata*

^2^ Possible species: *cimbricum, deslongchampsii, virgatum*

^3^ Formerly known as *Saccharina nigripes* or *Laminaria nigripes*

In-silico analyses on Genbank sequences showed that the COI primer set would not amplify other species besides *S. latissima*, with congenerics having an average of 1-3 base pair mismatches for the forward primer, and 1-2 mismatches for the reverse primer, Supplementary Figure 5. No congeneric taxa are present in the Gulf of Maine and non-Saccharina Laminariales showed a minimum of 3 mismatches for both forward and reverse primers.

In-vitro cross-reactivity testing of non-*S. latissima* reactions resulted in some single-digit amplification in several samples from two independent tests. (Supplementary Table B2). Melt curve analysis, Sanger sequencing and metabarcoding of the COI gene from a pooled sample of all positive amplifications on non-target species contained reads only from *S. latissima*, again confirming that these amplifications were false positives resulting from small amounts of contamination from *S. latissima* tissue. A sample enriched in benthic diatoms from the Lowe’s Cove site was also quantified to confirm that this often highly abundant community of benthic epifauna would not cross react with the assay. No amplification was detected in the benthic diatom sample.

**qPCR limit of detection (LOD) and limit of quantification (LOQ)**

The limit of detection (LOD) and limit of quantification (LOQ) for the assay was determined using fresh dilutions of the plasmid standard from stock solution, diluted in the elution buffer from the gDNA Clean & Concentrator Kit. Steps of 5 gene copies per μL (0, 5, 10, 15) were tested, with a minimum of 15 replicate reactions quantified per step. Steps were chosen based on variation in Cq values from standards and no-template controls (NTCs) in previous qPCR runs. The threshold for LOD was set at a 95% probability of positive detection, and the threshold for LOQ was set at a coefficient of variation (CV) of 25% (Kralik & Ricchi, 2017).

A one-way Analysis of Variance (ANOVA) was used to compare Cq values between different tested levels. Model residuals were confirmed to fulfill ANOVA assumptions using standard residual diagnostic plots.

The LOD and LOQ of the assay was tested to be at most 5 gene copies per μL of qPCR reaction. A post-hoc Tukey-Kramer test following a significant one-way ANOVA (F(2, 37) = 21.51, p < 0.00001) comparing the four tested levels, (0, 5, 10, 15 copies/μL) found all four levels to be significantly different from one another at the 95% confidence interval (Supplementary Table B3). Coefficients of variance for the three levels tested were all below the commonly-accepted LOQ threshold of 25% (5 copies = 23.5%; 10 copies = 17.5%, 15 copies = 22.4%).

**Supplementary Table B3:** Posthoc Tukey-Kramer test comparisons between the four levels tested in the Limit of Detection + Limit of Quantification tests.

| Levels | p-value |
| --- | --- |
| 0 vs 5 copies/μL | 0.000148 |
| 0 vs 10 copies/μL | 0.0000564 |
| 0 vs 15 copies/uL | 0.0000001 |
| 5 vs 10 copies/μL | 0.00292 |
| 5 vs 15 copies/μL | 0.0000003 |
| 10 vs 15 copies/μL | 0.0151 |

No LOD and LOQ testing was necessary for the dPCR, given that the theoretical value of both parameters is a single gene copy.

**qPCR-dPCR comparisons**

In general, for the kelp biomass spiking experiment, gene copy number ratios between the same samples run on dPCR versus qPCR were 0.555 +/- 0.0178 (y = 0.555x, R^2^ = 0.94, F(1, 29) = 971.8, p = < 0.0001) (Supplementary Figure B3).


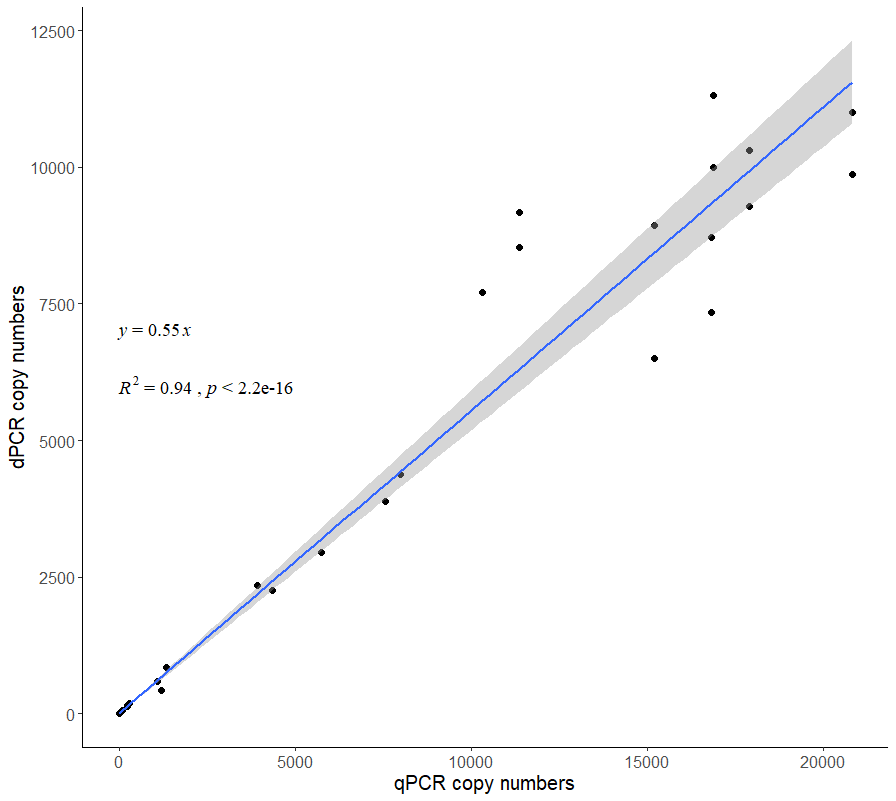


**Supplementary Figure B3**: Ratio of dPCR to qPCR values from the samples in the kelp biomass spiking experiment. The blue line and associated model output represent the OLS regression fitted to the dataset, with the gray borders as the 95% confidence interval. The slope of the regression line between the two variables is 0.555 +/- 0.0178.

## **Section C: Supplementary material for the main body of the manuscript**

1. $Maximum DNA extraction COI gene copy yield (with clean-up)$

$$= \frac{Mean dPCR copy number}{dPCR sample DNA concentration}\times\frac{Clean-up kit eluate concentration}{Clean-up kit efficiency}\times Clean-up kit eluate volume$$

$$\times\frac{PowerSoil eluate volume}{PowerSoil eluate volume used in clean-up}$$

2. $Maximum COI copy number per gram of dry sediment$

$$= \frac{Maximum DNA extraction yield}{Weight of sediment extracted}\times Proportion of buffer subsampled during each extraction step \times Sediment wet weight:dry weight ratio$$

**Supplementary Figure C1:** Formulae used for the calculation of COI gene copy number per gram of dry sediment, from dPCR mean copy number data. For the sake of conservatism, gene copy number per gram of sediment was calculated with the assumption that none of the extracted eDNA was lost during the extraction and clean-up process, except when extraction inefficiencies are clearly known, and when known volumes of various eluates were subsampled. Extraction efficiencies for all samples were assumed to be identical, regardless of method used. The maximum theoretical recovery efficiency for the Zymo kit was used (95%). Volumes of buffers used within each step of the PowerSoil kit were also accounted for in calculations. Hence, the resultant gene copy numbers per gram of dry sediment represent an upper limit estimate based on the gene copies in each dPCR reaction.

1.$Mass of dry kelp carbon (g) per unit volume of sediment [1 cm^{3}(2021)]$

$SUM(mean COI gene copies for every sampled layer of sediment) x (1/1.3 x 10^{10}) /$

$$SUM(Depths sampled for eDNA in cm)$$

**Supplementary Figure C2:** Formulae used to calculate derived grams of kelp carbon per unit volume of sediment in kelp farm sediment from 2021 and 2022. The value of 1.3 x 10^10^ is derived from the mean COI to dry kelp biomass equation in Figure 1. While not all depth intervals from 0 cm to the maximum sampled depth for eDNA have been sampled at high resolution, coverage is assumed to be sufficient to cover the slight degree of extrapolation needed within that range.

**Supplementary Table C1:** Mean COI values per gram of dry sediment, and per dPCR reaction, including standard deviation, at every treatment level in the kelp biomass spiking experiment, including the Great Salt Bay controls and the 5% wet kelp biomass/0.20625% dry kelp carbon treatment level not presented in Figure 1. Both wet kelp biomass values and dry kelp carbon values are presented in this table. Note that despite the seemingly high mean COI values in the Great Salt Bay and 0% kelp treatments, this corresponds to low single digit copy numbers per dPCR reaction, or sub-single digit in 2 out of the 3 Great Salt Bay treatment replicates. While a case can be made for coercing the Great Salt Bay values to zero, we have opted to treat these values as if they were true positives instead for all calculations.

| Added dry kelp carbon (%) | Added wet kelp biomass (%) | Mean COI/gram of dry sediment (1 d.p.) | Standard deviation | Mean COI/dPCR reaction (3 s.f.) | Standard deviation |
| --- | --- | --- | --- | --- | --- |
| 0% (Great Salt Bay) | 0% (Great Salt Bay) | 12189.9 | 78900.0 | 1.053 | 0.810 |
| 0% | 0% | 134070.8 | 73613.8 | 2.894 | 1.700 |
| 0.000413% | 0.01% | 1813470.7 | 263601.8 | 54.712 | 5.784 |
| 0.0020625% | 0.05% | 5698006.3 | 1992860.5 | 158.590 | 27.960 |
| 0.004125% | 0.1% | 32504170.8 | 9982033.2 | 626.412 | 212.581 |
| 0.020625% | 0.5% | 154755452.8 | 15104043.4 | 2517.426 | 378.440 |
| 0.04125% | 1% | 375964072.9 | 208426955.6 | 5325.796 | 2078.299 |
| 0.0825% | 2% | 1160226746.6 | 214742805.8 | 9106.923 | 1247.726 |
| 0.20625% | 5% | 1419011618.0 | 244721583.9 | 9390.512 | 1639.881 |

**
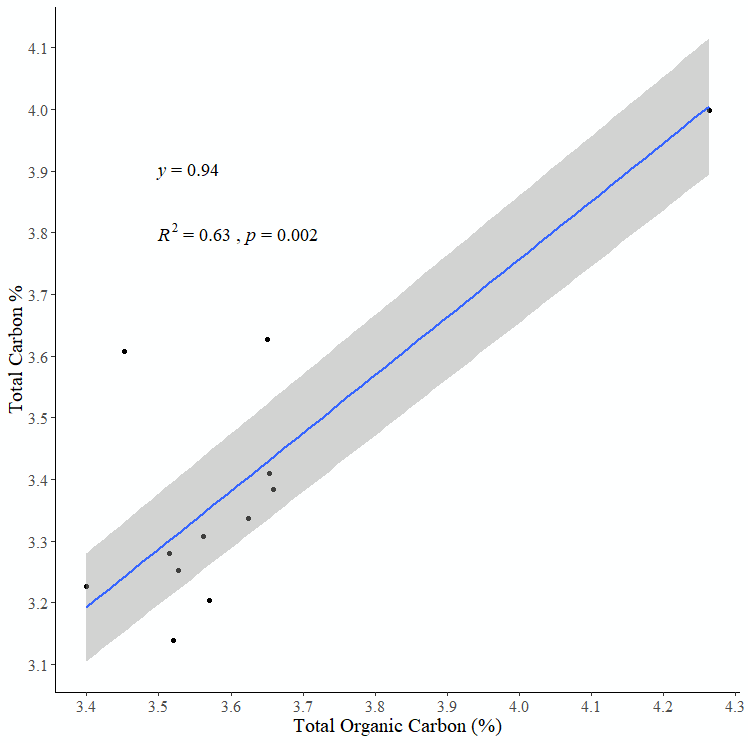
**

**Supplementary Figure C3:** Plot of total carbon % (TC%) against total organic carbon % (TOC%) for the 12 (out of 45) 2022 kelp farm samples analyzed for both TC% and TOC%.

The linear regression fitted to TC% against TOC% had an equation of y = 0.94x (p = 0.00203, R^2^ = 0.631). Strangely, TOC% was often slightly higher than TC% for the same samples (with a mean ratio of 1.066 +/- 0.043), but that may have been due to the fact that different subsamples were run for both metrics. The same was true of TN% and post-acidified N% (mean ratio of 1.024 +/- 0.057). Regardless, Kruskal-Wallis tests on both TOC% against TC% and post-acidified N% against TN% yielded non-significant results (p = 0.443 for both sets of metrics). For simplicity, inorganic carbon content in the sediments was considered to be minimal enough for TC% to approximate TOC%, likewise TN% was considered to approximate post-acidified N%


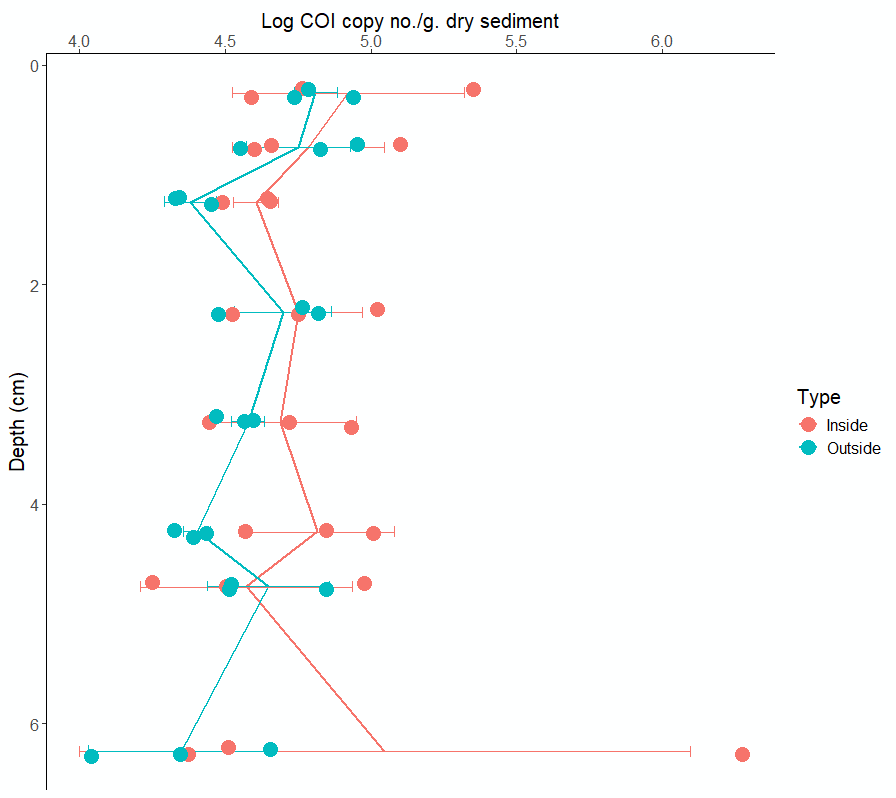


**Supplementary Figure C4:** *Log S. latissima* COI gene copy number by depth for the 2021 samples. The same interpretation of the plot applies as in Figure 2.

**Supplementary Table C2:** Summarized tabulated data for the 2021 COI plot in Supplementary Figure C4.

| Site Type | Depth (cm) | Mean log COI gene copy no. | S.D. |
| --- | --- | --- | --- |
| Inside | Aggregated | 4.773 | 0.408 |
| Inside | 0.25 | 4.922 | 0.397 |
| Inside | 0.75 | 4.785 | 0.260 |
| Inside | 1.25 | 4.601 | 0.078 |
| Inside | 2.25 | 4.751 | 0.219 |
| Inside | 3.25 | 4.690 | 0.259 |
| Inside | 4.25 | 4.815 | 0.265 |
| Inside | 4.75 | 4.572 | 0.364 |
| Inside | 6.25 | 5.048 | 1.048 |
| Outside | Aggregated | 4.577 | 0.221 |
| Outside | 0.25 | 4.810 | 0.074 |
| Outside | 0.75 | 4.752 | 0.178 |
| Outside | 1.25 | 4.381 | 0.089 |
| Outside | 2.25 | 4.698 | 0.168 |
| Outside | 3.25 | 4.579 | 0.057 |
| Outside | 4.25 | 4.403 | 0.047 |
| Outside | 4.75 | 4.648 | 0.210 |
| Outside | 6.25 | 4.349 | 0.320 |


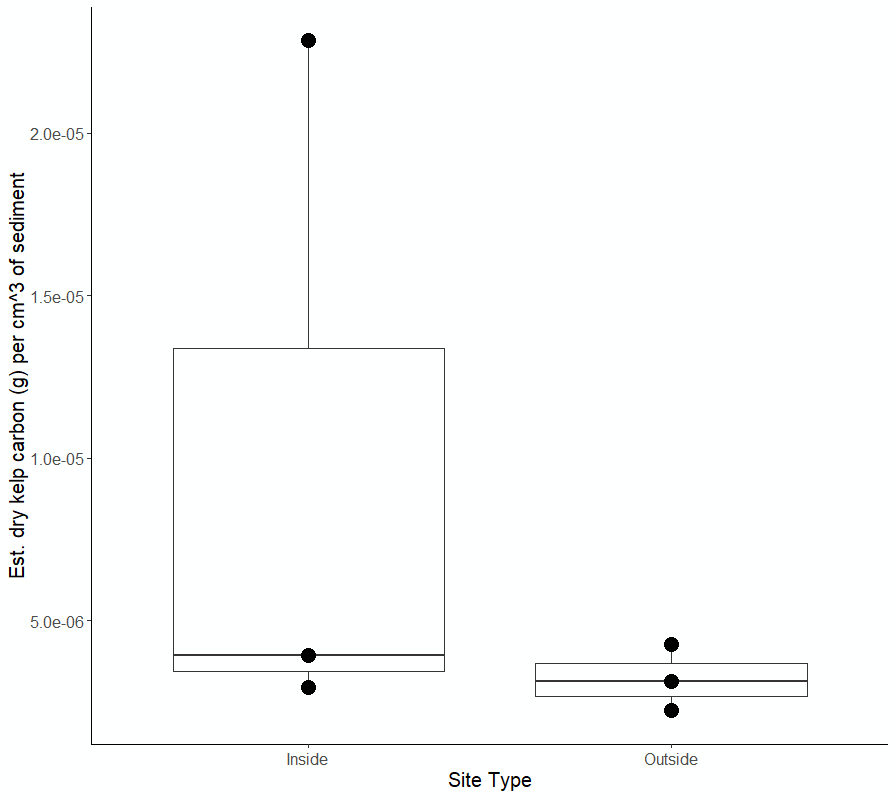


**Supplementary Figure C5**: Box plot of derived grams of *S. latissima* carbon in the 2021 cores per 1cm^3^ volume of sediment, integrated down to the maximum depth sampled for eDNA (6.5 cm). The same interpretation of the plot applies as in Figure 3.

**Supplementary Table C3:** Tabulated data for the 2021 integrated kelp carbon plot in Supplementary Figure C5

| Site Type | Est. dry kelp carbon (g) / cm^3^ of sediment | S.D. |
| --- | --- | --- |
| Inside | 9.901 e-06 | 1.122 e-05 |
| Outside | 3.211 e-06 | 1.018 e-06 |

**Supplementary Table C4:** Summarized tabulated data for the 2022 COI plot in Figure 2.

| Site Type | Depth (cm) | Mean log COI gene copy no. | S.D. |
| --- | --- | --- | --- |
| Inside | Aggregated | 4.816 | 0.391 |
| Inside | 0.5 | 5.047 | 0.368 |
| Inside | 1.5 | 4.921 | 0.367 |
| Inside | 2.5 | 4.826 | 0.390 |
| Inside | 4.5 | 4.606 | 0.264 |
| Inside | 6.5 | 4.680 | 0.454 |
| Outside | Aggregated | 4.506 | 0.211 |
| Outside | 0.5 | 4.677 | 0.182 |
| Outside | 1.5 | 4.600 | 0.275 |
| Outside | 2.5 | 4.530 | 0.187 |
| Outside | 4.5 | 4.390 | 0.086 |
| Outside | 6.5 | 4.335 | 0.101 |
| Site 1 | Aggregated | 4.669 | 0.362 |
| Site 2 | Aggregated | 5.101 | 0.391 |
| Site 3 | Aggregated | 4.678 | 0.255 |
| Site 4 | Aggregated | 4.549 | 0.188 |
| Site 5 | Aggregated | 4.463 | 0.231 |

**Supplementary Table C5:** Summarized tabulated data for the 2022 integrated kelp carbon plot in Figure 3.

| Site Type | Est. dry kelp carbon (g) / cm^3^ of sediment | S.D. |
| --- | --- | --- |
| Inside | 2.461 e-04 | 1.974 e-04 |
| Outside | 8.402 e-05 | 1.985 e-05 |

## **Section D: S****ediment nutrient metrics**

Cores collected from inside and outside the farm in 2021 were analyzed at 0.5 cm intervals to a depth of 10 cm, and 1 cm intervals to a mean maximum depth of 20 cm. Plots of TOC%, TC% and TN% respectively can be found inSupplementary Figures D1-3, and tabulated nutrient data in Supplementary Tables D1-3. For the OLS conducted on the acidified dataset, the overall model for the effect of site type and depth on TOC% was significant (F(2, 33) = 3.83, p = 0.032, adj. R^2^ = 0.14). Within the model, site type was non-significant (p = 0.791), but depth was significant (p = 0.009, beta = -0.06, std. beta = -0.43). For the non-acidified dataset, the overall TC% model was non-significant (F(2, 33) = 2.52, p = 0.096, adj.R^2^ = 0.08), as were type (p = 0.101) and depth (p = 0.149) within the model. For TN%, the overall model was significant (F(2, 33) = 3.35, p = 0.047, adj. R^2^ = 0.12). Within the model, site type was non-significant (p = 0.752), but depth was significant (p = 0.015, beta < -0.01, std. beta = -0.41).

Integrated across the depths sampled for eDNA (i.e. 6.5 cm), Kruskal-Wallis tests showed no statistically significant differences for TOC% (Kruskal-Wallis chi-squared (KW χ^2^) = 0.0476, df = 1, p = 0.827), TC% (KW χ^2^ = 2.333, df = 1, p = 0.127) and TN% (KW χ^2^ = 0.429, df = 1, p = 0.513) between inside and outside samples. Likewise, integrated across the depths of the entire core which were sampled for nutrients, there were no statistically significant differences for TOC% (KW χ^2^ = 1.191, df = 1, p = 0.275), TN% (acid; KW χ^2^ = 0.0476, df = 1, p = 0.827) and TC% (KW χ^2^ = 0.429, df = 1, p = 0.513). However, TN% (KW χ^2^ = 3.857, df = 1, p = 0.0495) was statistically significant, if borderline, with TN% being greater in samples from inside the farm. Plots and tables of the integrated nutrient values have been omitted for brevity.

For the 2022 samples, TC% and TN% were measured at 1 cm intervals for the top 3 cm of each core. Plots of TC% and TN% can be found in Supplementary Figures D4-5 and tabulated nutrient data in Supplementary Tables D4-5. For all 2022 datasets, site number was nested within site type.

For TC%, the overall OLS model was statistically significant (F(5, 39) = 5.23, p < 0.001, adj. R^2^ = 0.32), but only depth was significant (p < 0.001, beta = -0.12, adj. beta = -0.57). Site type was non-significant (p = 0.091), as were any of the sites nested within site type.

For TN%, the overall OLS model was statistically significant (F(5, 39) = 4.43, p =0.003, adj. R^2^ = 0.28). Unlike TC%, both depth (p < 0.001, beta = -0.02, std. beta = -0.46) and site type (p = 0.036, beta = -0.02, std. beta = -0.87; using site type = inside as the baseline) were significant. None of the sites nested within site type were statistically significant.

Integrated across the sampled depths of the entire core, Kruskal-Wallis tests showed no statistically significant differences for TC% (KW χ^2^ = 1.694, df = 1, p = 0.193), TN% (KW χ^2^ = 2.647, df = 1, p = 0.104), d^13^C (KW χ^2^ = 0.953, df = 1, p = 0.329) and d^15^N (KW χ^2^ = 0.576, df = 1, p = 0.448) between inside and outside samples.

In both years, d^13^C and d^15^N showed no site-related differences between inside and outside the farm for both acidified and non-acidified datasets, and for both the full depth of the core and depths sampled for eDNA in the integrated datasets. Results have been omitted for brevity, including depth-related trends.

Overall, there was no clear difference in sediment carbon and nitrogen content between sites inside and outside the farm in both years of the study. The only nutrient metrics that showed a statistically significant effect of site type were the integrated TN% value for the entire cores in 2021, and TN% in 2022.


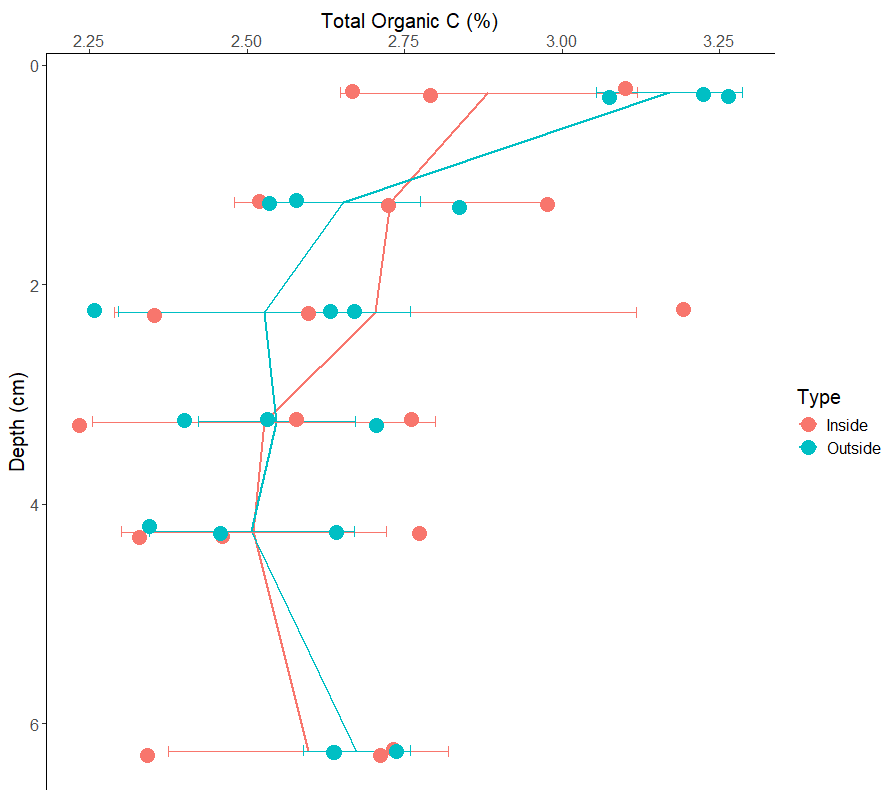


**Supplementary Figure D1:** Total organic carbon (TOC) % by depth for the 2021 sites, for the depths that were also sampled for eDNA. In 2021, 2 sites were sampled, 1 inside and 1 outside the farm, with 3 replicate cores collected per site. The colored lines represent mean values at each depth sampled, colored by site type. The horizontal bars with whiskers represent standard deviation at each depth. Depth values presented correspond to the middle of each depth horizon (e.g., the points at 6.25 cm correspond to the 6.0-6.5 cm depth horizon)

**Supplementary Table D1:** Summarized tabulated data for the 2021 total organic carbon (TOC%) plot in Supplementary Figure D1.

| Site Type | Depth (cm) | TOC% | S.D. |
| --- | --- | --- | --- |
| Inside | Aggregated | 2.658 | 0.267 |
| Inside | 0.25 | 2.883 | 0.236 |
| Inside | 1.25 | 2.727 | 0.247 |
| Inside | 2.25 | 2.703 | 0.414 |
| Inside | 3.25 | 2.527 | 0.272 |
| Inside | 4.25 | 2.510 | 0.211 |
| Inside | 6.25 | 2.597 | 0.223 |
| Outside | Aggregated | 2.679 | 0.266 |
| Outside | 0.25 | 3.170 | 0.115 |
| Outside | 1.25 | 2.653 | 0.121 |
| Outside | 2.25 | 2.527 | 0.232 |
| Outside | 3.25 | 2.547 | 0.125 |
| Outside | 4.25 | 2.510 | 0.163 |
| Outside | 6.25 | 2.673 | 0.085 |


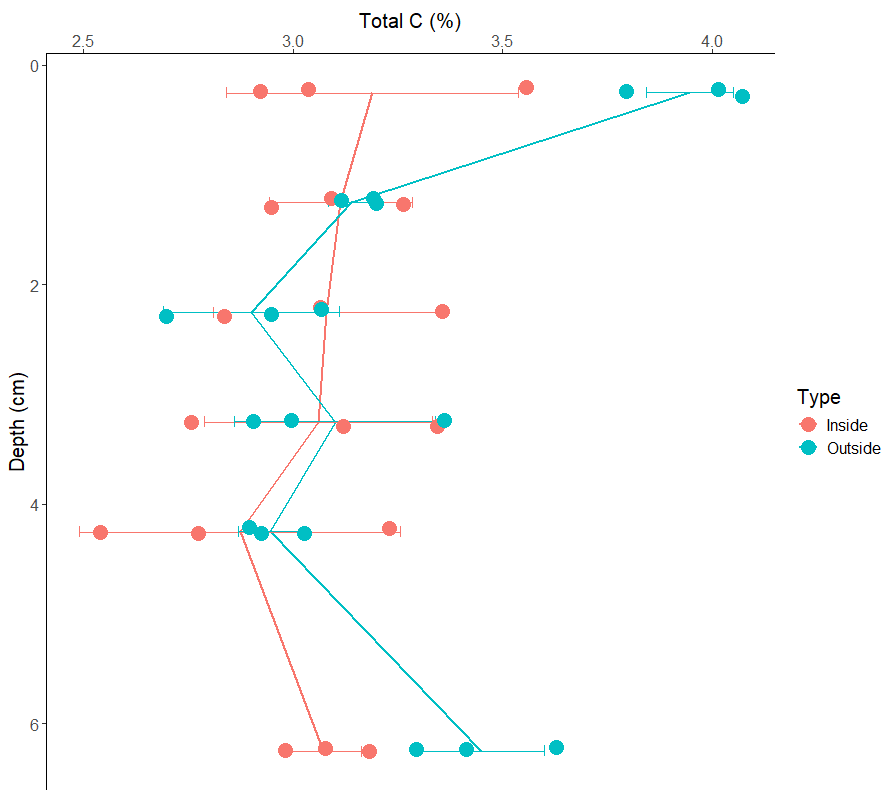


**Supplementary Figure D2**: Total carbon (TC) % by depth for the 2021 sites, for the depths that were also sampled for eDNA.

**Supplementary Table D2:** Summarized tabulated data for the 2021 total carbon (TC%) plot in Supplementary Figure D2.

| Site Type | Depth (cm) | TC% | S.D. |
| --- | --- | --- | --- |
| Inside | Aggregated | 3.065 | 0.251 |
| Inside | 0.25 | 3.190 | 0.348 |
| Inside | 1.25 | 3.113 | 0.170 |
| Inside | 2.25 | 3.080 | 0.271 |
| Inside | 3.25 | 3.060 | 0.272 |
| Inside | 4.25 | 2.873 | 0.383 |
| Inside | 6.25 | 3.073 | 0.089 |
| Outside | Aggregated | 3.247 | 0.392 |
| Outside | 0.25 | 3.947 | 0.104 |
| Outside | 1.25 | 3.140 | 0.056 |
| Outside | 2.25 | 2.900 | 0.210 |
| Outside | 3.25 | 3.100 | 0.240 |
| Outside | 4.25 | 2.947 | 0.078 |
| Outside | 6.25 | 3.450 | 0.149 |


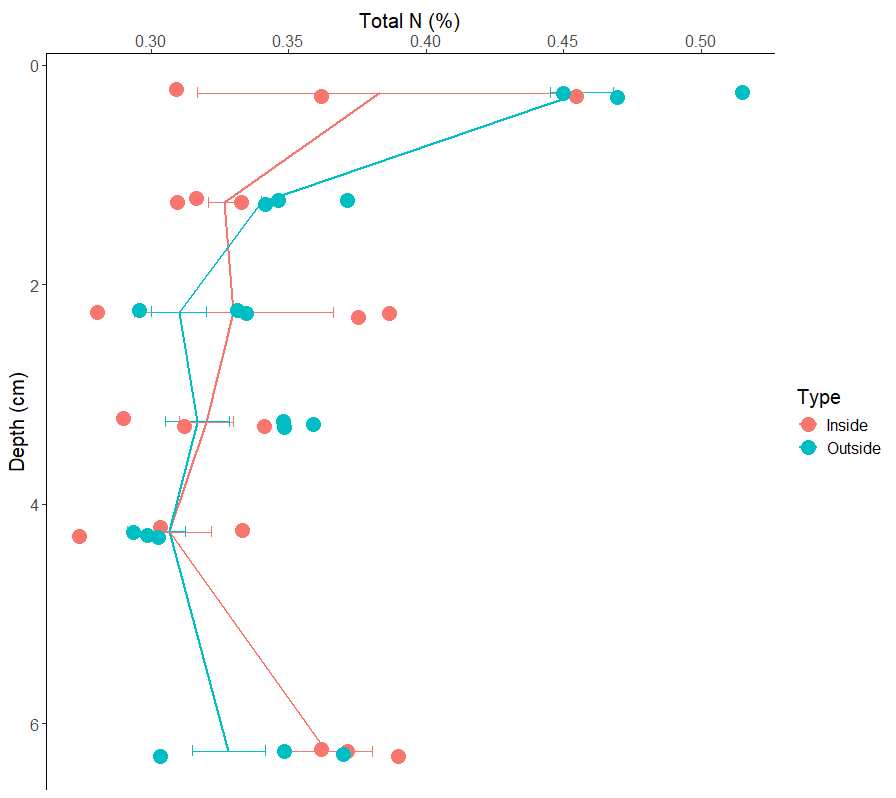


**Supplementary Figure D3**: Total nitrogen (TN) % by depth for the 2021 sites, for the depths that were also sampled for eDNA.

**Supplementary Table D3:** Summarized tabulated data for the 2021 total nitrogen (TN%) plot in Supplementary Figure D3.

| Site Type | Depth (cm) | TN% | S.D. |
| --- | --- | --- | --- |
| Inside | Aggregated | 0.338 | 0.039 |
| Inside | 0.25 | 0.383 | 0.067 |
| Inside | 1.25 | 0.327 | 0.006 |
| Inside | 2.25 | 0.330 | 0.036 |
| Inside | 3.25 | 0.320 | 0.010 |
| Inside | 4.25 | 0.307 | 0.015 |
| Inside | 6.25 | 0.364 | 0.017 |
| Outside | Aggregated | 0.343 | 0.054 |
| Outside | 0.25 | 0.457 | 0.012 |
| Outside | 1.25 | 0.340 | <0.001 |
| Outside | 2.25 | 0.310 | 0.010 |
| Outside | 3.25 | 0.317 | 0.012 |
| Outside | 4.25 | 0.307 | 0.006 |
| Outside | 6.25 | 0.328 | 0.013 |


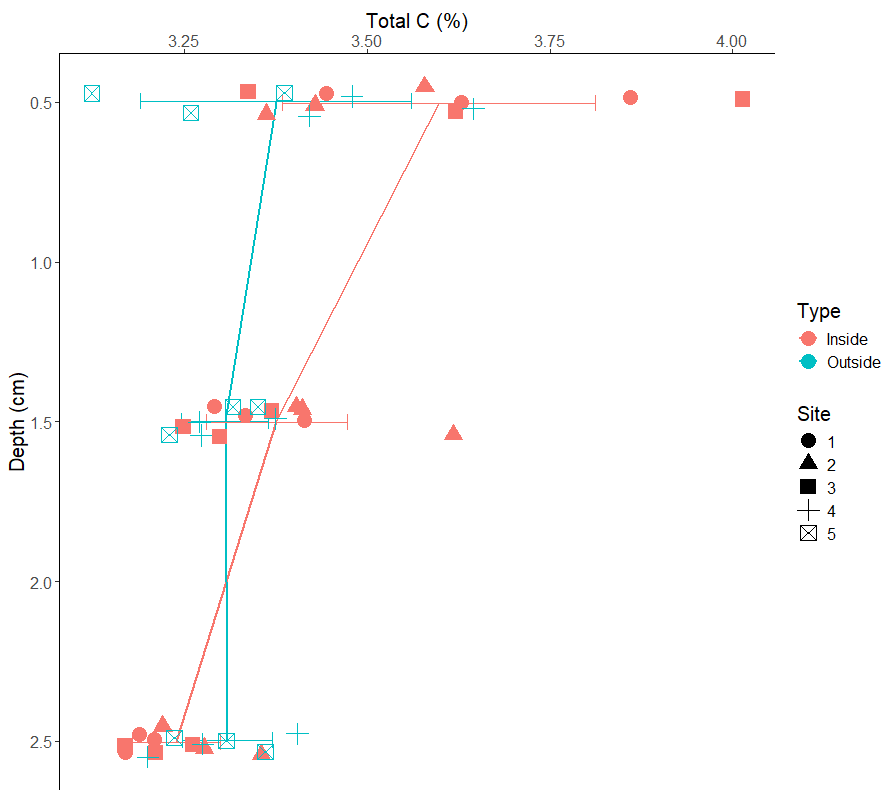


**Supplementary Figure D4**: Total carbon (TC) % by depth for the 2022 sites. In 2022, five sites were sampled, three inside and two outside the farm, with three replicate cores collected per site. The same interpretation of the plot applies as in Supplementary Figure D1, with the addition of shape to demarcate site number.

**Supplementary Table D4:** Summarized tabulated data for the 2022 total carbon (TC%) plot in Supplementary Figure D4.

| Site Type | Depth (cm) | TC% | S.D. |
| --- | --- | --- | --- |
| Inside | Aggregated | 3.404 | 0.202 |
| Inside | 0.5 | 3.598 | 0.214 |
| Inside | 1.5 | 3.376 | 0.097 |
| Inside | 2.5 | 3.239 | 0.060 |
| Outside | Aggregated | 3.330 | 0.019 |
| Outside | 0.5 | 3.375 | 0.186 |
| Outside | 1.5 | 3.305 | 0.059 |
| Outside | 2.5 | 3.309 | 0.061 |
| Site 1 | Aggregated | 3.400 | 0.225 |
| Site 2 | Aggregated | 3.418 | 0.133 |
| Site 3 | Aggregated | 3.394 | 0.253 |
| Site 4 | Aggregated | 3.377 | 0.136 |
| Site 5 | Aggregated | 3.283 | 0.070 |


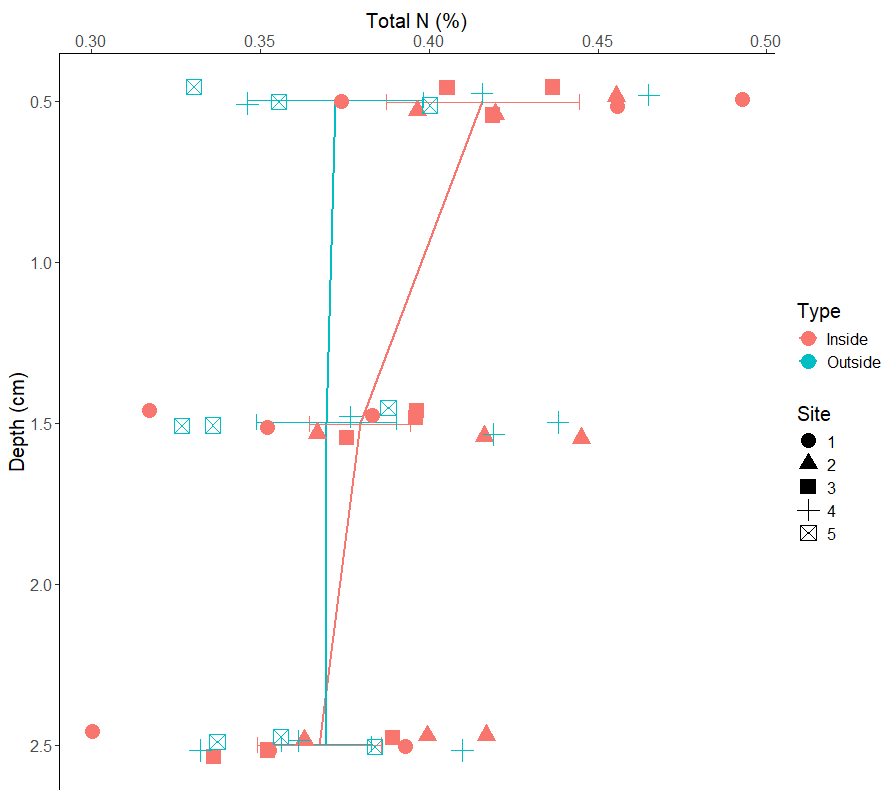


**Supplementary Figure D5:** Total nitrogen (TN) % by depth for the 2022 sites.

**Supplementary Table D5:** Summarized tabulated data for the 2022 total nitrogen (TN%) plot in Supplementary Figure D5.

| Site Type/Number | Depth (cm) | TN% | S.D. |
| --- | --- | --- | --- |
| Inside | Aggregated | 0.388 | 0.029 |
| Inside | 0.5 | 0.416 | 0.029 |
| Inside | 1.5 | 0.380 | 0.015 |
| Inside | 2.5 | 0.367 | 0.018 |
| Outside | Aggregated | 0.371 | 0.019 |
| Outside | 0.5 | 0.372 | 0.026 |
| Outside | 1.5 | 0.370 | 0.021 |
| Outside | 2.5 | 0.370 | 0.013 |
| Site 1 | Aggregated | 0.385 | 0.036 |
| Site 2 | Aggregated | 0.390 | 0.014 |
| Site 3 | Aggregated | 0.388 | 0.036 |
| Site 4 | Aggregated | 0.380 | 0.019 |
| Site 5 | Aggregated | 0.361 | 0.016 |

###

## **Section E:** **2021 16S + 18S metabarcoding data**

**Alpha diversity metrics: 16S and 18S**

Based on initial taxonomic barplots, suspected contaminant taxa from seawater (of the genus *Aquimarina* & family *Vibrionaceae*) were present in the top three layers analyzed in this dataset (0.25cm, 0.75cm, 1.25cm). We believe these contaminant taxa to originate from unfiltered seawater used for the sediment core holding tanks prior to processing, which may have been entrained into the surficial sediment layers during processing. Such taxa were present in high abundance where present, but were highly inconsistent and variable in relative abundance across cores, and not known to be part of the sediment microbiome. As such, these taxa were removed prior to all downstream analyses. The major taxa in the 2021 core microbiome post-removal of contaminant taxa showed relative similarity to the 2022 core microbiome.

Firstly, basic alpha diversity metrics were calculated for the metabarcoding datasets to examine whether the number of ASVs (and by extension, taxa) differs between samples inside and outside the farm, as well as with depth and COI gene copy number. Violin plots of observed diversity (OD) and the Shannon Index (SI) by site type and depth can be found in Supplementary Figures E1-2 (16S) and E3-4 (18S). Observed diversity in this case refers to the absolute number of ASVs, while the Shannon index also accounts for the abundance of each ASV. Tabulated OD and SI values by site type and depth can be found in Supplementary Tables E1 and E2.

For the 2021 16S dataset, Kruskal-Wallis tests showed no statistically significant differences between samples from inside and outside the farm, for both OD (KW χ2 = 0.499, df = 1, p = 0.480) and the SI (KW χ2 = 0.107, df = 1, p = 0.744).

Likewise, for the 2021 18S dataset, Kruskal-Wallis tests showed no statistically significant differences between samples from inside and outside the farm, for both OD (KW χ2 = 0.881, df = 1, p = 0.348) and SI (KW χ2 = 1.602, df = 1, p = 0.206).

For COI gene copy number, there was no statistically significant association in both datasets, for both indices (16S-OD: KW χ2 = 26.859, df = 29, p = 0.579; 16S-SI: KW χ2 = 26.178, df = 29, p = 0.616; 18S-OD: KW χ2 = 28, df = 28, p = 0.464; 18S-SI: KW χ2 = 28, df = 28, p = 0.464).

Depth was not statistically significant for OD (KW χ2 = 6.736, df = 4, p = 0.151) and SI (KW χ2 = 7.603, df = 4, p = 0.107) in the 16S dataset. On the other hand, depth was statistically significant for both OD (KW χ2 = 10.707, df = 4, p = 0.0301) and SI (KW χ2 = 10.462, df = 4, p = 0.0333) in the 18S dataset. The post-hoc Dunn’s tests showed the only significant pairwise differences to be that between the 2.25 cm and 4.75 cm depth horizons for both OD (p = 0.0177) and SI (p = 0.0220), where both indices were greater at the latter depth than the former.


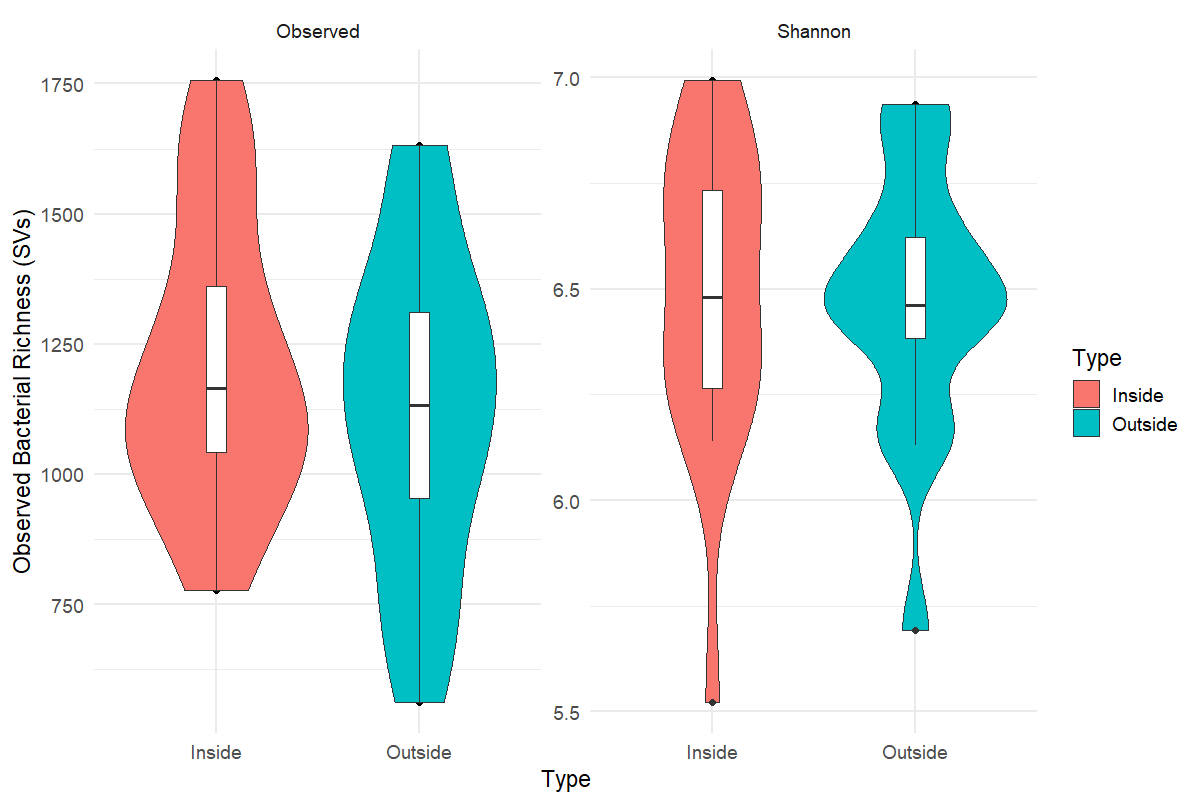


**Supplementary Figure E1:** Violin plots of alpha diversity (observed diversity and the Shannon Index) for the 2021 16S dataset, colored by site type.


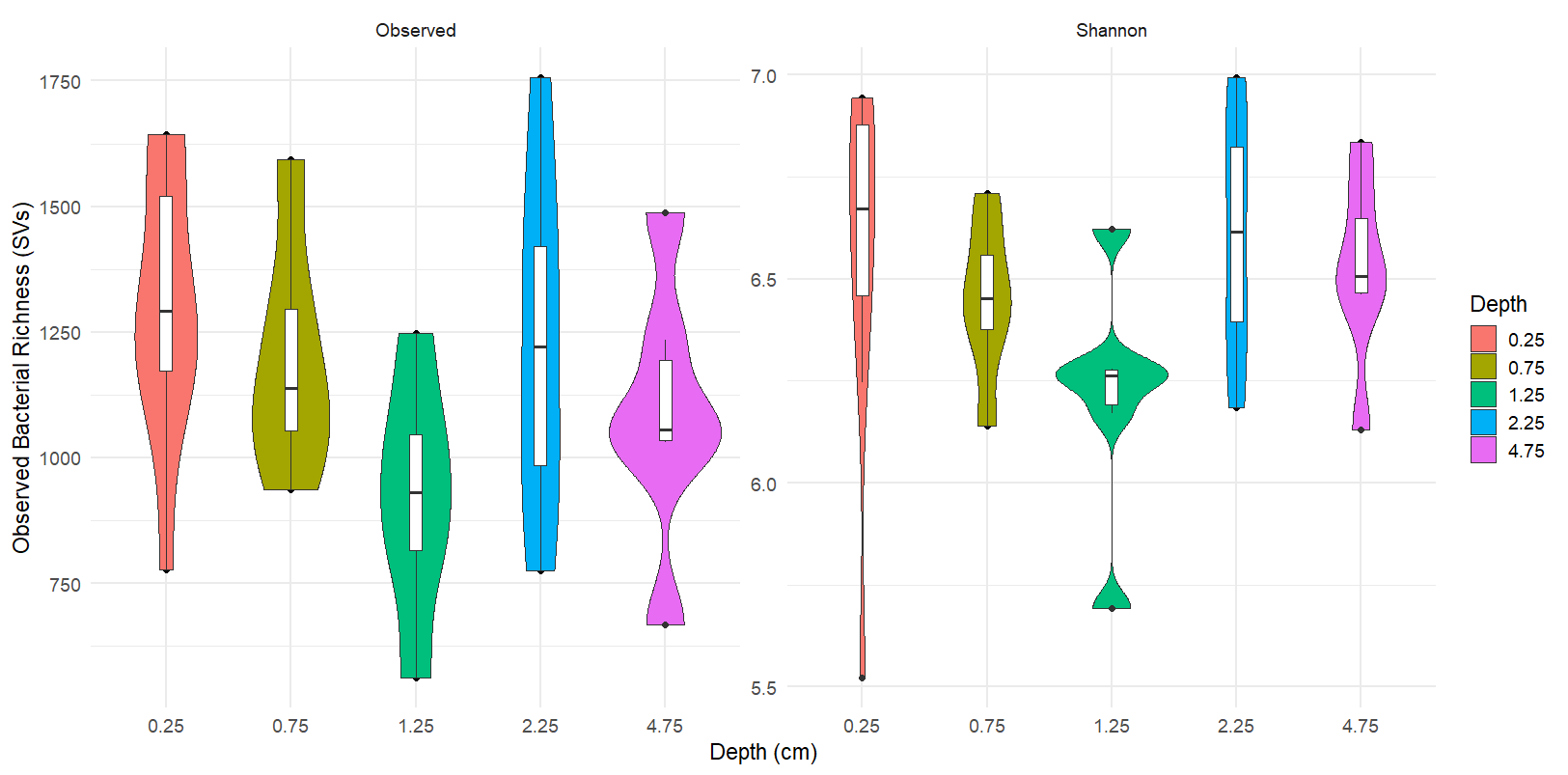


**Supplementary Figure E2:** Violin plots of alpha diversity (observed diversity and the Shannon Index) for the 2021 16S dataset, colored by sample depth. Site type has been omitted for simplicity.

**Supplementary Table E1:** Tabulated alpha diversity summary statistics (observed diversity and the Shannon Index) for the 2021 16S dataset, organized by site type and depth. Observed diversity values are given to 1 decimal place.

| Type | Depth (cm) | Observed Diversity | S.D. | Shannon Index | S.D. |
| --- | --- | --- | --- | --- | --- |
| Inside | 0.25 | 1217.8 | 310.9 | 6.420 | 0.562 |
| Inside | 0.75 | 1205.3 | 344.6 | 6.401 | 0.289 |
| Inside | 1.25 | 975.3 | 95.9 | 6.267 | 0.011 |
| Inside | 2.25 | 1399.7 | 339.7 | 6.742 | 0.283 |
| Inside | 4.75 | 1255.0 | 223.7 | 6.667 | 0.178 |
| Outside | 0.25 | 1387.4 | 215.2 | 6.693 | 0.236 |
| Outside | 0.75 | 1188.3 | 143.0 | 6.498 | 0.082 |
| Outside | 1.25 | 869.7 | 347.7 | 6.162 | 0.465 |
| Outside | 2.25 | 1056.0 | 344.3 | 6.467 | 0.333 |
| Outside | 4.75 | 922.3 | 221.9 | 6.375 | 0.215 |

**
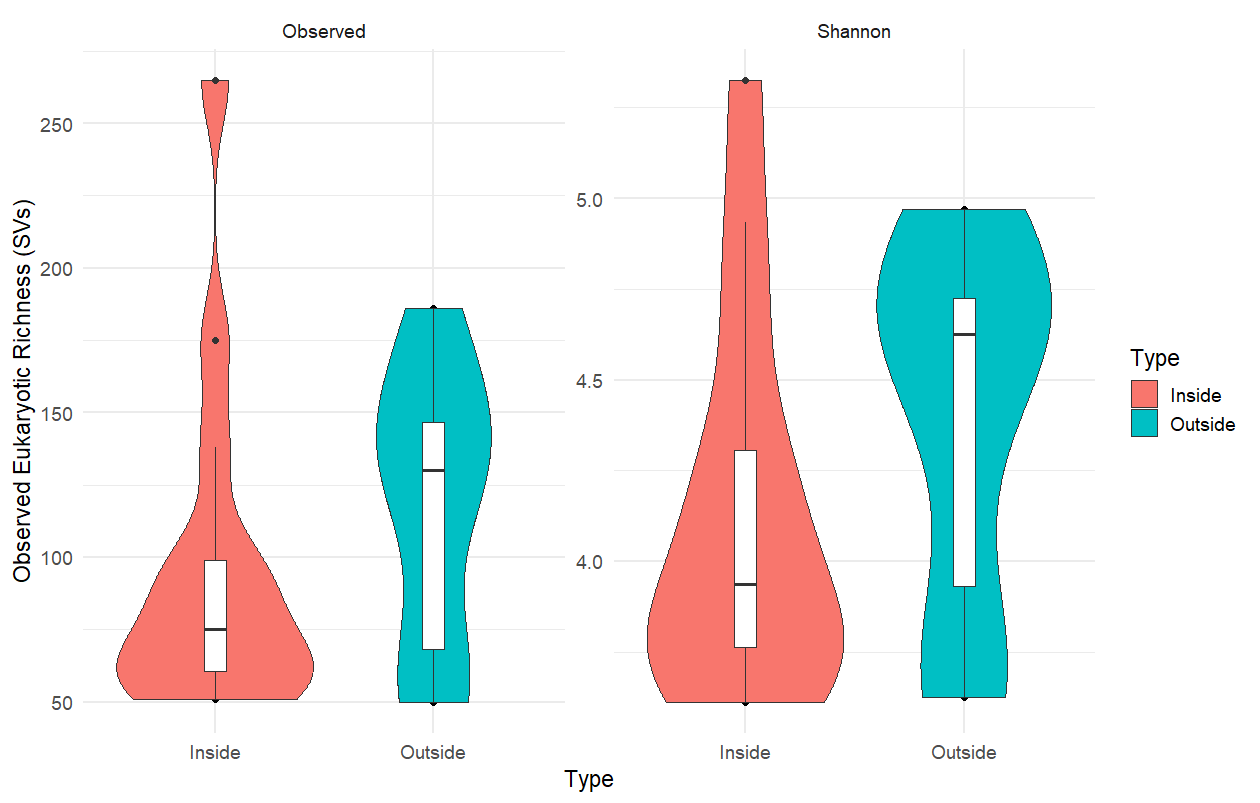
**

**Supplementary Figure E3:** Violin plots of alpha diversity (observed diversity and the Shannon Index) for the 2021 18S dataset, colored by site type.

**
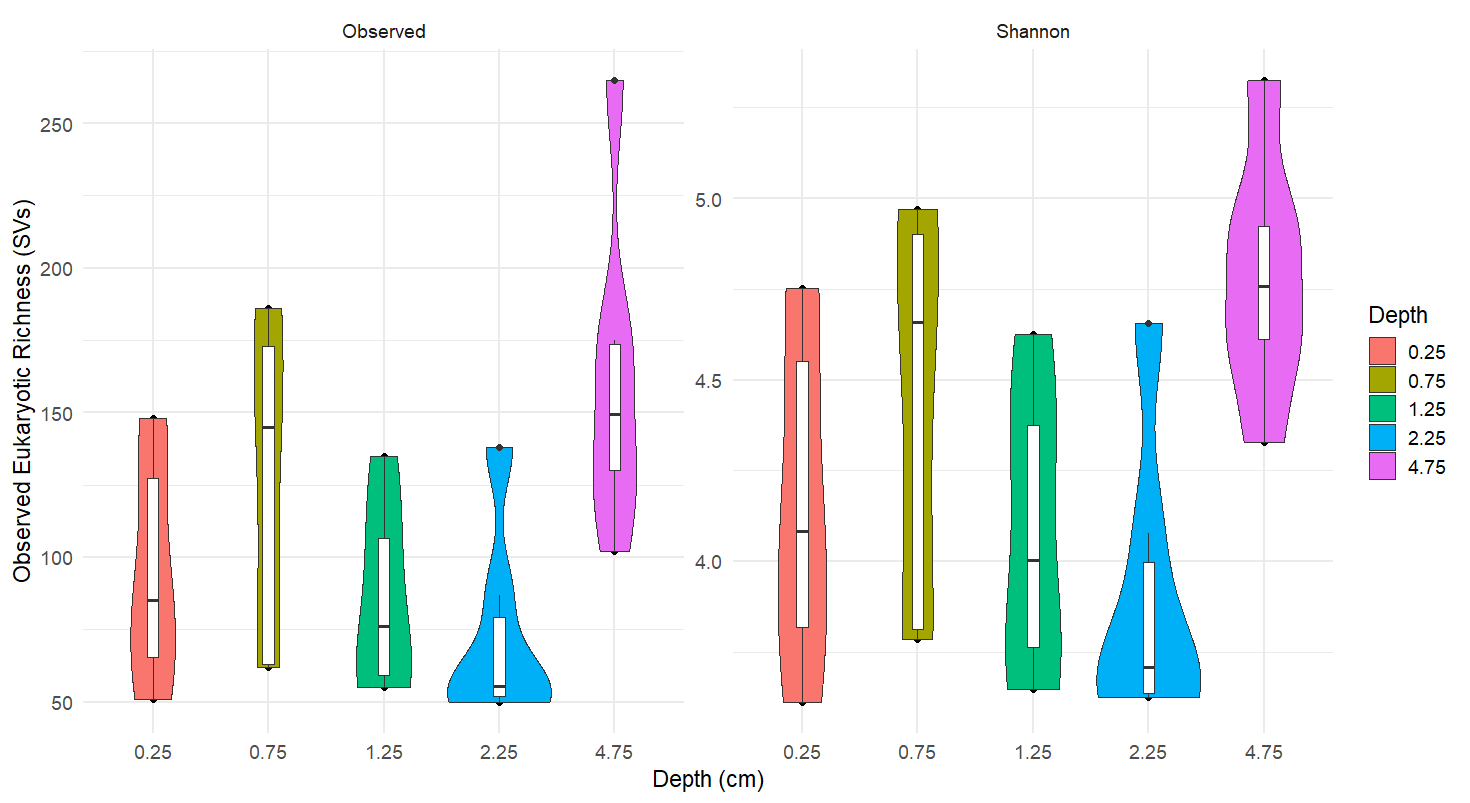
**

**Supplementary Figure E4:** Violin plots of alpha diversity (observed diversity and the Shannon Index) for the 2021 18S dataset, colored by sample depth. Site type has been omitted for simplicity.

**Supplementary Table E2:** Tabulated alpha diversity summary statistics (observed diversity and the Shannon Index) for the 2021 18S dataset, organized by site type and depth. Observed diversity values are given to 1 decimal place.

| Type | Depth (cm) | Observed Diversity | S.D. | Shannon Index | S.D. |
| --- | --- | --- | --- | --- | --- |
| Inside | 0.25 | 66.7 | 19.9 | 3.804 | 0.233 |
| Inside | 0.75 | 62.5 | 0.7 | 3.798 | 0.019 |
| Inside | 1.25 | 70.0 | 17.4 | 3.921 | 0.271 |
| Inside | 2.25 | 93.7 | 41.4 | 4.120 | 0.515 |
| Inside | 4.75 | 180.7 | 81.6 | 4.863 | 0.503 |
| Outside | 0.25 | 123.0 | 36.6 | 4.518 | 0.362 |
| Outside | 0.75 | 168.0 | 21.0 | 4.844 | 0.163 |
| Outside | 1.25 | 100.7 | 41.2 | 4.231 | 0.517 |
| Outside | 2.25 | 52.0 | 2.6 | 3.681 | 0.069 |
| Outside | 4.75 | 143.0 | 22.5 | 4.707 | 0.155 |

**Beta diversity: 16S**

Analysis of 16S beta diversity indicated significant differences in the Shannon index with depth (but not observed diversity), with some shallower depth horizons showing a higher Shannon index than deeper depth horizons. Thus, further analyses were conducted to examine subtler variations in taxonomic distribution with site and depth, and other covariates of interest, such as COI gene copy number.

Firstly, family-level barplots of the top 30 microbial taxa were made, grouped by depth and then site type (Supplementary Figure E5). At a glance, depth-related differences in community composition seem to be more prominent than any potential site-type related differences. In general, common families in the core microbiome, regardless of site type and depth, include *Flavobacteriaceae* (e.g., the genera *Lutibacter, Eudorea, Winogradskyella*)*, Desulfosarcinaceae, Pirellulaceae, Woeseiaceae, Thermoanaerobaculaceae* and *Marinfilaceae* (e.g., the genus *Marinifilum*)*.*


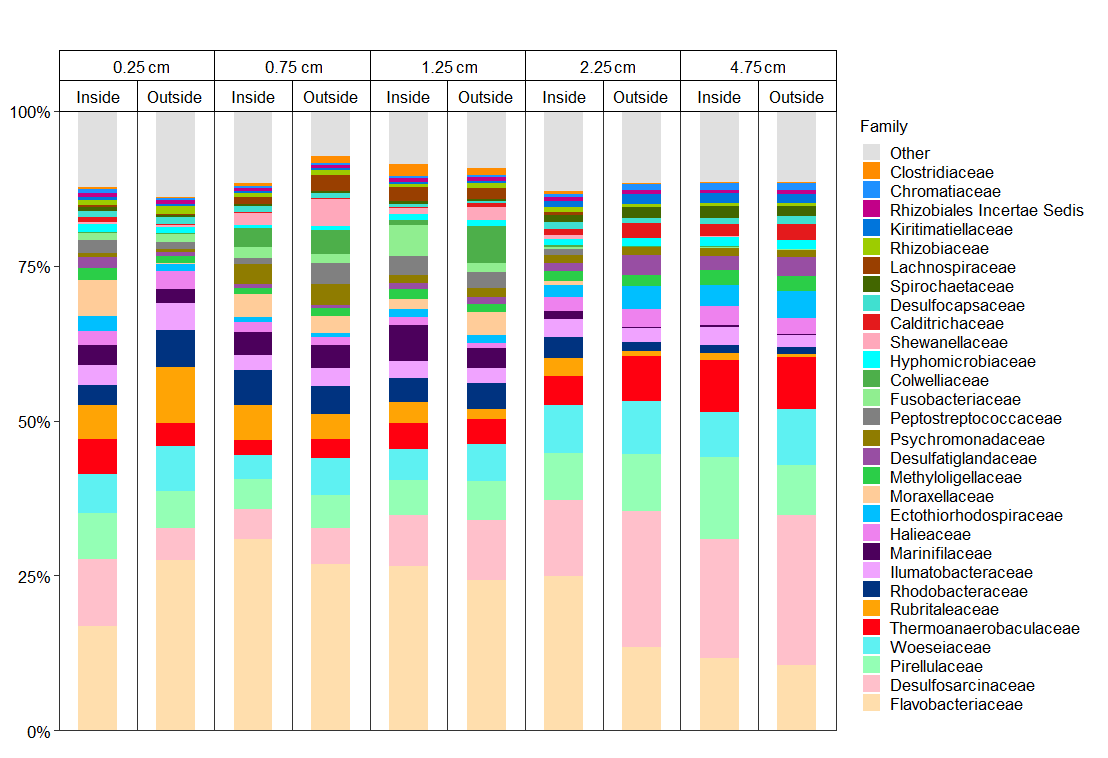


**Supplementary Figure E5:** Stacked bar plots of the top 30 microbial families in the 2021 16S dataset by relative abundance, grouped together by sample depth (with the value representing the mid-depth of each depth horizon), and then by site type. Remaining taxa have been aggregated into an “Other” category.

A Principal Coordinates Analysis (PCoA) of the 16S dataset was used to examine dissimilarity in community composition between the different samples, based on Bray-Curtis dissimilarity. Two variants of the PCoA were made, one colored by sample depth (Supplementary Figure E6), and the other colored by log COI gene copy number (Supplementary Figure E7). Visually, there was no clear separation between samples from inside and outside the farm in PCoA space, but some depth-related clustering was observed. Samples with relatively high *S. latissima* COI gene copy numbers did not appear to cluster separately from other samples from the same depth horizons, regardless of site type.


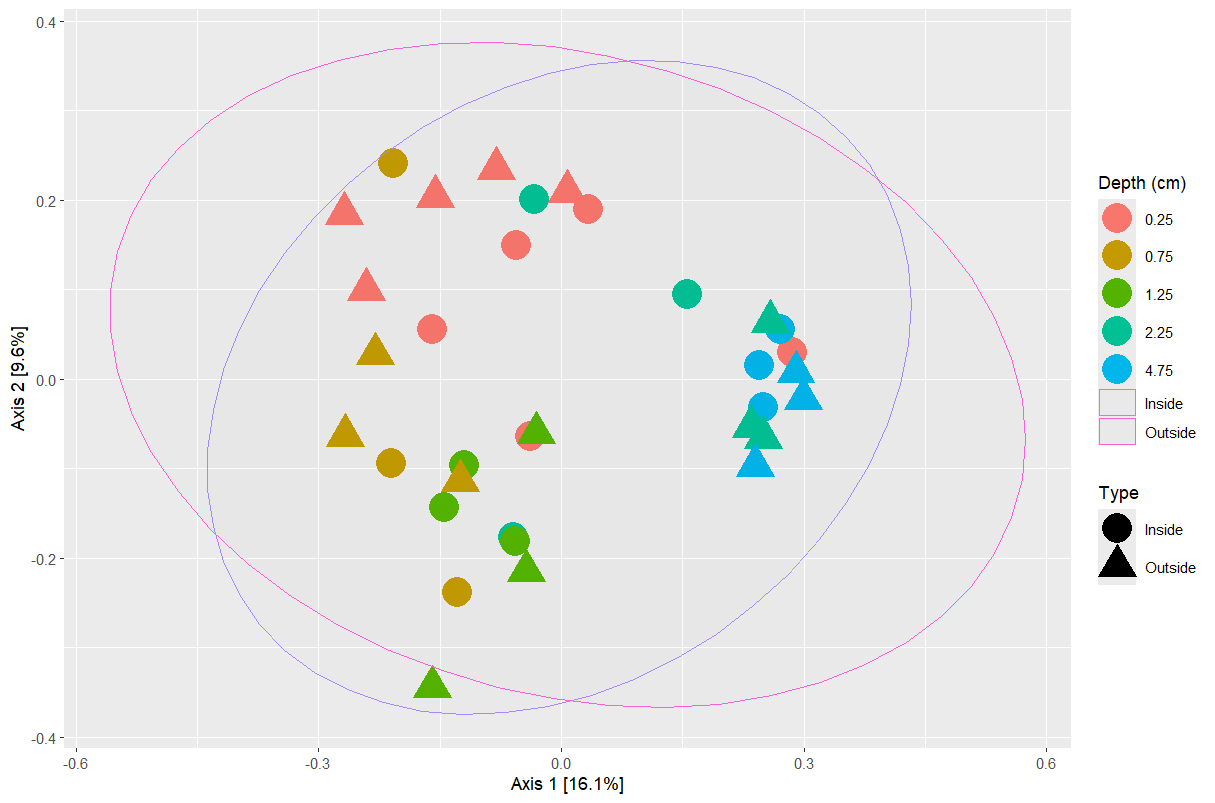


**Supplementary Figure E6:** Principal coordinates analysis (PCoA) plot of the 2021 16S microbiome dataset, based on Bray-Curtis distances. Each point represents a single sample, with shape representing site type (inside vs outside the farm) and color representing the depth of the sample. Ellipses have been drawn around points of the two site types at the 95% confidence interval; with the lines colored by type.


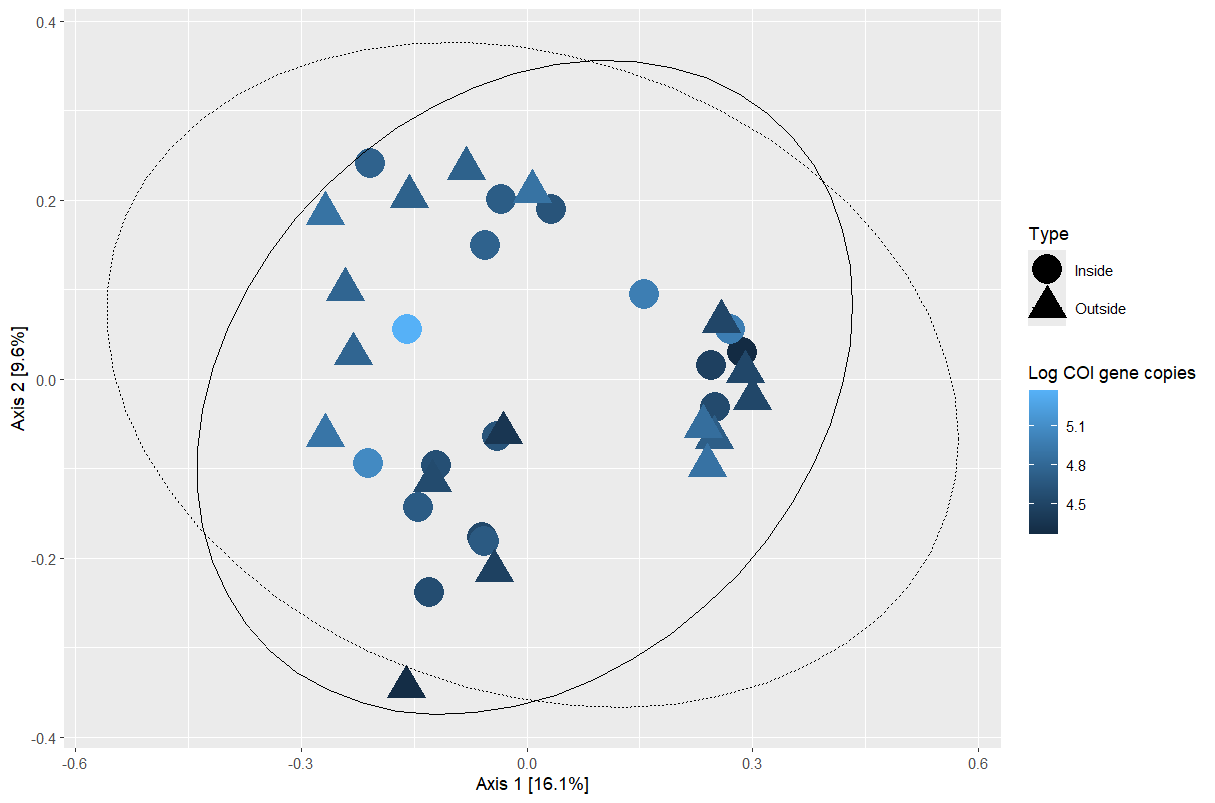


**Supplementary Figure E7:** Alternative principal coordinates analysis (PCoA) plot of the 2021 16S microbiome dataset, based on Bray-Curtis distances. Each point represents a single sample, with shape representing site type (inside vs outside the farm) and color representing log *S. latissima* COI gene copies, with lighter colors representing greater values. Ellipses have been drawn around points of the two site types at the 95% confidence interval; with the ellipse with solid lines representing samples from inside the farm, and the ellipse with dotted lines representing samples from outside the farm.

A PERMANOVA was then conducted to examine factors of interest (i.e. site type, depth, COI, nutrients) that might show statistically significant associations with the Bray-Curtis distances between samples. Sample depth (p = 0.0001, ⍵2 = 0.0874) was the only statistically significant factor structuring the microbial communities in the samples, while site type (p = 0.380) and *S. latissima* COI gene copy number (p = 0.159) were non-significant.

A second PERMANOVA was also conducted on a subset of the dataset with full nutrient data, which found site type (p = 0.555), TOC% (p =0.388), TC% (p = 0.810) and log COI (pm = 0.0810) to be non-significant. Sample depth (p = 0.0001, ⍵2 = 0.0654) was once again statistically significant, but this time, so was TN% (p = 0.0148, ⍵2 = 0.0328). Based on the PERMANOVA effect sizes, sample depth is more important in structuring 16S community composition than TN%. This can also be seen in the PCoA, where there is some visual separation based on sample depth, rather than site type. Regardless, the amount of variance explained on both principal coordinate axes is relatively low.

A family-level volcano plot ([Supplementary](#7ni7y5x0vvfe) Figure E8) was made to examine potential indicator taxa that may be associated with sediment from the two different site types. Using a p-value threshold of 0.05 (a log value of ~1.3) as the cutoff, the only relatively widespread taxon that is enriched in samples inside the farm relative to outside the farm is *Marinilabilaceae* (and to a lesser extent in terms of prevalence across all samples, *Desulfolunaceae*). Conversely, *Woeseiaceae* and *Acanthopleuribacteraceae* are relatively more enriched in samples from outside the farm. The lack of statistical significance of site type in the PERMANOVA analyses supports the relative dearth of significant potential indicator taxa based on site type.


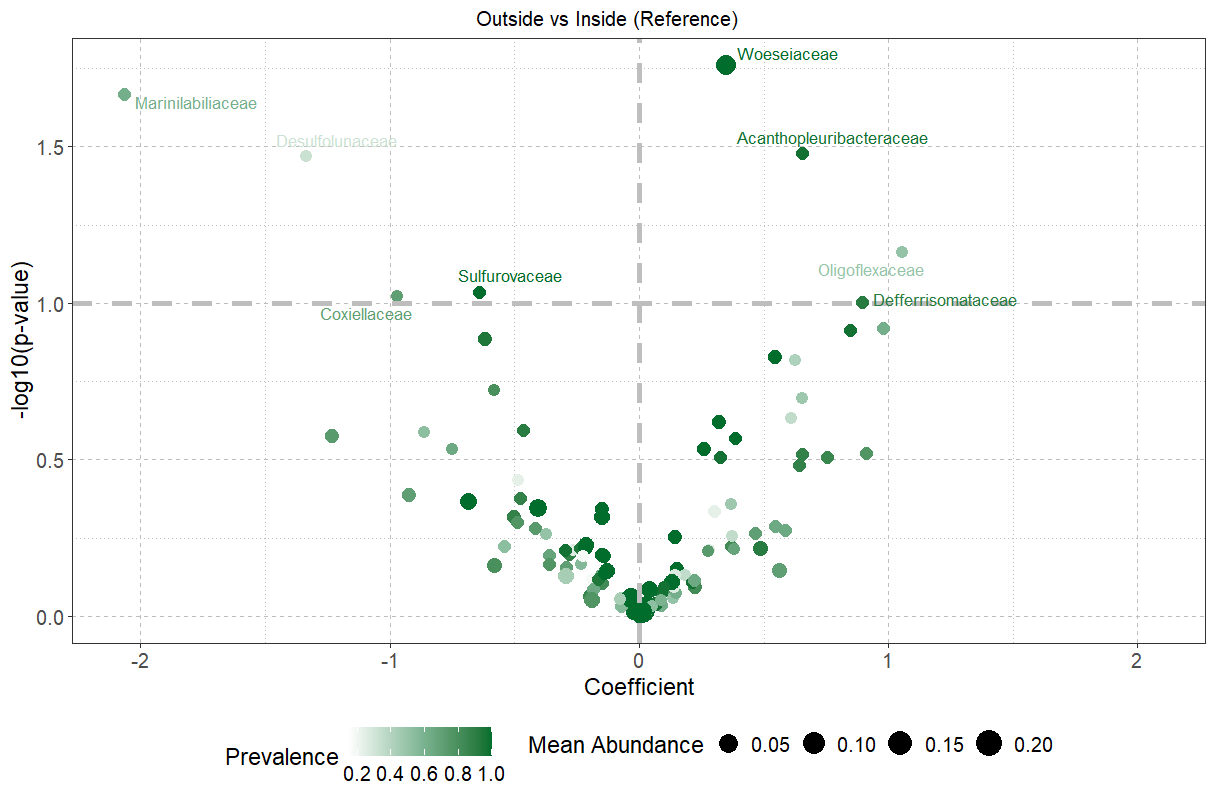


**Supplementary Figure E8:** Volcano plot of potential microbial indicator taxa at the family level distinguishing samples from inside and outside the farm in the 2021 16S dataset. Potential indicator taxa are determined by differential log-fold changes, with a negative coefficient on the x-axis representing a relative enrichment in samples from inside the farm, and vice versa. The size of the dot for each taxon represents its mean abundance in samples across the dataset where present, while the color intensity represents its prevalence across all samples in the dataset. The dotted gray line on the y-axis represents a cut-off p-value of 0.1 for statistically significant taxa. A more stringent p-value cut-off of 0.05 would be represented by a y-axis value of ~1.3. Likewise, a p-value cut-off of 0.01 would be presented by a y-axis value of 2.

To examine indicator taxa in further depth, a correlation analysis (Supplementary Table E3) was conducted to examine taxa that showed statistically significant correlations with S. latissima COI gene copy number, and by extension, possibly kelp biomass presence and quantity. Prominent taxa associated with sediments from inside the farm that were positively correlated with COI values *include Sphingomonadaceae, Schleiferiaceae, Gimesiaceae, Pseudoalteromonadaceae, Granulosicoccaceae* and *Rhodobacteraceae.* There were no clear overlaps between the indicator taxa provided by the correlation analysis and the volcano plots.

**Supplementary Table E3:** A list of the top significant taxa (p < 0.05) in the correlation analysis for the 2021 16S dataset, and their associated p-values, Spearman’s rho values and site types. Spearman's rho values range from -1 to +1, indicating negative to positive correlation respectively. Taxa are correlated with both site type and log COI values, and are sorted by site type, then p-value, and then finally rho.

| **Type** | **Family** | **rho** | **p-value** |
| --- | --- | --- | --- |
| Inside | *Sphingomonadaceae* | 0.661601 | 0.003821 |
| Inside | *Schleiferiaceae* | 0.613351 | 0.008833 |
| Inside | AB-539-J10 | -0.5887 | 0.012911 |
| Inside | *Gimesiaceae* | 0.572013 | 0.016429 |
| Inside | *Pseudoalteromonadaceae* | 0.556418 | 0.020358 |
| Inside | *Granulosicoccaceae* | 0.517813 | 0.033248 |
| Inside | *Rhodobacteraceae* | 0.511657 | 0.035779 |
| Inside | *Anaerolineaceae* | -0.49571 | 0.043018 |
| Inside | *Chitinophagaceae* | 0.482801 | 0.049644 |
| Outside | *Methylophagaceae* | 0.715433 | 0.001243 |
| Outside | *Spirosomaceae* | 0.689135 | 0.002214 |
| Outside | *Oscillospiraceae* | -0.65948 | 0.003976 |
| Outside | *Blastocatellaceae* | 0.636368 | 0.006024 |
| Outside | *Phycisphaeraceae* | 0.603194 | 0.010366 |
| Outside | *Bacteriovoracaceae* | 0.599145 | 0.011033 |
| Outside | *Saprospiraceae* | 0.583026 | 0.014033 |
| Outside | *Moritellaceae* | 0.559924 | 0.019417 |
| Outside | *Fokiniaceae* | 0.557671 | 0.020018 |
| Outside | *Rickettsiaceae* | 0.554694 | 0.020834 |
| Outside | *Izemoplasmataceae* | 0.536703 | 0.026333 |
| Outside | *Desulfocapsaceae* | 0.532516 | 0.02776 |
| Outside | AB-539-J10 | -0.51872 | 0.032886 |
| Outside | *Alteromonadaceae* | 0.51295 | 0.035235 |
| Outside | *Competibacteraceae* | 0.511964 | 0.035649 |
| Outside | *Spongiibacteraceae* | 0.498551 | 0.041652 |
| Outside | *Halomonadaceae* | -0.49633 | 0.042717 |
| Outside | *Gimesiaceae* | 0.495755 | 0.042994 |
| Outside | *Crocinitomicaceae* | 0.486455 | 0.047695 |
| Outside | *Rubritaleaceae* | 0.484663 | 0.048644 |
| Outside | *Pseudonocardiaceae* | 0.484128 | 0.04893 |

**Beta diversity: 18S**

Similar to the 16S dataset, the only trends in the 18S dataset with regards to alpha diversity metrics were depth-related, with both observed diversity and the Shannon Index being significantly different with depth.

Likewise, barplots of the the top 30 taxa (Supplementary Figure E9) (in this case, genera) show more dramatic differences in community composition with depth (e.g., greater relative abundance of *Alexandrium* and unclassified taxa with depth), but with greater heterogeneity between site types within each depth stratum, which may be due to the larger size and thus potentially greater eDNA heterogeneity of both microeukaryotes and macroeukaryotes (e.g., *Bulla* reads in 0.25cm sediments from inside the farm, which may correspond to a related gastropod). The 18S dataset largely consists of microeukaryotes, both planktonic and benthic, with a significant component also coming from the eelgrass *Zostera marina*, which is common in shallow subtidal waters in the region.


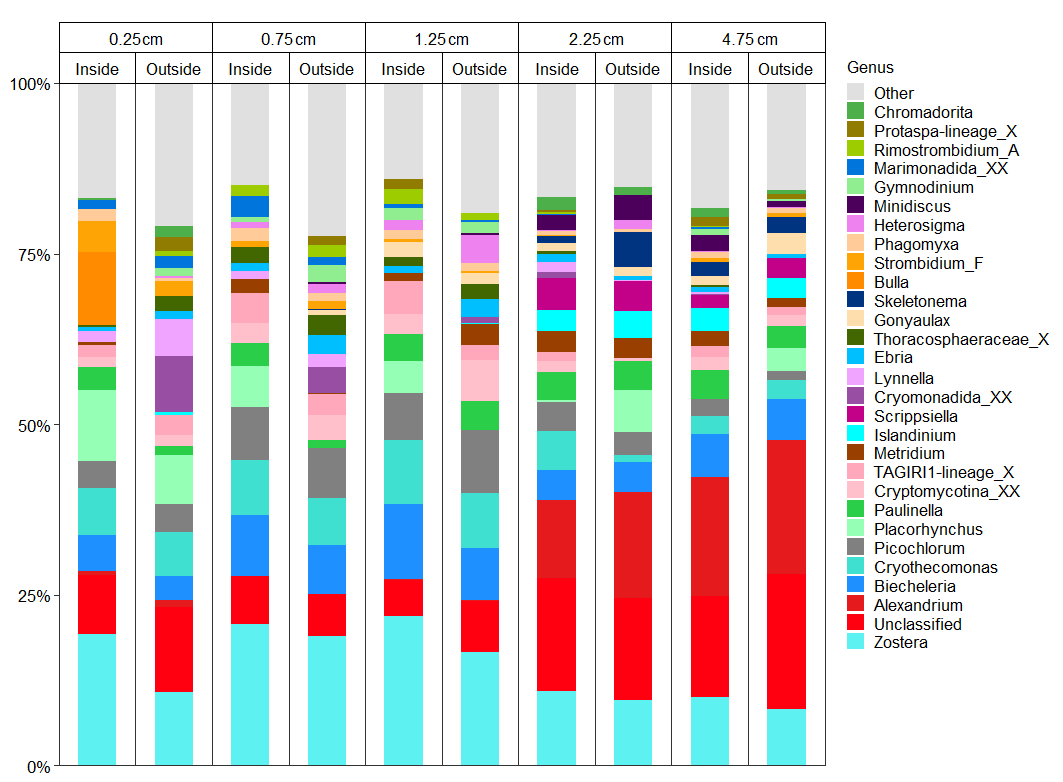


**Supplementary Figure E9:** Stacked bar plots of the top 30 eukaryotic genera in the 2021 18S dataset by relative abundance, grouped together by sample depth (with the value representing the mid-depth of each depth horizon), and then by site type. Remaining taxa have been aggregated into an “Other” category.

The main genera in the core eukaryotic community include *Zostera* and *Bierchelaria*, with the *Zostera* reads corresponding to many unique ASVs. Thus, the *Zostera* reads may possibly correspond to 18S eDNA from terrestrial angiosperms as well as *Zostera marina* itself. Alternatively, these variants may correspond to degraded eDNA from *Zostera marina*.

Similar to the 16S dataset, the PCoAs (colored by depth: Supplementary Figure E10; colored by log COI values: Supplementary Figure E11) showed stronger visual separation of samples by depth, with no obvious separation by site type, or COI gene copy number.


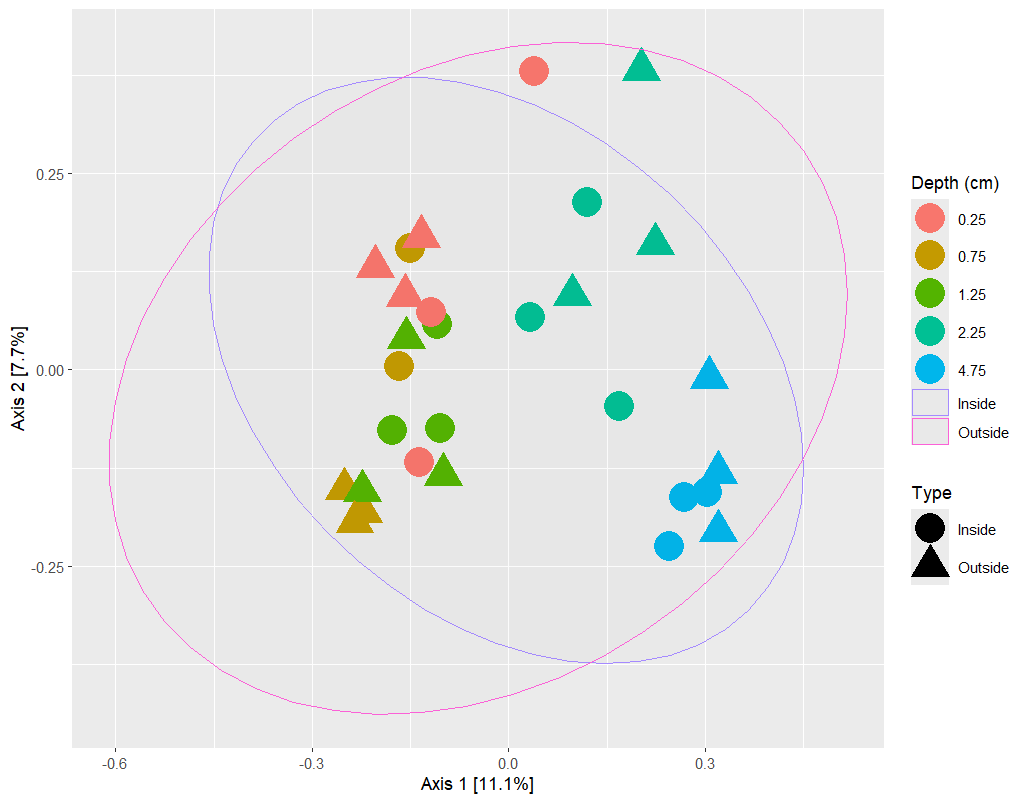


**Supplementary Figure E10:** Principal coordinates analysis (PCoA) plot of the 2021 18S eukaryotic dataset, based on Bray-Curtis distances. Ellipses have been drawn around points of the two site types at the 95% confidence interval; with the lines colored by type. Refer to Supplementary Figure E6 for further details on figure interpretation.


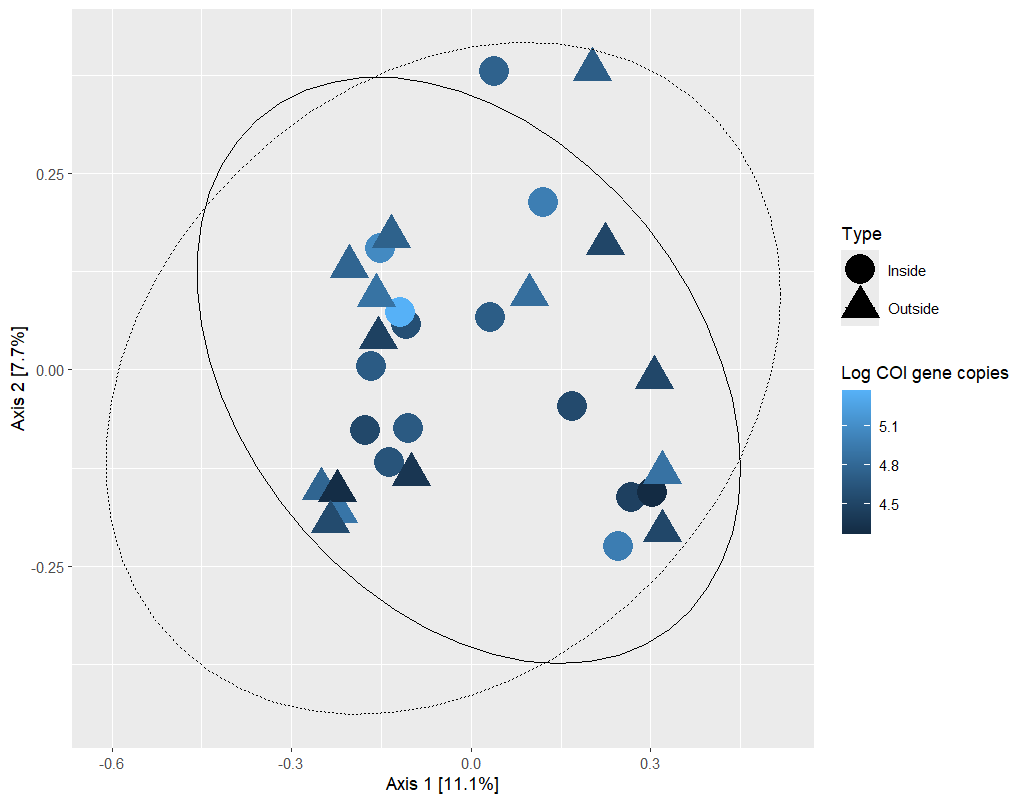


**Supplementary Figure E11:** Alternative principal coordinates analysis (PCoA) plot of the 2021 18S eukaryotic dataset, based on Bray-Curtis distances. The color intensity of each point represents log *S. latissima* COI gene copies, with lighter colors representing greater values. Ellipses have been drawn around points of the two site types at the 95% confidence interval; with the ellipse with solid lines representing samples from inside the farm, and the ellipse with dotted lines representing samples from outside the farm.

The PERMANOVA run on the full dataset showed only depth (p = 0.0001, ⍵2 = 0.118) to be statistically significant in driving community differences between samples, not site type (p = 0.298) or COI gene copy number (p = 0.881). For the subset of the dataset with nutrient data, in addition to depth (p = 0.0003, ⍵2 = 0.0612), TOC% (p = 0.0404, ⍵2 = 0.0176) was also statistically significant. Site type (p = 0.380), TC% (p = 0.270) and TN% (p = 0.142) were not statistically significant. Once again, the effect size for depth was the largest of the two significant factors, by far.

A genus-level volcano plot (Supplementary Figure E12) was made to examine potential indicator taxa that may be associated with sediment from the two different site types. However, unlike the 16S dataset, statistically significant potential indicator taxa were less prevalent across all samples. Some of the more prominent indicator taxa with p-values lower than 0.05 include *Cryomonadida*, *Heterosigma*, *Ventrifissuridae*, *Gregarines* GRE1 and *Minutocellus*, which are less enriched in sediments inside the farm than outside the farm. However, none of the above taxa are prevalent across all the samples of that site type.


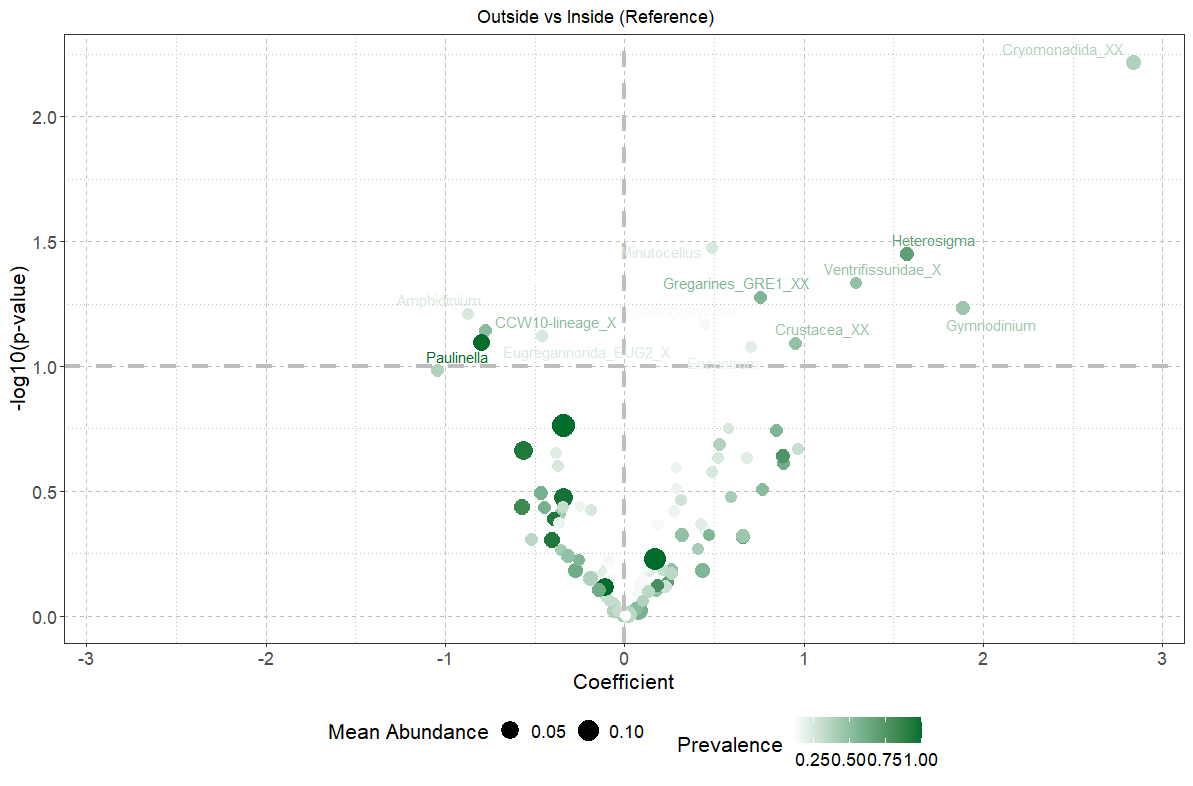


**Supplementary Figure E12:** Volcano plot of potential microbial indicator taxa at the family level distinguishing samples from inside and outside the farm in the 2021 18S dataset. Refer to Supplementary Figure E8 for details on figure interpretation.

The correlation analysis conducted on COI gene copy number (Supplementary Table E4) showed taxa such as *Lynella* and *Pelagostrobilidium* to be positively correlated with COI gene copies both inside and outside the farm. Within the farm, *Ventrifissuridae*, *Chromadorita, Discomonas* and *Halalaimus* were positively correlated with COI, while *Gonyaulax, Metschnikowia* and *Paraconiothyrium* were inversely correlated with COI. *Vetrifissuridae* was the only taxon common to both indicator taxon methods, but despite being relatively less enriched in sediments from inside the farm in the volcano plot, it showed a positive correlation with COI.

**Supplementary Table E4:** A list of the top significant taxa (p < 0.05) in the correlation analysis for the 2022 18S dataset, and their associated p-values, Spearman’s rho values and site types. Spearman's rho values range from -1 to +1, indicating negative to positive correlation respectively. Taxa are correlated with both site type and log COI values, and are sorted by site type, then p-value, and then finally rho.

| **Type** | **Genus** | **rho** | **p-value** |
| --- | --- | --- | --- |
| Inside | *Gonyaulax* | -0.68411 | 0.006969 |
| Inside | *Ventrifissuridae*_X | 0.674468 | 0.008151 |
| Inside | *Lynnella* | 0.670251 | 0.008715 |
| Inside | *Metschnikowia* | -0.62692 | 0.016419 |
| Inside | *Pelagostrobilidium* | 0.583621 | 0.028446 |
| Inside | *Paraconiothyrium* | -0.54814 | 0.042417 |
| Outside | CCW10-lineage_X | -0.6938 | 0.004118 |
| Outside | *Lynnella* | 0.627549 | 0.012262 |
| Outside | *Heterosigma* | -0.61057 | 0.015625 |
| Outside | *Heterolepidoderma* | -0.58896 | 0.020882 |
| Outside | *Pelagostrobilidium* | 0.578406 | 0.023896 |
| Outside | *Chromadorita* | 0.553399 | 0.032352 |
| Outside | *Discomonas* | 0.54719 | 0.034762 |
| Outside | *Askenasia* | -0.54394 | 0.036074 |
| Outside | *Oncholaimus* | -0.51824 | 0.047813 |
| Outside | *Halalaimus* | 0.514554 | 0.0497 |

## **Section F:** **2022 16S + 18S metabarcoding data**

### **Alpha diversity metrics: 16S and 18S**

Based on our analysis of the 2021 sampling effort, in 2022, we chose to take more samples inside the farm to better understand the level of variation within the farm sediments and focus on a subset of depths that the 2021 sampling indicated were significant. Observed diversity (OD) and the Shannon Index (SI) were calculated for both 16S and 18S datasets to examine whether the number of taxa differed between the different site types, as well as with depth and COI gene copy number. Violin plots of OD and SI by site type and depth can be found in Supplementary Figures F1-3 (16S) and F4-6 (18S). Observed diversity in this case refers to the absolute number of ASVs, while the Shannon index also accounts for the abundance of each ASV. Tabulated OD and SI values by site type and depth can be found in Supplementary Tables F1 and F2.

For the 2022 16S dataset, there were modest but statistically significant differences in alpha diversity between inside and outside the farm, which was not observed in the 2021 16S datasets. Kruskal-Wallis tests showed both OD (KW χ^2^ = 10.233, df = 1, p = 0.00109) and the SI (KW χ^2^ = 10.233, df = 1, p = 0.00138) to be statistically significant. The post-hoc Dunn’s tests showed both OD (p = 0.0005) and the SI (p = 0.0007) to be greater in samples from outside the farm. For the 2022 18S dataset, only the SI was significantly different between the two site types (KW χ^2^ = 7.955, df = 1, p = 0.000480), but not OD (KW χ^2^ = 1.644, df = 1, p = 0.200). Once again, the post-hoc Dunn’s test showed the SI to be greater from samples outside the farm (p = 0.0024).

For COI gene copy number, there was no statistically significant association in both datasets, for both indices (16S-OD: KW χ^2^ = 59, df = 59, p = 0.476; 16S-SI: KW χ^2^ = 59, df = 59, p = 0.476; 18S-OD: KW χ^2^ = 57, df = 57, p = 0.475; 18S-SI: KW χ^2^ = 57, df = 57, p = 0.475).

For the 16S dataset, there was no significant difference in OD (KW χ^2^ = 2.0367, df = 3, p = 0.565) and the SI (KW χ^2^ = 0.805, df = 3, p = 0.848) with depth, unlike in 2021, where depth was significant for the SI. Likewise, neither OD (KW χ^2^ = 2.0367, df = 3, p = 0.565) nor the SI (KW χ^2^ = 0.805, df = 3, p = 0.845) showed any significant difference with depth in the 18S dataset.

Overall, there is some indication of alpha diversity potentially being higher outside the farm than inside the farm, with both indices being statistically significant in the 16S dataset, and the SI only in the 18S dataset.


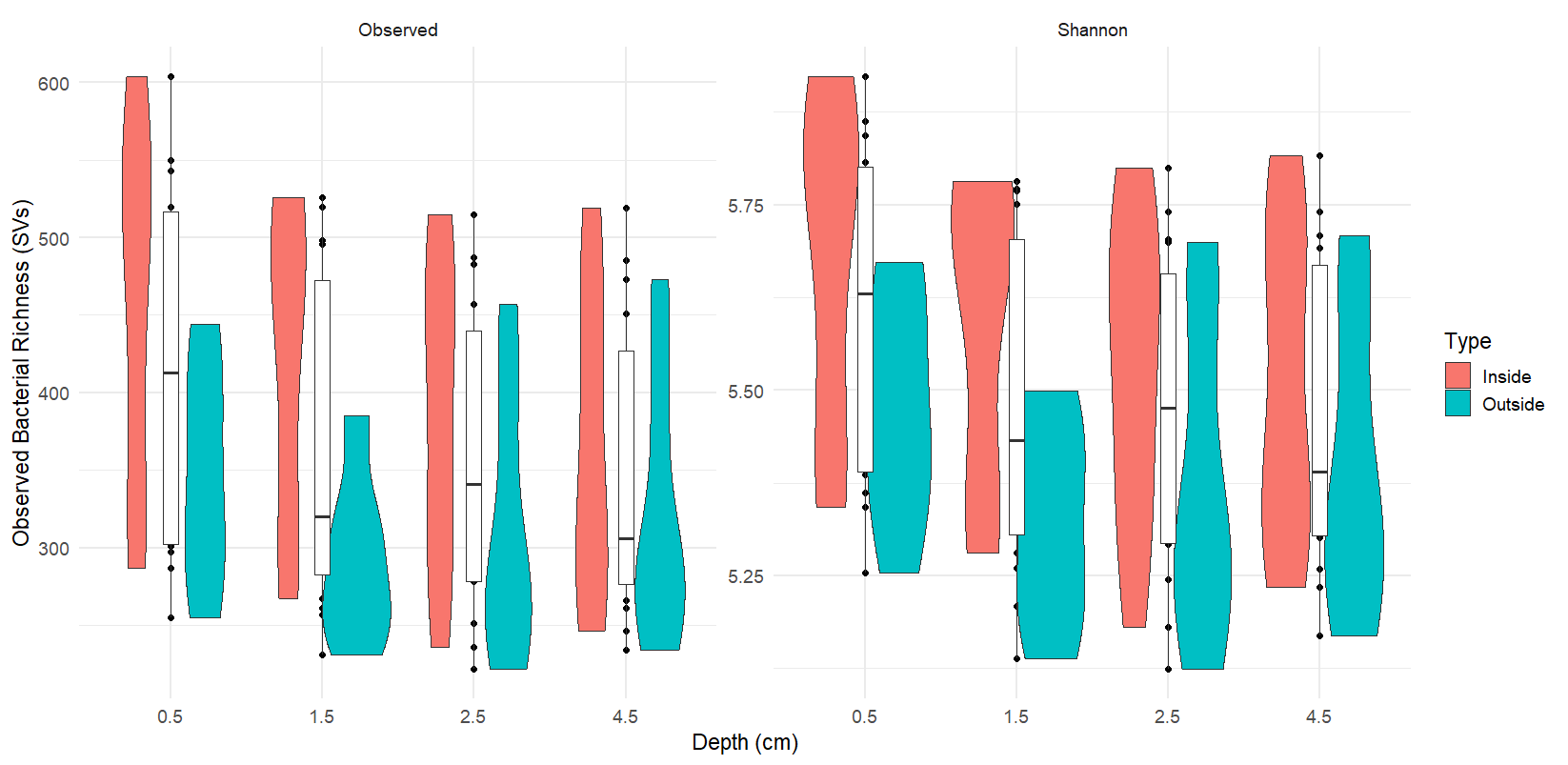


**Supplementary Figure F1:** Violin plots of alpha diversity (observed diversity and the Shannon Index) for the 2022 16S dataset, colored by site type and sorted by depth. The bar plots correspond to depth only.


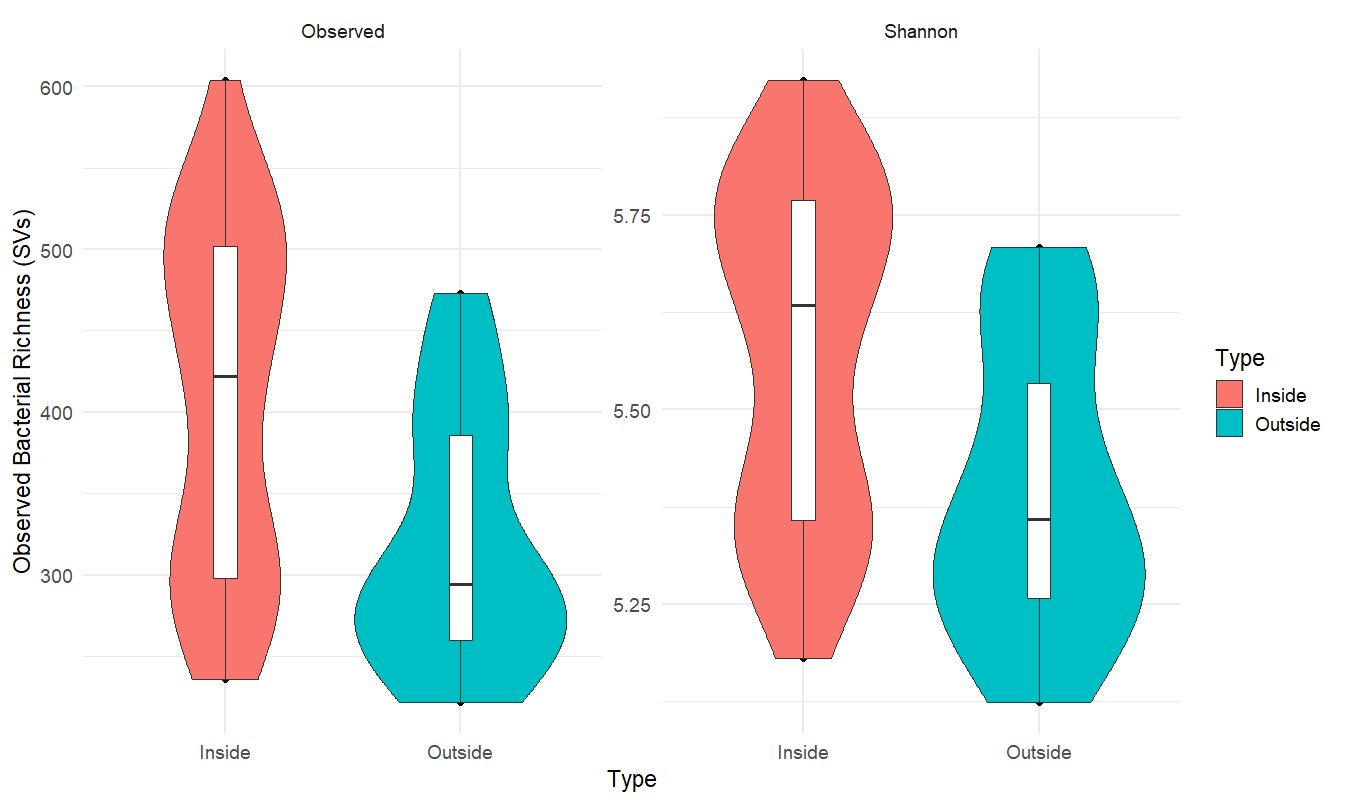


**Supplementary Figure F2:** Violin plots of alpha diversity (observed diversity and the Shannon Index) for the 2022 16S dataset, colored by site type. Sample depth has been omitted for simplicity.


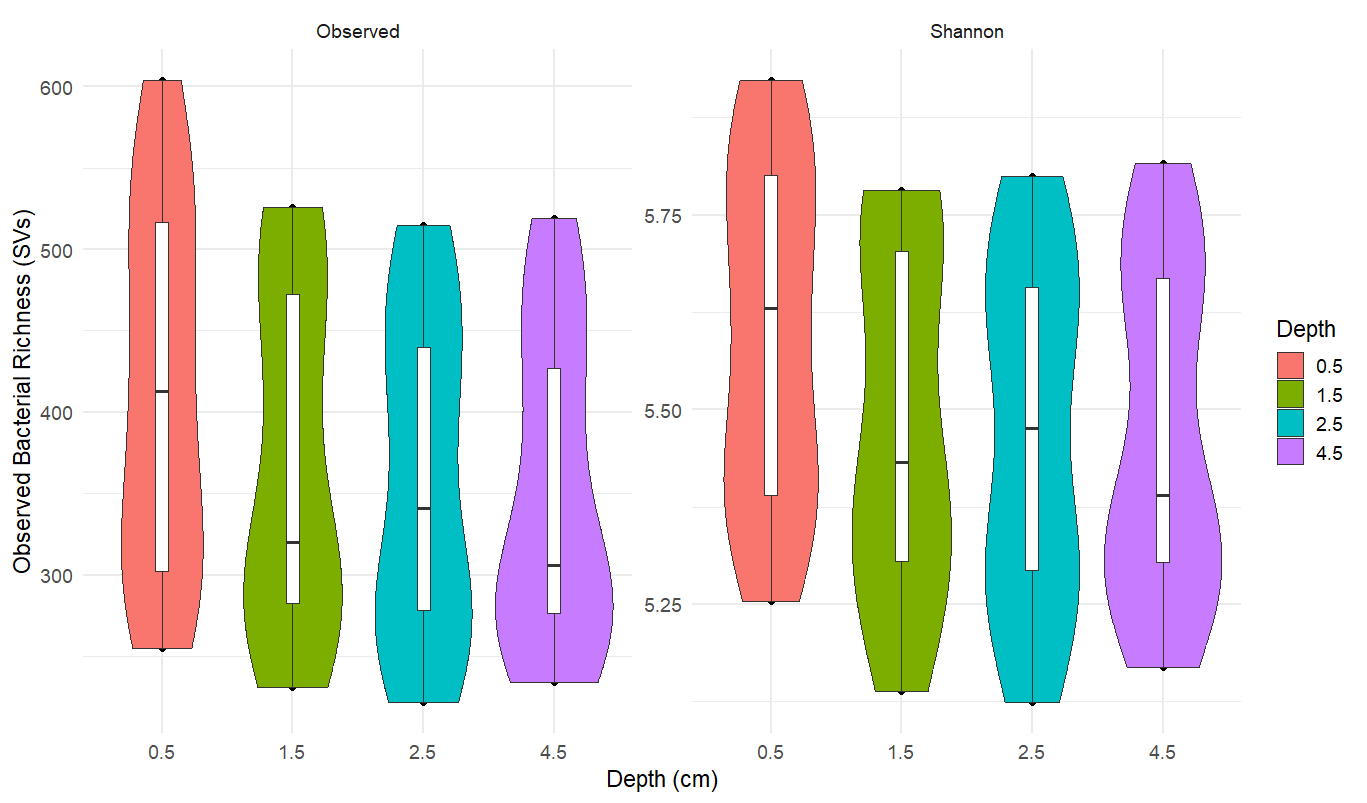


**Supplementary Figure F3:** Violin plots of alpha diversity (observed diversity and the Shannon Index) for the 2022 16S dataset, colored by sample depth. Site type has been omitted for simplicity.

**Supplementary Table F1:** Tabulated alpha diversity summary statistics (observed diversity and the Shannon Index) for the 2022 16S dataset, organized by site type and depth. Observed diversity values are given to 1 decimal place.

| Type | Depth (cm) | Observed Diversity | S.D. | Shannon Index | S.D. |
| --- | --- | --- | --- | --- | --- |
| Inside | 0.5 | 454.1 | 119.2 | 5.672 | 0.226 |
| Inside | 1.5 | 420.7 | 102.5 | 5.594 | 0.206 |
| Inside | 2.5 | 386.8 | 100.6 | 5.529 | 0.210 |
| Inside | 4.5 | 366.0 | 101.4 | 5.508 | 0.217 |
| Outside | 0.5 | 348.0 | 733 | 5.473 | 0.160 |
| Outside | 1.5 | 292.5 | 55.5 | 5.320 | 0.140 |
| Outside | 2.5 | 312.5 | 90.4 | 5.379 | 0.226 |
| Outside | 4.5 | 323.7 | 90.6 | 5.400 | 0.213 |

**
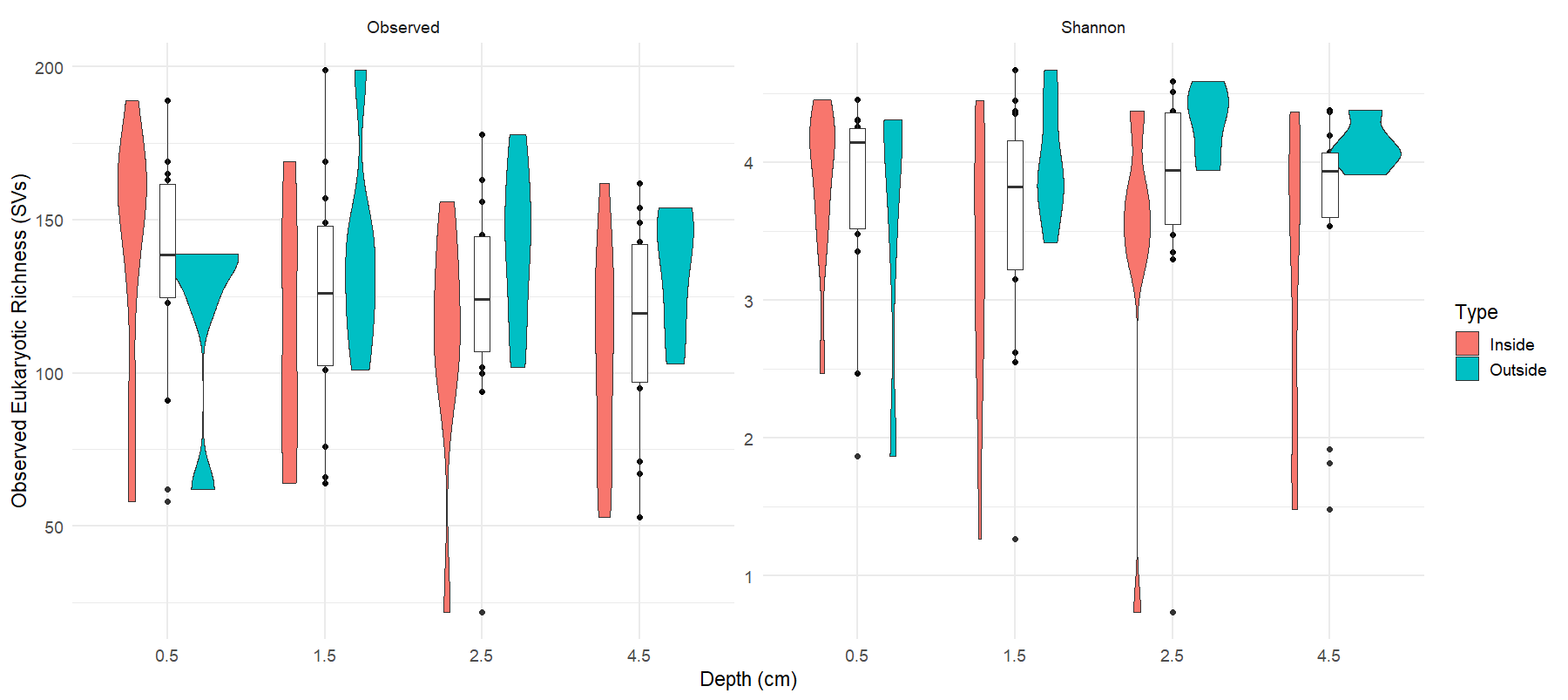
**

**Supplementary Figure F4:** Violin plots of alpha diversity (observed diversity and the Shannon Index) for the 2022 18S dataset, colored by site type and sorted by depth. The bar plots correspond to depth only.


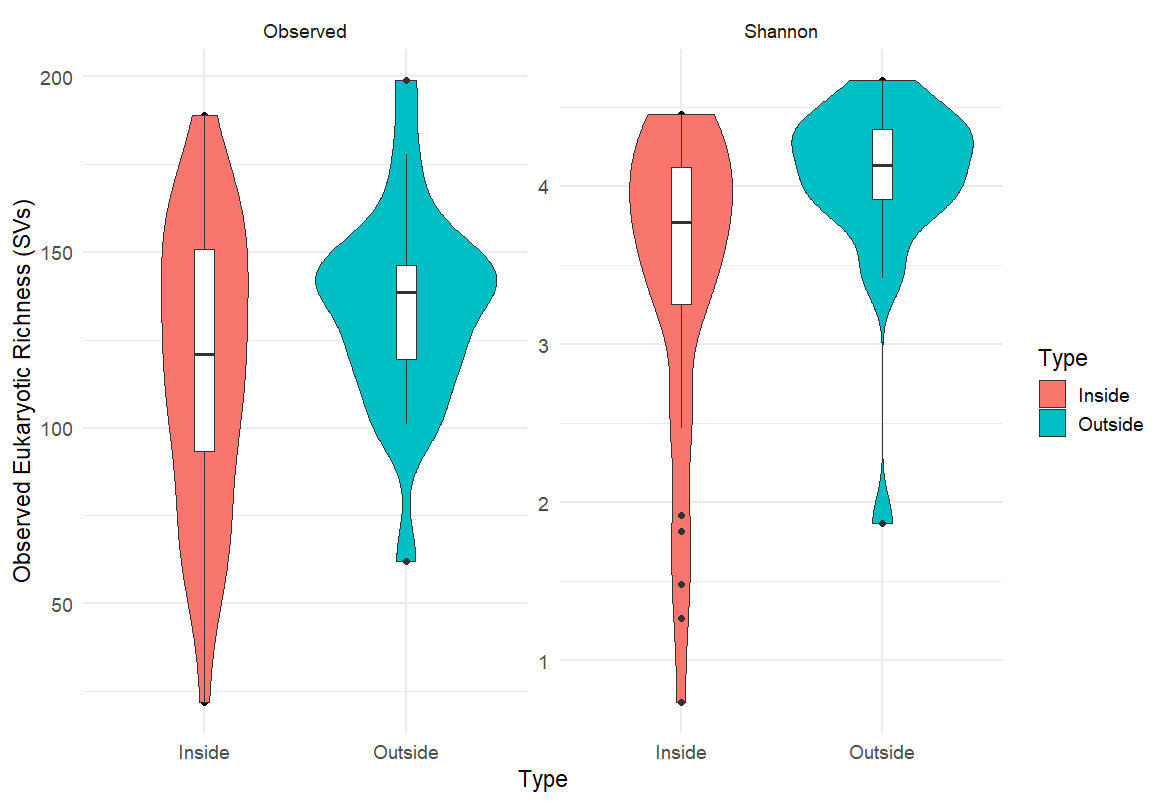


**Supplementary Figure F5:** Violin plots of alpha diversity (observed diversity and the Shannon Index) for the 2022 18S dataset, colored by site type. Sample depth has been omitted for simplicity.

**
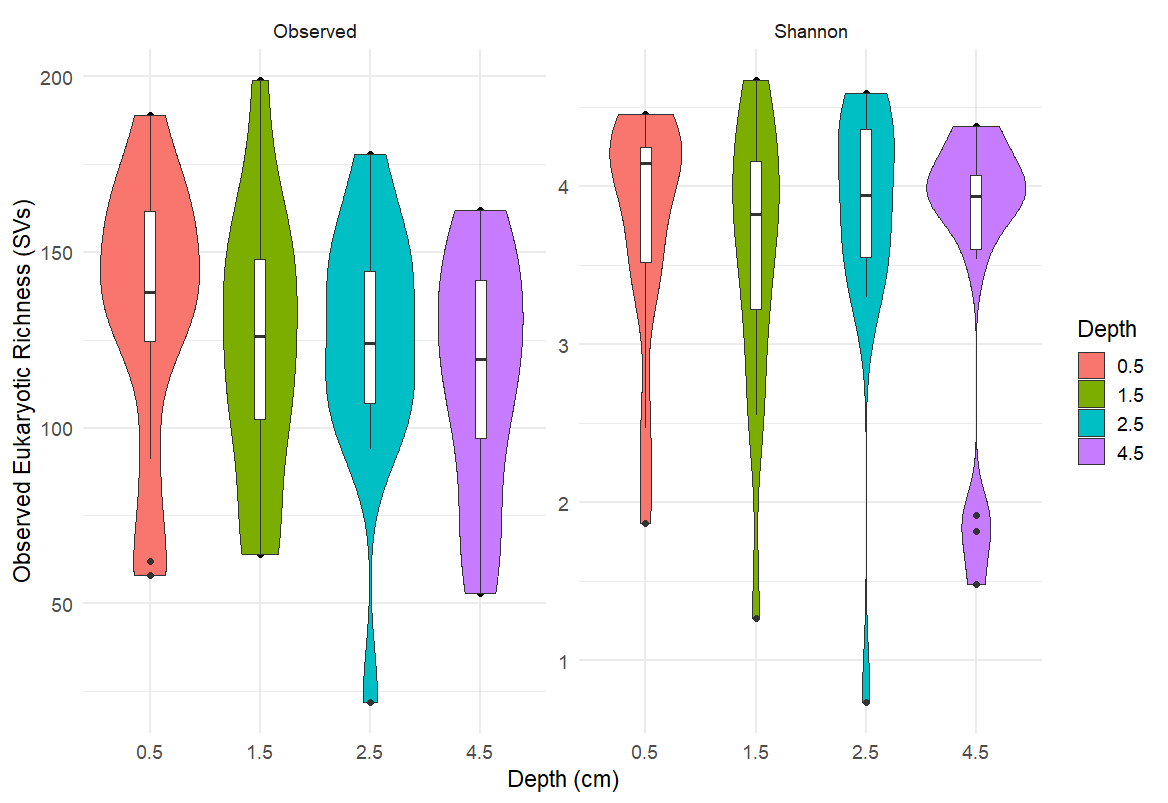
**

**Supplementary Figure F6:** Violin plots of alpha diversity (observed diversity and the Shannon Index) for the 2022 18S dataset, colored by sample depth. Site type has been omitted for simplicity.

**Supplementary Table F2:** Tabulated alpha diversity summary statistics (observed diversity and the Shannon Index) for the 2022 18S dataset, organized by site type and depth. Observed diversity values are given to 1 decimal place.

| Type | Depth (cm) | Observed Diversity | S.D. | Shannon Index | S.D. |
| --- | --- | --- | --- | --- | --- |
| Inside | 0.5 | 141.1 | 41.8 | 3.830 | 0.615 |
| Inside | 1.5 | 113.7 | 40.0 | 3.288 | 1.027 |
| Inside | 2.5 | 111.4 | 39.2 | 3.416 | 1.074 |
| Inside | 4.5 | 105.6 | 36.5 | 3.197 | 1.118 |
| Outside | 0.5 | 120.0 | 33.1 | 3.632 | 1.043 |
| Outside | 1.5 | 138.5 | 34.0 | 4.002 | 0.450 |
| Outside | 2.5 | 141.7 | 27.8 | 4.300 | 0.256 |
| Outside | 4.5 | 134.0 | 21.4 | 4.120 | 0.177 |


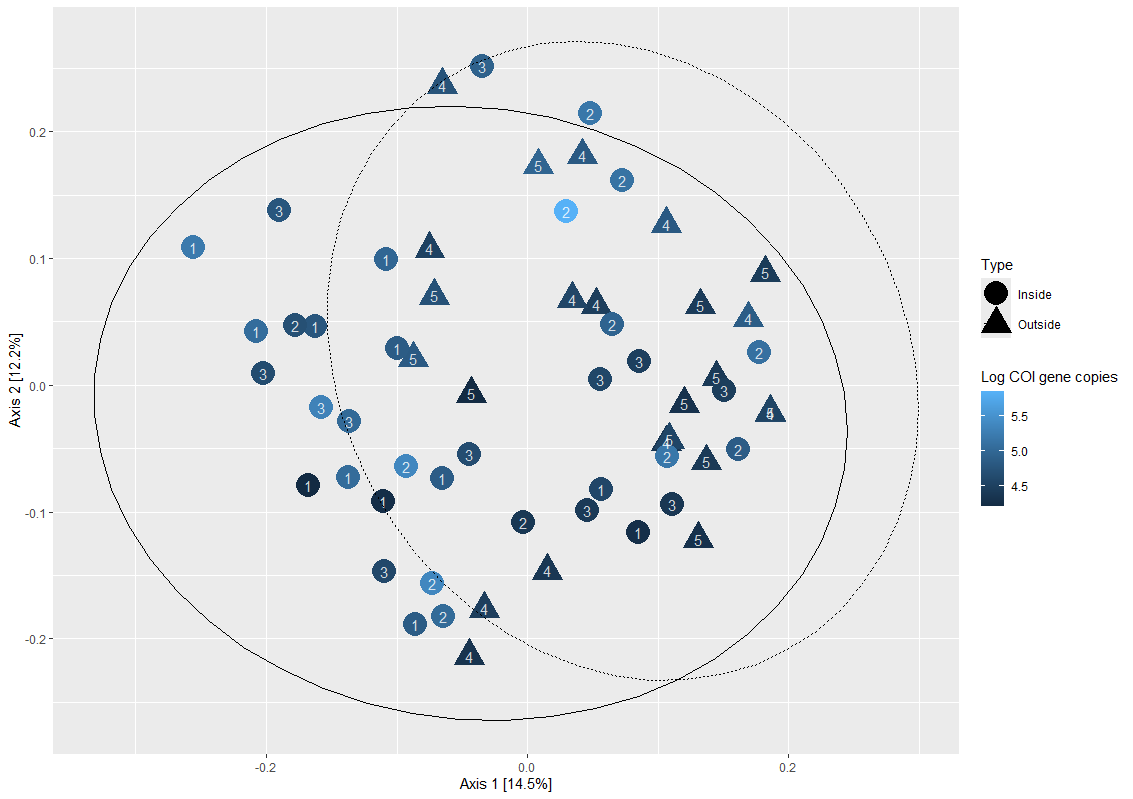


**Supplementary Figure F7:** Alternative principal coordinates analysis (PCoA) plot of the 2022 16S microbiome dataset, based on Bray-Curtis distances. Each point represents a single sample, with shape representing site type (inside vs outside the farm) and color representing log *S. latissima* COI gene copies, with lighter colors representing greater values. Site number (1 to 5) is overlaid on top of each point. Ellipses have been drawn around points of the two site types at the 95% confidence interval; with the ellipse with solid lines representing samples from inside the farm, and the ellipse with dotted lines representing samples from outside the farm. The PCoA colored based on depth can be found as Figure 5.


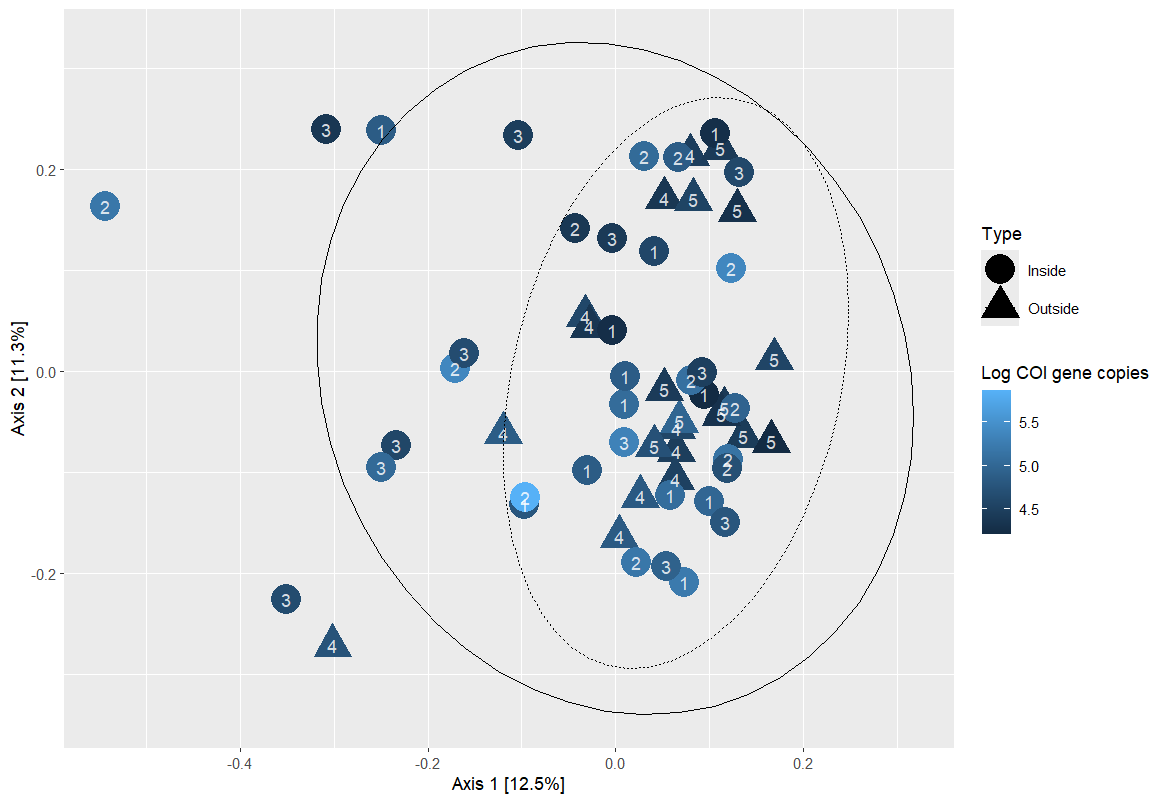


**Supplementary Figure F8:** Alternative principal coordinates analysis (PCoA) plot of the 2022 18S eukaryotic dataset, based on Bray-Curtis distances. See Supplementary Figure F7 for details on figure interpretation. The PCoA colored based on depth can be found as Figure 8.

**Supplementary Table F3:** A list of the top significant taxa (p < 0.05) in the correlation analysis for the 2022 16S dataset, and their associated p-values, Spearman’s rho values and site types. Spearman's rho values range from -1 to +1, indicating negative to positive correlation respectively. Taxa are correlated with both site type and log COI values, and are sorted by site type, then p-value, and then finally rho.

| **Type** | **Family** | **rho** | **p-value** |
| --- | --- | --- | --- |
| Inside | koll11 | -0.49708 | 0.002041 |
| Inside | BSV26 | -0.48876 | 0.002489 |
| Inside | *Arenicellaceae* | -0.4883 | 0.002516 |
| Inside | *Thermoanaerobaculaceae* | -0.44881 | 0.006041 |
| Inside | *Rhodothermaceae* | 0.43463 | 0.008077 |
| Inside | AB-539-J10 | -0.42378 | 0.010006 |
| Inside | AKAU3564 sediment group | -0.42306 | 0.010147 |
| Inside | *Rubritaleaceae* | 0.398318 | 0.016125 |
| Inside | *Flavobacteriaceae* | 0.387412 | 0.019573 |
| Inside | *Gemmatimonadaceae* | -0.38076 | 0.021965 |
| Inside | *Prolixibacteraceae* | 0.375098 | 0.024185 |
| Inside | *Rhizobiales Incertae Sedis* | -0.36821 | 0.027135 |
| Inside | *Sumerlaeaceae* | -0.36676 | 0.027791 |
| Inside | *Nitrosomonadaceae* | 0.362354 | 0.02987 |
| Inside | *Nitrincolaceae* | 0.362322 | 0.029886 |
| Inside | *Rubinisphaeraceae* | 0.350114 | 0.036321 |
| Inside | *Sedimenticolaceae* | -0.34914 | 0.03688 |
| Inside | *Fermentibacteraceae* | -0.34438 | 0.039708 |
| Inside | *Crocinitomicaceae* | 0.338081 | 0.043725 |
| Inside | *Spirochaetaceae* | -0.33765 | 0.044011 |
| Inside | *Ectothiorhodospiraceae* | -0.33318 | 0.047067 |
| Outside | *Granulosicoccaceae* | 0.68816 | 0.000202 |
| Outside | *Nitrincolaceae* | 0.645448 | 0.000659 |
| Outside | *Flavobacteriaceae* | 0.644488 | 0.000675 |
| Outside | *Kangiellaceae* | 0.627403 | 0.001032 |
| Outside | *Latescibacteraceae* | -0.59861 | 0.002 |
| Outside | *Rhodobacteraceae* | 0.579269 | 0.003015 |
| Outside | *Rhizobiaceae* | 0.560264 | 0.004408 |
| Outside | *Anaerolineaceae* | -0.55055 | 0.005307 |
| Outside | *Desulfosarcinaceae* | -0.54316 | 0.00609 |
| Outside | *Ectothiorhodospiraceae* | -0.52437 | 0.008528 |
| Outside | SB-5 | -0.51774 | 0.00956 |
| Outside | *Alteromonadaceae* | 0.514519 | 0.010099 |
| Outside | *Thermoanaerobaculaceae* | -0.51272 | 0.01041 |
| Outside | *Halieaceae* | 0.510991 | 0.010717 |
| Outside | *Rubinisphaeraceae* | 0.51042 | 0.01082 |
| Outside | *Methyloligellaceae* | -0.50968 | 0.010954 |
| Outside | *Amoebophilaceae* | 0.503737 | 0.012086 |
| Outside | *Saprospiraceae* | 0.498585 | 0.013143 |
| Outside | *AB-539-J10* | -0.49366 | 0.014223 |
| Outside | Unknown Family | 0.489343 | 0.015229 |
| Outside | *Moduliflexaceae* | -0.4889 | 0.015336 |
| Outside | SG8-4 | -0.47052 | 0.02032 |
| Outside | 67-14 | -0.46627 | 0.021639 |
| Outside | *Cryomorphaceae* | 0.462386 | 0.022907 |
| Outside | *Crocinitomicaceae* | 0.461572 | 0.02318 |
| Outside | *Alicyclobacillaceae* | -0.45873 | 0.024154 |
| Outside | *Rubritaleaceae* | 0.450939 | 0.026994 |
| Outside | Hyphomicrobiaceae | -0.45021 | 0.027274 |
| Outside | ST-12K33 | 0.445696 | 0.029052 |
| Outside | Desulfobulbaceae | -0.44333 | 0.03002 |
| Outside | BSV26 | -0.41956 | 0.041252 |
| Outside | Arenicellaceae | -0.4051 | 0.04956 |

**Supplementary Table F4:** A list of the top significant taxa (p < 0.05) in the correlation analysis for the 2022 18S dataset, and their associated p-values, Spearman’s rho values and site types. See Supplementary Table F3 for details on data interpretation.

| Type | Genus | rho | p-value |
| --- | --- | --- | --- |
| Inside | *Rimostrombidium*_A | -0.49606 | 0.002092 |
| Inside | *Gastrotricha*_XXX | 0.451415 | 0.005719 |
| Inside | *Pycnophyes* | 0.444248 | 0.006641 |
| Inside | *Oxystomina* | 0.416703 | 0.011466 |
| Inside | *Spongospora* | -0.40219 | 0.015031 |
| Inside | *Thalassiosira* | 0.395882 | 0.016847 |
| Inside | *Scrippsiella* | -0.38048 | 0.022069 |
| Inside | *Amylax* | -0.3708 | 0.025994 |
| Inside | *Difficilina* | 0.359726 | 0.031169 |
| Inside | *Halomonhystera* | 0.350632 | 0.036026 |
| Inside | *Parastrombidinopsis* | 0.340289 | 0.042282 |
| Inside | *Oncholaimus* | 0.33242 | 0.047606 |
| Outside | *Scrippsiella* | -0.61459 | 0.002339 |
| Outside | *Protodinium* | -0.61435 | 0.002351 |
| Outside | *Biecheleria* | -0.61204 | 0.002468 |
| Outside | *Filosa-Imbricatea*_XXX | -0.58041 | 0.004626 |
| Outside | *Ptycholaimellus* | 0.563473 | 0.006318 |
| Outside | *Zostera* | -0.54741 | 0.008367 |
| Outside | *Pentapharsodinium* | -0.5312 | 0.010962 |
| Outside | *Chromadorita* | 0.487419 | 0.021396 |
| Outside | *Skeletonema* | -0.47205 | 0.026544 |
| Outside | *Amylax* | 0.467262 | 0.028336 |
| Outside | *Tintinnopsis*_05 | -0.46636 | 0.028684 |
| Outside | *Marimonadida*_XX | -0.46581 | 0.0289 |
| Outside | CONTH_7_XXX | -0.46015 | 0.031172 |
| Outside | *Heteromastus* | 0.459536 | 0.031428 |
| Outside | *Gymnodinium* | -0.45875 | 0.031757 |
| Outside | *Gregarina*_1 | -0.4486 | 0.036254 |
| Outside | *Verrucomonas* | -0.44299 | 0.038946 |
| Outside | *Pseudopirsonia* | -0.44149 | 0.039693 |
| Outside | *Ovulinata* | -0.42772 | 0.047067 |
| Outside | *Odontophora* | 0.42351 | 0.049525 |
| Outside | *Paraconiothyrium* | -0.42326 | 0.049671 |
